# A Masked PY-NLS in *Drosophila* TIS11 and Its Mammalian Homolog Tristetraprolin

**DOI:** 10.1371/journal.pone.0071686

**Published:** 2013-08-09

**Authors:** Laure Twyffels, Corinne Wauquier, Romuald Soin, Christine Decaestecker, Cyril Gueydan, Véronique Kruys

**Affiliations:** 1 Laboratoire de Biologie Moléculaire du Gène, Faculté des Sciences, Université Libre de Bruxelles (ULB), Gosselies, Belgium; 2 Center for Microscopy and Molecular Imaging, Gosselies, Belgium; 3 Laboratory of Image Synthesis and Analysis - Ecole Polytechnique de Bruxelles, Université Libre de Bruxelles (ULB), Brussels, Belgium; German Cancer Research Center, Germany

## Abstract

Many RNA-binding proteins (RBPs) dynamically shuttle between the nucleus and the cytoplasm, often exerting different functions in each compartment. Therefore, the nucleo-cytoplasmic distribution of RBPs has a strong impact on their activity. Here we describe the localization and the shuttling properties of the tandem zinc finger RBP dTIS11, which is the *Drosophila* homolog of mammalian TIS11 proteins. *Drosophila* and mammalian TIS11 proteins act as destabilizing factors in ARE-mediated decay. At equilibrium, dTIS11 is concentrated mainly in the cytoplasm. We show that dTIS11 is a nucleo-cytoplasmic shuttling protein whose nuclear export is mediated by the exportin CRM1 through the recognition of a nuclear export signal (NES) located in a different region comparatively to its mammalian homologs. We also identify a cryptic Transportin-dependent PY nuclear localization signal (PY-NLS) in the tandem zinc finger region of dTIS11 and show that it is conserved across the TIS11 protein family. This NLS partially overlaps the second zinc finger ZnF2. Importantly, mutations disrupting the capacity of the ZnF2 to coordinate a Zinc ion unmask dTIS11 and TTP NLS and promote nuclear import. All together, our results indicate that the nuclear export of TIS11 proteins is mediated by CRM1 through diverging NESs, while their nuclear import mechanism may rely on a highly conserved PY-NLS whose activity is negatively regulated by ZnF2 folding.

## Introduction

The nucleo-cytoplasmic compartmentalization enables eukaryotic cells to develop sophisticated mechanisms to regulate gene expression at post-transcriptional levels. Messenger RNAs undergo several maturation processes in the nucleus before being exported to the cytoplasm, where they are dispatched to specialized machineries that finally translate and degrade them. All these processes are mediated by RNA-binding proteins (RBPs), many of which are able to shuttle between the nucleus and the cytoplasm. Post-transcriptional regulations can therefore be rapidly and efficiently modulated by changes in RBP localization. Numerous studies illustrate how the conditional translocation of a specific RBP, for example Hur [1] or hnRNPA1 [2], can affect its target mRNAs.

Nucleo-cytoplasmic traffic occurs through the nuclear pore complexes (NPCs). These large structures that span the nuclear envelope provide a diffusion channel for small molecules and also enable active transport of molecules ranging from single proteins to ribosomal subunits and mRNA export complexes. Active transport of proteins is mediated by receptors belonging to the karyopherin β family, which includes about twenty members in mammals [3]. These factors recognize nuclear localization signals (NLS) or nuclear export signals (NES) in their protein cargoes and convey them through the NPC. The GTPase Ran regulates karyopherin β-cargo interactions, and the directionality of the karyopherin β-mediated transfers is ensured by the presence of a nuclear Ran-GTP vs a cytoplasmic Ran-GDP gradient across the nuclear envelope [4]–[7]. By contrast, bulk mRNA export is mediated by the non-karyopherin β transporter NXF1/TAP and is facilitated by a host of RNA-binding proteins associated with the mRNA molecule. The directionality of the transport here relies on the remodeling of the mRNP on the cytoplasmic side of the NPC [8].

The RNA-binding protein dTIS11 is the single member of the TIS11 family present in *Drosophila*. TIS11 proteins consist of a conserved tandem zinc finger domain (TZF) flanked by divergent amino- and carboxy-terminal regions. The two zinc fingers are 18 amino acids apart and each is initiated by an R/K-Y-K-T-E sequence preceding the C-X_8_-C-X_5_-C-X_3_-H motif. Apart from the TZF, the only region conserved through evolution is the carboxy-terminal extremity. In mammals, the TIS11 protein family comprises four members: TTP (Tristetraprolin, also called TIS11/ZFP36/NUP475), TIS11B (also called BRF-1/ERF-1/ZFP36L1/CMG1), TIS11D (also called BRF-2/ERF-2/ZFP36L2) and ZFP36L3 [9], [10]. The latter has only been detected in murine placenta and extra-embryonic tissues and seems to be rodent-specific [11]. TTP is well known for being able to bind adenine- and uridine-rich elements (AREs) that are found in the 3’ UTR of numerous mRNAs, thereby inducing their deadenylation and decay. Its targets include TNF [12], GM-CSF [13] and IL-3 [14] mRNAs. AREs can also be recognized by TIS11B, TIS11D and ZFP36L3 [9]–[11]. Recently, we and others showed that like mammalian TIS11 proteins, dTIS11 binds to AREs and accelerates the deadenylation and decay of ARE-containing mRNAs. Among its targets is the mRNA encoding the anti-microbial peptide Cecropin A1 [15]–[18].

Mammalian TTP, TIS11B and TIS11D proteins are known to continuously shuttle between the nucleus and the cytoplasm. Their export from the nucleus is mediated by the general export factor CRM1 [19], [20]. TTP was also shown to interact with the nucleoporin CAN/Nup214, suggesting an alternative NES-independent nuclear export mechanism [21]. The nuclear accumulation of TTP and TIS11B has been shown to require the TZF domain but not the RNA-binding ability [19], [20]. However, it has not been demonstrated that the entry of these proteins into the nucleus is an active process and no transporter has yet been identified. The transcription of the mammalian member *TTP* can be induced by the same signals activating transcription of some of its mRNA targets [12]. Furthermore, functional AREs are found in the 3’UTR of mammalian TIS mRNAs [22]. This mechanism generates a negative feedback loop ensuring a temporally restricted pattern of expression for TIS11 proteins and their targets. By contrast, *dtis11* gene is constitutively expressed in *Drosophila* S2 cells and does not contain ARE motifs [16]. These observations suggest that in S2 cells, the control of dTIS11 activity could rely mainly on the regulation of its subcellular localization and/or on post-translational modifications rather than on expression-regulating mechanisms that evolved later in evolution.

In the present study, we characterized the determinants and the machinery governing the nucleo-cytoplasmic distribution of dTIS11. Our results bring new insights into the mechanism ruling the nucleo-cytoplasmic shuttling of TIS11 proteins and provide evidence for similarities and differences between *Drosophila* and mammalian TIS11 protein trafficking.

## Results

### dTIS11 is a nucleo-cytoplasmic shuttling protein whose nuclear export is mediated by CRM1 exportin

**Figure 1:**
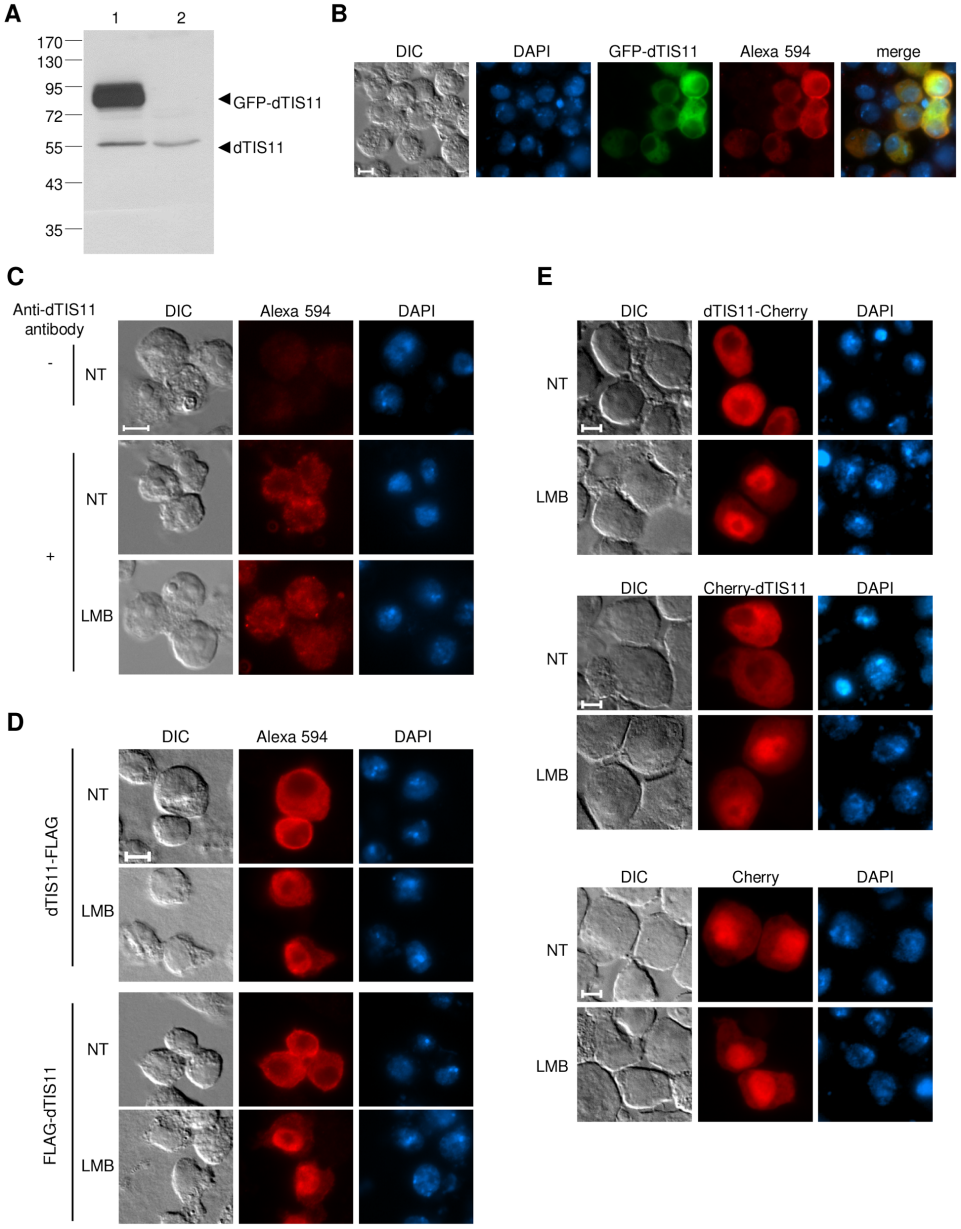
dTIS11 is cytoplasmic and relocalizes to the nucleus in response to leptomycin B. **(**A) Western blot analysis of endogenous dTIS11 and GFP-dTIS11 expression in S2 cells with the dTIS11 monoclonal antibody. (B) Detection of GFP-dTIS11 in transiently transfected S2 cells by direct fluorescence of GFP (green) or immunostaining with the murine monoclonal anti-dTIS11 antibody and an Alexa 594 anti-mouse secondary antibody (red) as described in Material and Methods. The nuclei were stained with DAPI (blue). (C) Endogenous dTIS11 subcellular localization in S2 cells treated or not with leptomycin B (10 ng/ml for 5h). (D) Localization of dTIS11-FLAG and FLAG-dTIS11 in S2 cells treated or not with leptomycin B (10 ng/ml for 5h) (E) Localization of dTIS11-Cherry, Cherry-dTIS11 and unfused Cherry in S2 cells-treated or not with leptomycin B (10 ng/ml for 5h). Bar  =  5 µm.

To study dTIS11 localization, we isolated a monoclonal antibody from mice immunized with purified dTIS11. This antibody specifically detects both endogenous dTIS11 and ectopically expressed GFP-dTIS11 in western blot (Fig. 1A). As expected, the level of expression of the endogenous protein appears considerably lower than that of GFP-dTIS11. The antibody also recognizes GFP-dTIS11 in immunofluorescence experiments (Fig. 1B). In untransfected S2 cells, the signal provided by the anti-dTIS11 antibody is predominantly located in the cytoplasm. Upon treatment of the cells with leptomycin B (LMB), an inhibitor of the CRM1 exportin [23], [24], it is partially redistributed into the nucleus (Fig. 1C). This result suggests that dTIS11, although mainly cytoplasmic at steady state, is a nucleo-cytoplasmic shuttling protein whose nuclear export is mediated by a CRM1-dependent pathway.

We then expressed dTIS11 fused at its N- or C-terminus to the FLAG tag or to the Cherry fluorescent protein in S2 cells. The four chimeric constructs were mostly cytoplasmic in untreated cells but shifted to a more nuclear localization upon LMB treatment (Fig. 1D,E), as observed for the endogenous protein. In contrast, unfused Cherry was slightly more nuclear than cytoplasmic regardless of the LMB treatment (Fig. 1E). We then verified that the Cherry-dTIS11 fusion was able to destabilize an ARE-bearing reporter mRNA (Fig. S1), as previously shown for GFP-dTIS11 [16]. Having established that it was functional and localized similarly to the endogenous protein, we used the Cherry-dTIS11 fusion to further investigate the nucleo-cytoplasmic shuttling of dTIS11.

### The 101-113 amino acid sequence of dTIS11 is a CRM1-dependent NES

**Figure 2:**
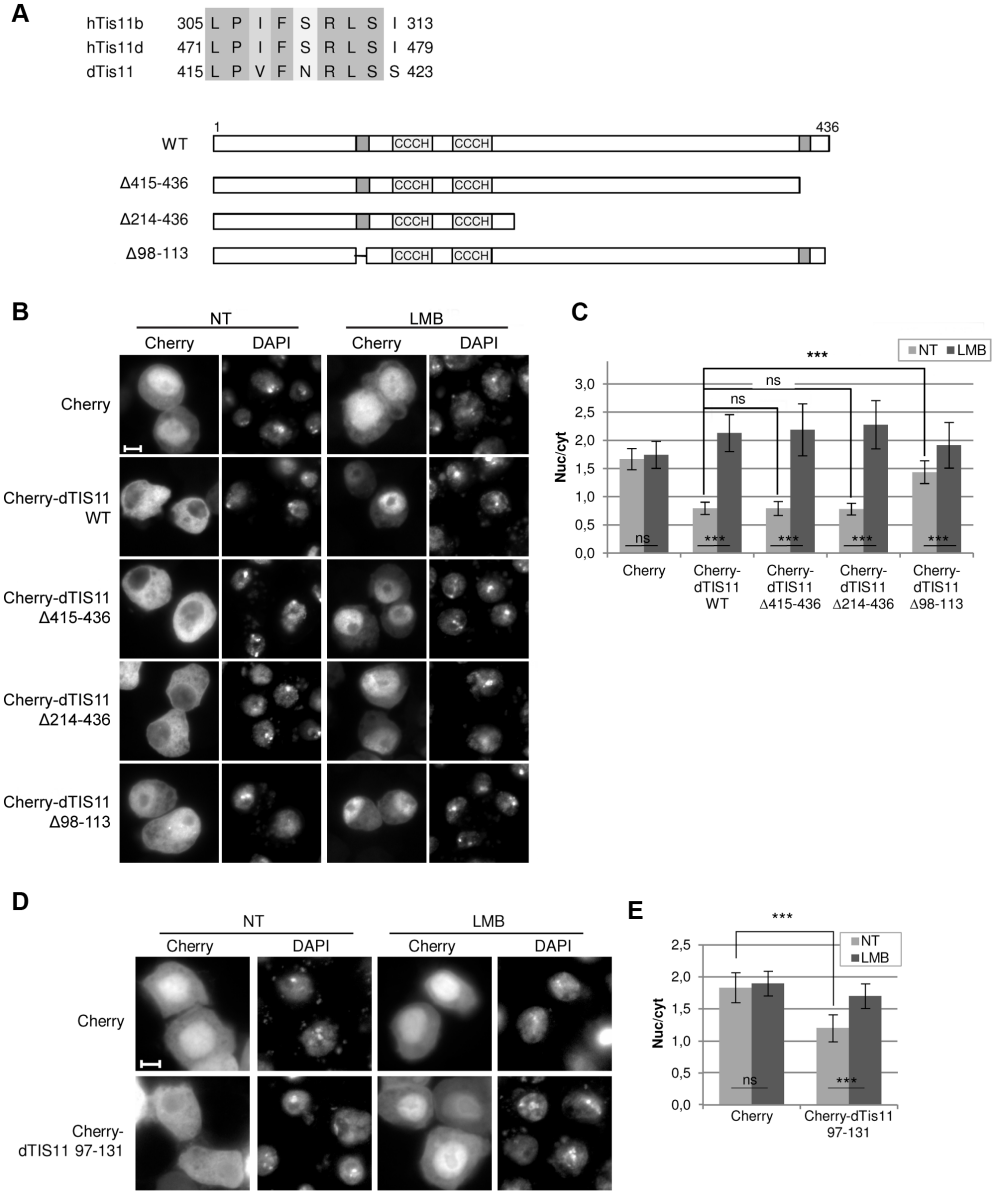
Amino acid 101-113 sequence of dTIS11 is a CRM1-dependent NES. (A) Upper panel: alignment of hTIS11B and hTIS11D NESs with the corresponding sequence in dTIS11. Lower panel: schematic representation of dTIS11 and dTIS11 deletion mutants. Light grey box « CCCH »: zinc finger domain, dark grey box: putative NESs. (B) Localization of wild-type and mutant forms of dTIS11 fused to Cherry in *Drosophila* S2 cells treated (right) or not (left) with LMB (10 ng/ml for 5h). Bar  =  5 µm. (C) Quantification of the relocalization of the various mutants in response to LMB for the experiment presented in (B). For each condition, the average fluorescence intensity was measured in the nucleoplasm (nuc) and in the cytoplasm (cyt) of 30–40 cells. Statistical analyses were performed as described in the methods section. Bars show the average of the nuc/cyt ratio ± s.d. ***: p<0.0001; ns: non-significant (p>0.05) (t-tests). Cherry-dTIS11 is also significantly more nuclear than Cherry alone in LMB-treated cells (p<0.0001). The significant difference between Cherry-dTIS11 and Cherry-dTIS11 Δ98–113 nuc/cyt ratios in untreated S2 cells was observed in 3 independent experiments. (D) S2 cells were transfected to express Cherry or Cherry-dTIS11 97-131. Sixteen hours later, cells were incubated in the presence (LMB) or the absence (NT) of LMB (10 ng/ml for 5h) before analysis by fluorescence microscopy. Bar  =  5 µm. (E) Quantification of the relocalization of the proteins in the experiment presented in (D). For each condition, the nuc/cyt ratio was quantified as described for (D). Bars show the average of the nuc/cyt ratio ± s.d. ***: p<0.0001; ns: non-significant (p>0.05) (U-tests). The significant difference between Cherry and Cherry-dTIS11 97-131 nuc/cyt ratios was observed in 2 independent experiments.

The C-terminal extremity of dTIS11 shares a high degree of identity with that of human TIS11B and TIS11D proteins (Fig. 2A, upper panel), whose nuclear export is mediated by a NES present in this region [19]. To test if this motif was responsible for the CRM1-mediated export of dTIS11, we generated DNA constructs encoding wild-type or mutant versions of dTIS11 fused to Cherry (Fig. 2A, lower panel) and expressed them in S2 cells. We observed that mutants of dTIS11 lacking the C-terminal extremity (Cherry-dTIS11 Δ415–436) or the whole C-terminal region (Cherry-dTIS11 Δ214–436) accumulated almost exclusively in the cytoplasm of untreated S2 cells as observed for the wild-type dTIS11 (Cherry-dTIS11 WT). Moreover, upon LMB treatment, these mutants were relocalized to the nucleus to the same extent as Cherry-dTIS11 WT (Fig. 2B,C). Similar results were obtained when wild-type or mutant dTIS11 were fused to the V5 tag instead of Cherry (Fig. S2A,B). Together, these results indicate that CRM1-mediated nuclear export of dTIS11 is not governed by the C-terminal region.

We then searched dTIS11 for a sequence fitting the consensus for CRM1-dependent NESs [Φ-X_2_-Φ-X_3_-Φ-X_2,3_-Φ-X-Φ] or [Φ-P/L-X-Φ-X_2_-Φ-X-Φ], where Φ is a hydrophobic residue and X is any amino acid [25]–[27]. Only one motif, found directly upstream of the ZnF domain and encompassing amino acids 101 to 113 (**L**VT**I**TEN**L**GN**M**N**L**), fulfilled these criteria. We observed that a mutant of dTIS11 lacking the a.a. 98 to 113 (Cherry-dTIS11 Δ98–113) is significantly more nuclear than the wild-type protein in untreated cells (Fig. 2B,C), which suggests that this motif indeed corresponds to a NES. In accordance with this result, the V5-tagged dTIS11 mutant lacking the first 110 amino acids is clearly more nuclear than the wild-type protein in untreated cells (Fig. S2A,B). Of note, the nuclear relocalization of the Cherry-dTIS11 Δ98–113 slightly increases upon LMB treatment. This weak but significant effect could be explained by the existence of a residual weak CRM1-dependent NES in this mutant. Alternatively, this effect might result from indirect effects of LMB leading to an increased retention of dTIS11 protein in the nucleus.

As the consensus described for CRM1-dependent NESs highlights the functional importance of hydrophobic residues, we examined the effect of point-mutating the L108 or M111 and L113 residues in dTIS11 NES (Fig. S2C). We observed that these mutations significantly increased the nuclear accumulation of the protein in untreated cells and markedly reduced the effect of LMB (Fig. S2D,E), which confirms that hydrophobic residues are necessary for efficient recognition of dTIS11 NES by CRM1 exportin.

To further test the functionality of the identified NES, we transfected S2 cells with a vector encoding mCherry fused to dTIS11 amino acids. 97–131. We observed that these 35 amino acids significantly reduced the nuclear fraction of mCherry and conferred to mCherry a LMB-sensitive localization (Fig. 2D,E). The sequence identified is thus sufficient to induce the nuclear export of a reporter protein. All together, these results demonstrate that dTIS11 nuclear export is mainly mediated by a CRM1-dependent NES located between a.a. 101 and 113.

### dTIS11 nuclear accumulation relies on an ATP-dependent mechanism

**Figure 3:**
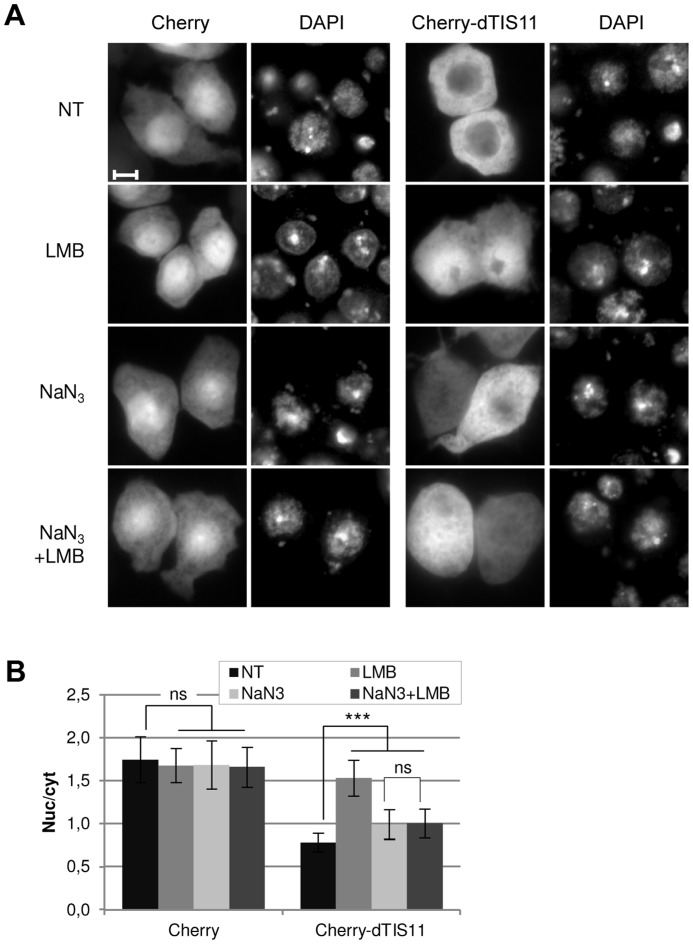
dTIS11 nuclear accumulation relies on an active energy-dependent mechanism. (A) Subcellular distribution of Cherry and Cherry-dTIS11 proteins upon energy depletion by sodium azide (10 mM, 2h) in the presence or the absence of leptomycin B (10 ng/ml). Bar  =  5 µm. (B) Quantification and statistical significance of the data shown in (A). The analysis was performed as in Fig.2 for 30–40 cells per condition. Bars show the average of the nuc/cyt ratio ± s.d. ***: p<0.0001; ns: non-significant (p>0.05) (U-tests). The results are representative of two independent experiments.

As Cherry-dTIS11 is more nuclear than Cherry in LMB-treated cells (Fig. 2C, S2E), we suspected that dTIS11 could be actively imported into the nucleus. To test this hypothesis, we determined dTIS11 localization under azide-mediated inhibition of active nucleo-cytoplasmic transport. Azide blocks the electron transport chain, drastically reducing ATP production. ATP depletion results in GTP shortage, which eventually destabilizes the Ran-GTP/Ran-GDP gradient across the nuclear envelope and thus knocks down Ran-dependent nucleo-cytoplasmic transport pathways [28]. We treated S2 cells with sodium azide and/or leptomycin B and examined where Cherry and Cherry-dTIS11 localized in these conditions. To minimize the toxicity, the treatment was restricted to 2 hours. We observed that upon azide treatment, the nuclear/cytoplasmic ratio of the fusion protein increased significantly, but stayed significantly inferior to the value obtained following LMB treatment alone. Moreover, the addition of LMB to azide-treated cells did not further affect Cherry-dTIS11 nuclear/cytoplasmic ratio (Fig. 3A,B). None of the treatments changed the nuclear/cytoplasmic ratio of unfused Cherry, confirming that the observed effects resulted from dTIS11 presence in the fusion protein (Fig. 3A,B). We can therefore assume that azide efficiently blocks dTIS11 nuclear export but inhibits dTIS11 import as well, thereby resulting in a homogenous localization. This experiment thus supports the existence of an active import pathway for dTIS11.

### Role of the TZF in dTIS11 sub-cellular trafficking

**Figure 4:**
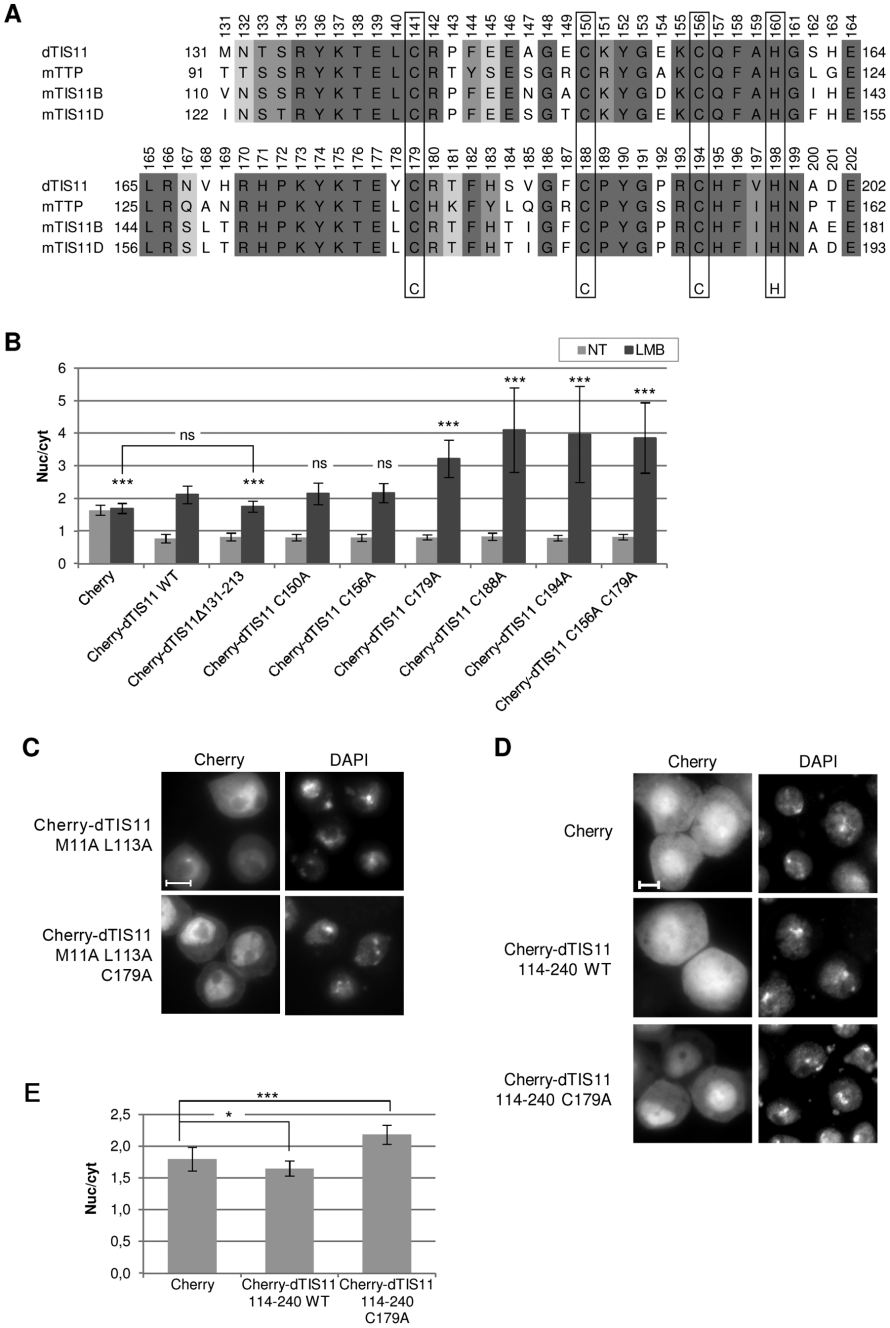
Role of the TZF in dTIS11 sub-cellular trafficking. (A) Alignment of the sequences of dTIS11, hTTP, hTIS11B and hTIS11D TZF domains. Conserved residues are shown in grey. Cysteine and histidine residues contributing to zinc chelation are framed. (B) Localization of dTIS11 mutants lacking the TZF domain or bearing point mutations substituting cysteines involved in dTIS11 RNA-binding activity into alanines. These mutants were expressed in fusion with Cherry in S2 cells that were treated or not with LMB (10 ng/ml, 5h). The subcellular distribution of the constructs was quantified for 30–40 cells as described in Fig. 2. Bars show the average of the nuc/cyt ratio ± s.d. Asterisks above the bars indicate the significance of the difference with Cherry-dTIS11 WT. ***: p<0.0001; ns: non-significant (p>0.05) (U-tests). The absence of significant difference between Cherry-dTIS11 WT nuc/cyt ratio and those of the ZnF1 mutants was observed in three independent experiments. The significant difference between the nuc/cyt ratio of Cherry-dTIS11 WT and those of the C188A and C194A mutants in LMB-treated cells was reproduced in two independent experiments. Ten independent experiments confirmed the significant difference between the nuc/cyt ratios of Cherry-dTIS11 and Cherry-dTIS11 C179A in LMB-treated S2 cells. (C) Localization of dTIS11 mutants bearing point mutations in the NES (M111A/L113A) alone or in combination with the C179A substitution in the ZnF2. Bar  =  5 µm. (D) Subcellular distribution of Cherry protein fused or not with WT or C179A versions of dTIS11 TZF in S2 cells. Bar  =  5 µm. (E) Quantification and statistical significance of the data shown in (D). The analysis was performed as in Fig.2. Bars show the average of the nuc/cyt ratio ± s.d. *: p<0.05; ***: p<0.0001 (U-tests). The significant difference between Cherry and Cherry-dTIS11 114-240 C179A nuc/cyt ratios was observed in two independent experiments.

Several studies [29]–[32] have established that the RNA-binding activity of TIS11 proteins is mediated by their conserved tandem zinc finger domain (TZF) (Figure 4A). We investigated the importance of dTIS11 TZF domain for its nucleo-cytoplasmic distribution. Plasmids encoding dTIS11 fused to Cherry or to the V5 tag with deletion of the whole TZF domain (Cherry-dTIS11 Δ131–213) or with point mutations at the Zn^2+^-coordinating cysteine residues (C150A, C156A, C179A, C188A, C194A and double mutant C156A/C179A) were generated and transfected into S2 cells. All these mutants proved to be unable to enhance the destabilization of an ARE-bearing reporter mRNA when overexpressed in S2 cells, whereas Cherry-dTIS11 efficiently decreased the half-life of the reporter mRNA as compared to Cherry alone (data not shown). While their ARE-binding ability seemed equally abolished, the TZF mutants displayed strikingly different abilities to relocate into the nucleus in response to LMB. The mutant lacking the two ZnFs (Cherry-dTIS11 Δ131–213) showed a slightly reduced nuclear accumulation compared to that of the WT in LMB-treated cells (Fig. 4B and S3A), which suggests that this domain could contribute to the nuclear import of dTIS11. The C150A and C156A mutants, in which the first zinc finger (ZnF1) is disrupted, were distributed similarly to the WT, whereas the C179A, C188A and C194A mutants, in which the second zinc finger (ZnF2) is disrupted, accumulated into the nucleus far more efficiently than the WT in response to LMB. The double mutant C156A/C179A showed the same localization patterns as the ZnF2 simple mutants (Fig. 4B and S3A,B,C). The effect of the C179A mutation on dTIS11 localization was also evaluated in absence of leptomycin B, by combining the C179A mutation with the double point mutation of the NES described above (M111A/L113A, Fig. S2E). Cherry-dTIS11 M111A L113A C179A was more nuclear than Cherry-dTIS11 M111A L113A (Figure 4C). This confirmed that disruption of the ZnF2 promotes the nuclear localization of dTIS11, independently of the presence of leptomycin B.

To further investigate the role of the TZF domain in the control of dTIS11 localization, we generated a Cherry fusion protein containing dTIS11 TZF (a.a. 114–240). The same construct with the C179A mutation was generated in order to test the ability of the TZF to mediate nuclear import in the absence of ZnF2 folding. As shown in Fig.4D and E, the mutated TZF conferred a marked nuclear localization to Cherry, which indicates that this sequence may act as an NLS. By contrast, the addition of the WT TZF did not significantly affect Cherry localization. Overall, our results are consistent with the hypothesis that the TZF of dTIS11 contains a cryptic NLS whose activity is impaired by ZnF2 ion coordination and folding.

### dTIS11 nuclear import is dependent on Transportin

**Figure 5:**
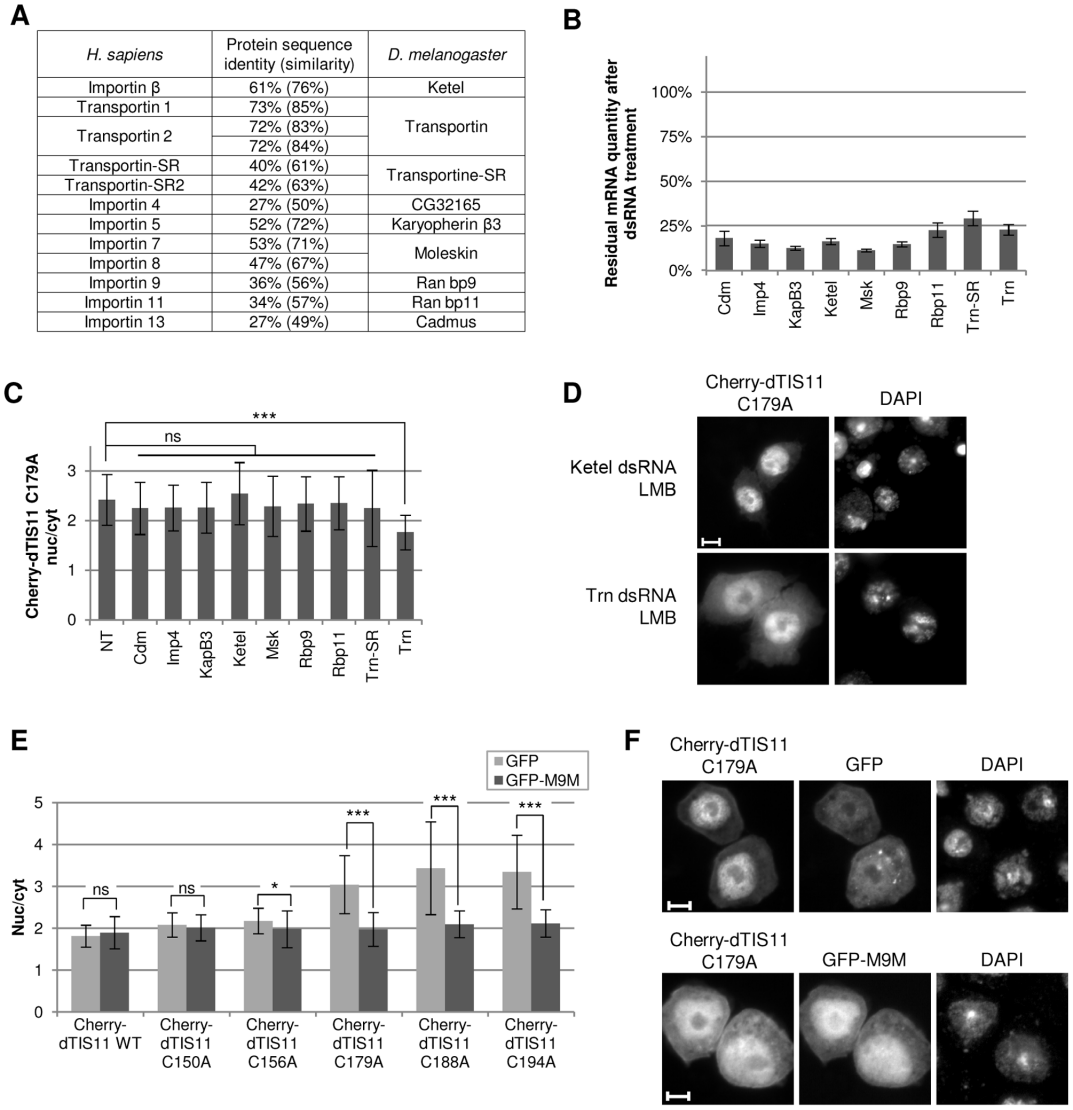
Screening of *Drosophila* importins to identify dTIS11 nuclear import machinery. (A) Human karyopherins and their *Drosophila* homologs. The identity/similarity of sequence is indicated. (B) Inhibition of *Drosophila* karyopherin expression by RNA interference. S2 cells were incubated for 4 days in the absence or in the presence of dsRNA targeting the indicated karyopherins. Cells were harvested and karyopherin mRNAs were quantified by qRT-PCR. The values are expressed relatively to untreated samples. The error bars represents the standard deviation over a triplicate measure. (C) Subcellular distribution of Cherry-dTIS11 C179A protein upon inhibition of karyopherin expression. S2 cells were treated with dsRNA targeting the indicated karyopherins as described in (B), transfected with Cherry-dTIS11 C179A construct and treated with LMB (10 ng/ml, 1h). Data acquisition and quantification were performed as in Fig. 2. Bars show the average of the nuc/cyt ratio ± s.d. ns: non-significant (p>0.05); ***: p<0.0001 (U-tests). (D) Representative images of the nucleo-cytoplasmic distribution of Cherry-dTIS11 C179A mutant in Ketel dsRNA- and Trn dsRNA-treated S2 cells in the presence of LMB. Bar  =  5 µm. Results shown in (B), (C) and (D) are representative of three independent experiments. (E) S2 cells were transfected to express wild-type or mutant versions of dTIS11 fused to Cherry, in combination with GFP alone or in fusion with the M9M peptide. Twenty hours post-transfection, cells were treated with LMB (10 ng/ml, 1h), fixed and observed by direct fluorescence microscopy. The subcellular distribution of the Cherry fusions was quantified as described in Fig. 2 for cells exhibiting GFP fluorescence. Bars show the average of the nuc/cyt ratio ± s.d. *: p<0.05; ***: p<0.0001; ns: non-significant (p>0.05) (t-tests). The absence of significant difference between Cherry-dTIS11 WT nuc/cyt ratios in GFP versus GFP-M9M-expressing cells and the significant difference between Cherry-dTIS11 C179A nuc/cyt ratios in these two conditions were observed in five independent experiments. (F) Representative images of the nucleo-cytoplasmic distribution of Cherry-dTIS11 C179A in S2 cells expressing GFP or GFP-M9M and treated with LMB as in (E). Bar  =  5 µm.

We next developed a screening assay to identify the karyopherin involved in dTIS11 import. Twelve importins are known in mammals. Sequence alignments recognized nine putative homologs in *Drosophila* [3] (Fig. 5A). An import function has been confirmed for three of them: Ketel, which is the Importinβ ortholog [33], Transportin-SR [34] and Moleskin [35], [36]. We produced double-stranded RNAs (dsRNAs) targeting each of these nine candidates to knock down their expression by RNA interference in S2 cells. Q-PCR experiments confirmed that the nine dsRNAs efficiently inhibited the accumulation of the targeted mRNAs (≥ 70% inhibition, see Fig. 5B). Because the ZnF2 point mutants showed a more efficient import than the WT, we first examined the localization of Cherry-dTIS11 C179A in our screening. Leptomycin B was added in order to block nuclear export. We observed a significant and reproducible diminution of Cherry-dTIS11 C179A nuclear/cytoplasmic ratio in cells where Trn expression was knocked down (Fig. 5C,D) compared to that observed in untreated cells. By contrast, the reduction of the expression of the eight other importins had no significant effect on Cherry-dTIS11 C179A nuclear accumulation (Fig. 5C). No variation was detected in the nuclear/cytoplasmic ratio of the dTIS11 WT fusion (data not shown), presumably due to Zn^2+^-mediated ZnF2 folding.

To confirm a role of *Drosophila* Transportin (dTRN) in dTIS11 nuclear import, we exploited the strong identity of mammalian and *Drosophila* Transportins within their NLS-binding domains and blocked dTRN activity by overexpressing the M9M peptide. This peptide was previously described as an inhibitor of the Transportin 1 pathway in mammals [37]. To validate this approach, we first verified that hnRNPA1, the best characterized cargo of mammalian Transportin 1 [38], is efficiently imported by dTRN into the nucleus of S2 cells. We observed that GFP-hnRNPA1 is exclusively nuclear in untreated S2 cells and that it partially relocalizes to the cytoplasm under dsRNA-mediated knock-down of dTRN expression (Fig. S4A). Secondly, we show that a major redistribution of GFP-hnRNPA1 into the cytoplasm occurs upon expression of Cherry-M9M (Fig. S4B), confirming the ability of the M9M peptide to block dTRN nuclear import activity in *Drosophila* cells.

We then analyzed the effect of M9M peptide expression in fusion with GFP on the LMB-driven nuclear accumulation of various Cherry-dTIS11 constructs. Cherry-dTIS11 WT localization was similar in LMB-treated cells expressing GFP or GFP-M9M. Fusions containing a point mutation in the ZnF1 (Cherry-dTIS11 C150A or C156A) accumulated into the nucleus to the same extent as the wild-type fusion, and this relocalization was not hindered by the expression of GFP-M9M (Cherry-dTIS11 C150A), or only very slightly (Cherry-dTIS11 C156A). By contrast, we observed a marked decrease in the nuclear relocalization of ZnF2 mutants (Cherry-dTIS11 C179A, C188A or C194A) upon GFP-M9M expression (Fig. 5E,F). All together, our results indicate that disrupting dTIS11 ZnF2 facilitates nuclear import through a Transportin-dependent import mechanism.

### dTIS11 import is mediated by a basic PY-NLS-like motif located in the TZF domain

Mammalian Transportin recognizes many cargoes via PY-NLSs. These are characterized by a C-terminal PY motif close behind a basic residue (R/K/H-X_2-5_-P-Y). The N-terminal part of the NLS can either be a hydrophobic motif fitting the loose Φ-G/A/S-Φ-Φ consensus (where Φ is a hydrophobic amino acid) or a patch enriched in basic residues. The linker separating the amino-terminal motif from the carboxy-terminal PY is 8-13 a.a. long and the whole sequence is structurally disordered [38].

**Figure 6:**
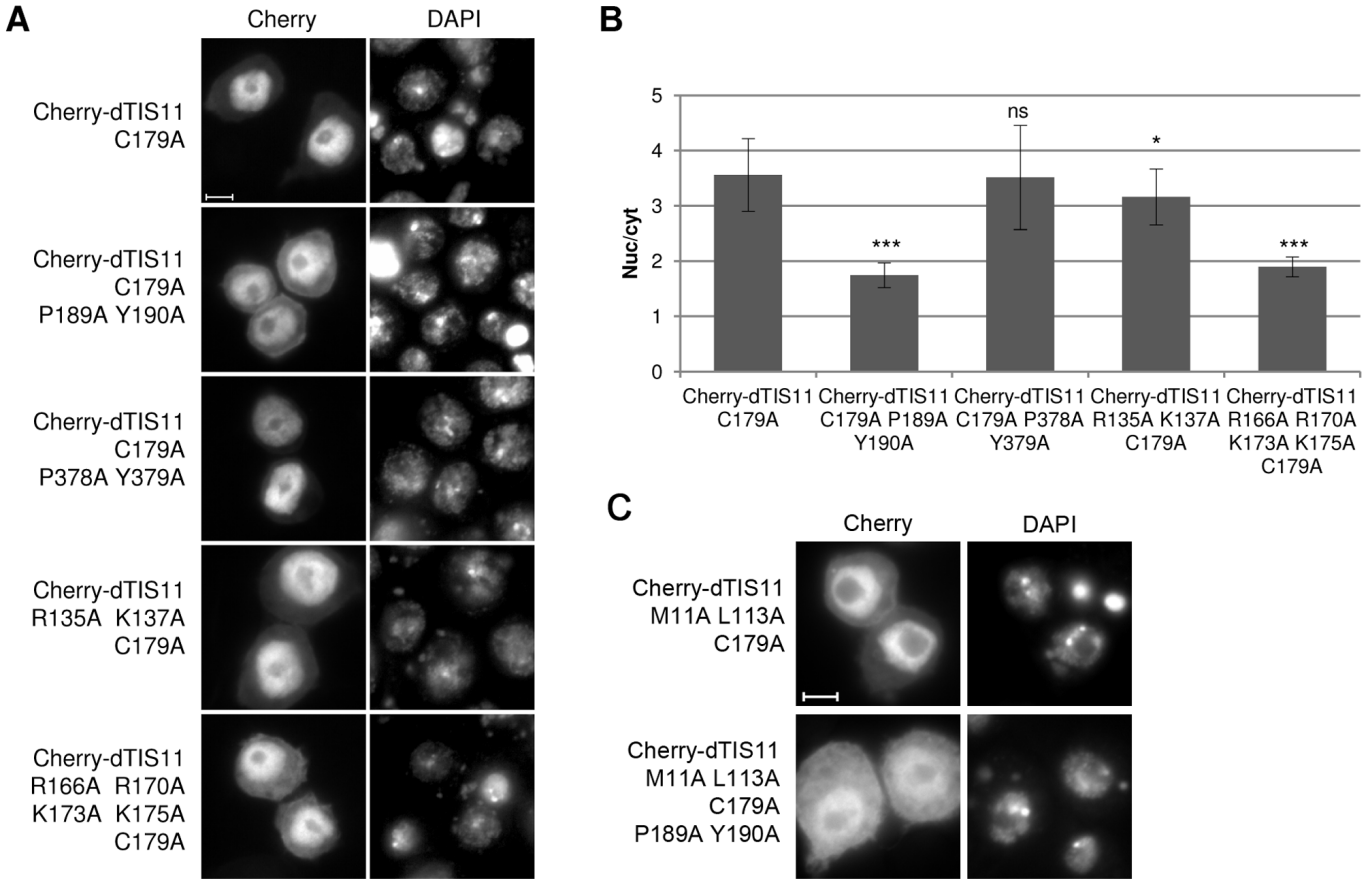
Identification of a cryptic PY-NLS-like motif in dTIS11 TZF. (A) Plasmids coding for Cherry fused to WT or mutant versions of dTIS11 were expressed in S2 cells treated with LMB (10 ng/ml, 5h) and the localization of the fusions was observed by fluorescence microscopy. Bar  =  5 µm. (B) The subcellular distribution of the fusions was quantified as described for Fig.2. Bars show the average of the nuc/cyt ratio ± s.d. *: p<0.05; ***: p<0.0001; ns: non-significant (U-tests). The significant differences between the nuc/cyt ratio of Cherry-dTIS11 C179A and those of the C179A/P189A/Y190A and R166A/R170A/K173A/K175A/C179A mutants were observed in two independent experiments. (C) Plasmids coding for Cherry fused to dTIS11 M111A L113A or dTIS11 M111A L113A C179A P189A Y190A were expressed in S2 cells and the localization of the fusions was observed by fluorescence microscopy. Bar  =  5 µm.

We noticed that a PY motif is present at positions 189-190 in dTIS11 ZnF2 and that the linker between the two ZnFs is enriched with basic residues. We thus generated and expressed dTIS11 mutants combining the C179A mutation with several substitutions in the suspected NLS region. We observed that mutation of the PY motif located at amino acids 189-190 drastically decreased dTIS11 C179A nuclear relocalization in response to LMB (P189A/Y190A, Fig. 6A,B). In contrast, mutation of the only other PY motif present in dTIS11 sequence (P378A/Y379A) had no effect. Substitution of R135 and K137 (which precede the ZnF1) with alanine very slightly diminished the LMB-driven relocalization, while substitution of the basic residues in the linker region and in the beginning of ZnF2 (R166A/R170A/K173A/K175A) had the same drastic effect as the P189A/Y190A mutations (Fig.6A,B). The P189A/Y190A mutations also conferred a more cytoplasmic localization to the Cherry-dTIS11 M111A L113A C179A mutant, which indicates that the function of the PY-NLS-like motif is independent of leptomycin B (Fig. 6C).

In conclusion, nuclear import of dTIS11 C179A appears to be mediated by a variant of the basic-type PY-NLS. It involves a C-terminal PY motif and several basic residues found 23 to 14 positions upstream from the PY motif in the primary structure. We also observed a minor contribution from two basic residues that lay more than 50 positions upstream from the PY motif (R135 and K137), but could be physically close because of the buckle structure of the ZnF1. The most original feature of this NLS is however its embedded position in the ZnF domain, whose folding competes with dTRN-mediated nuclear import.

### Conservation of the nuclear import of TIS11 proteins during evolution

**Figure 7:**
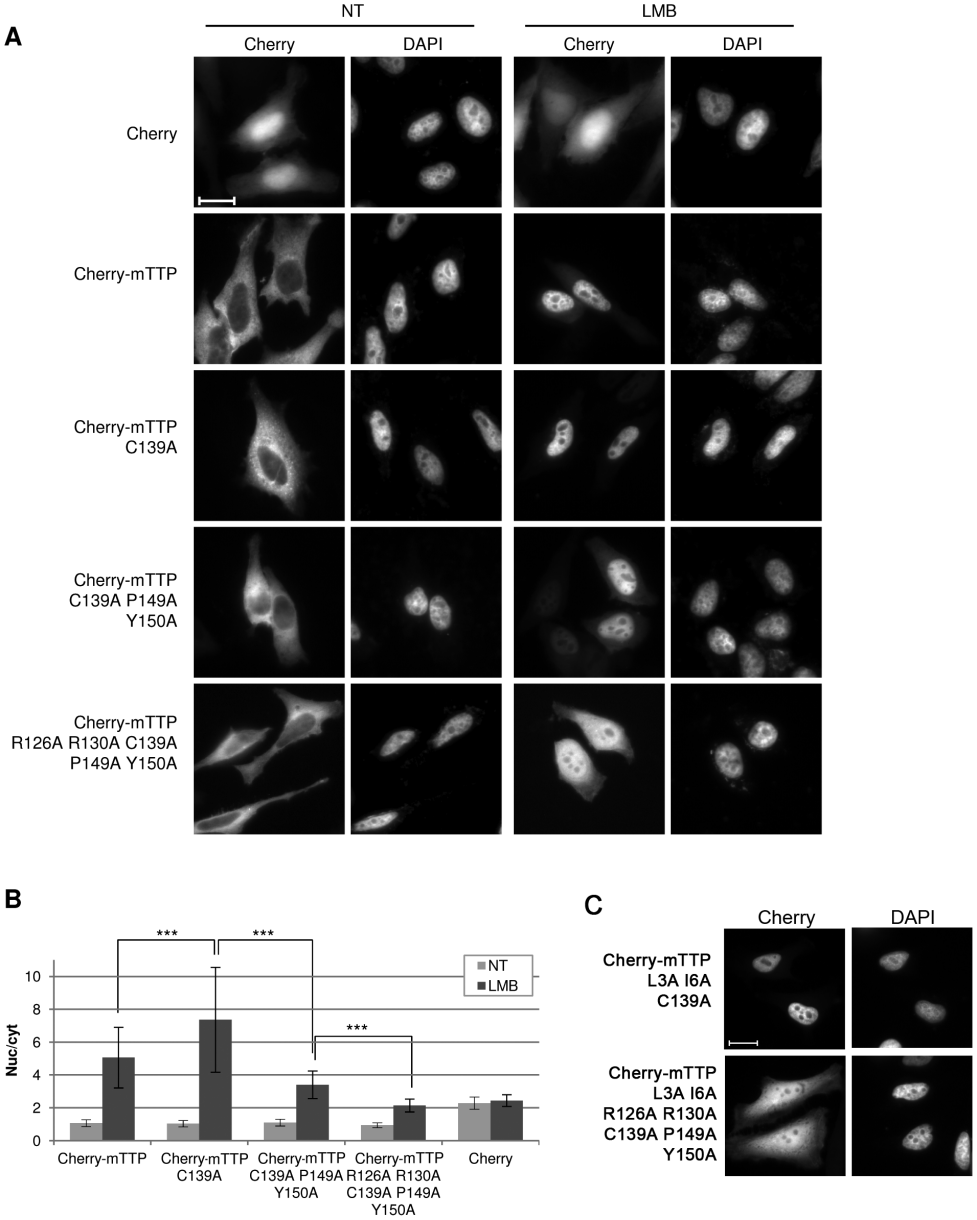
Influence of C139A, P149A/Y150A and additional mutations on the nucleo-cytoplasmic distribution of TTP. (A) HeLa cells were transfected to express Cherry or fusions of Cherry to wild-type or mutant versions of mTTP. Twenty-four hours later, cells were treated or not with LMB (10 ng/ml, 3h), fixed and observed by fluorescence microscopy. Bar  =  20 µm. (B) The subcellular distribution of Cherry fusions was quantified as described in Fig.2. Bars show the average of the nuc/cyt ratio ± s.d. ***: p<0.0001; ns: non-significant (t-tests). The significant difference shown between the nuc/cyt ratio of Cherry-mTTP WT and that of Cherry-mTTP C139A has been observed in 3 independent experiments. The significant difference between the nuc/cyt ratio of Cherry-mTTP C139A and that of Cherry-mTTP R126A R130A C139A P149A Y150A was observed in two independent experiments. (C) Localization of TTP mutants bearing mutations in the NES (L3A I6A) and in the ZnF2 (C139A) alone or in combination with mutations in the NLS (R126A R130A P149A Y150A). Bar  =  20 µm.

As shown in Fig. 4A, *Drosophila* and mammalian TIS11 proteins display a very high degree of conservation within the TZF domain (85% identity and 93% similarity between dTIS11 and TTP). We thus investigated whether the nuclear import of dTIS11 mammalian homologs such as murine TTP (mTTP) obeys the same rules as that of dTIS11. We first tested whether mTTP nuclear import is favored by ZnF2 unfolding. In this purpose, we compared the LMB-induced nuclear accumulation of a wild-type Cherry-mTTP fusion with a mutant in which a Zn^2+^-coordinating cysteine of ZnF2 is replaced by an alanine (Cherry-TTP C139A) in HeLa cells. As shown in Fig. 7, the mutant is markedly more nuclear than the wild-type protein upon LMB treatment, thereby indicating that the nuclear import of mTTP is increased upon ZnF2 destructuration. The effect of this mutation however is less pronounced than observed for *Drosophila* TIS11, which is probably due to a more efficient nuclear relocalization of the wild-type mammalian protein.

All the residues identified as part of the PY-NLS in dTIS11 are conserved in mammalian TIS proteins (Fig. 4A). Furthermore, in previous studies, point mutation of the two arginine residues located between the two ZnFs were reported to reduce the nuclear accumulation of TTP and of ZFP36L3 [20], [39], in accordance with what we observed for dTIS11. We thus examined whether point mutation of the key PY residues within TTP ZnF2 would affect the LMB-induced nuclear accumulation of the C139A mTTP mutant. We observed that mutation of the PY motif led to a significant decrease in the nuclear accumulation of the C139A mutant upon LMB treatment (Fig.7, compare Cherry-mTTP C139A with Cherry-mTTP C139A P149A Y150A). We then confirmed that additional mutation of the arginine residues R126 and R130 further reduced the nuclear level of the fusion protein to that of unfused Cherry (Fig.7B). The effect of the PY-NLS-like motif mutation was also evaluated in the context of a NES-mutated mTTP (L3A/I6A). We observed that the R126A/R130A/P149A/Y150A mutations strongly decreased the nuclear fraction of the Cherry-mTTP L3A I6A mutant (Fig. 7C). All together, these observations suggest that the nuclear import of mTTP is regulated by ZnF2 folding and that it is mediated by a basic PY-NLS-like motif located within the TZF.

## Discussion

This study aimed at characterizing the nucleo-cytoplasmic traffic of members of the TIS11 protein family. First, we identify a CRM1-dependent NES composed of amino acids 101 to 113 in *Drosophila* dTIS11 protein. This sequence complies with the proposed consensus NES for CRM1. Interestingly, mammalian TTP, TIS11B and TIS11D proteins are also exported by CRM1 but the NESs involved in CRM1 recruitment are different and are located in other parts of the proteins (in the N-terminal extremity for TTP, in the C-terminal extremity for TIS11B and TIS11D) [19]. It was recently reported that Cth2, a yeast protein displaying similarities to the TIS11 family [32], is exported from the nucleus in association with mRNAs through the Mex67 pathway rather than by CRM1 [40]. Although we do not exclude a similar mechanism for dTIS11 nuclear export, we clearly demonstrate that CRM1 inhibition massively relocalizes dTIS11 in the nucleus, thereby indicating that CRM1 constitutes the major nuclear export pathway of dTIS11, as it is for TTP, TIS11B and TIS11D. All together, these observations indicate that the export mechanism of TIS11 proteins significantly diverged during evolution.

In comparison with their nuclear export, the mechanisms governing the nuclear import of TIS11 proteins are poorly understood. Here, we show that dTIS11 nuclear accumulation is impaired upon ATP depletion, which supports the existence of an active import pathway for dTIS11. However, this process appears rather inefficient except when ZnF2 folding is disrupted by mutating one of the Zn^2+^-coordinating cysteines. Upon ZnF2 unfolding, dTIS11 nuclear import relies on Transportin, as LMB-driven relocalization of dTIS11 into the nucleus is markedly reduced upon inhibition of Transportin expression or activity. Moreover, we show that dTIS11 TZF bears the NLS activity and includes a motif similar to the PY-NLS consensus described for mammalian Transportin. We also show that these residues strongly condition the localization of a dTIS11 ZnF2 mutant.

The TZFs of mammalian TTP, TIS11B, ZFP36L3 and of yeast Cth2 have been described as necessary for the nuclear accumulation of these proteins [19], [20], [39], [40]. Furthermore, the P189-Y190 residues that we have identified as crucial for dTIS11 import are conserved in Cth2 and in the four mammalian TIS proteins. Recently, Transportin1 has been shown to interact with hTTP, mediating its migration in cytoplasmic granules under stress conditions [41]. These observations suggested that the Transportin-dependent import described here for dTIS11 could also operate for TIS11 proteins from yeast to mammals. Indeed, we show that substitution of a cysteine in TTP ZnF2 with alanine (C139A) results in an increased nuclear accumulation of a Cherry-TTP fusion upon LMB treatment in HeLa cells (Fig. 7), albeit to a lesser extent than observed for dTIS11 (Fig. 4B). Furthermore, we provide evidence that mutation of the PY-NLS-like motif in TTP TZF reduces the nuclear relocalization of the TTP C139A mutant upon LMB treatment. Our results thus suggest that the mechanism governing TIS11 protein nuclear import has been conserved during evolution.

Interestingly, the NLS we identified in dTIS11 and mTTP TZF only partially corresponds to the PY-NLS consensus. It involves a PY motif and a basic-enriched motif, as described for several Transportin cargoes such as hnRNP M, PQBP-1, YBP-1 and HEXIM1 [38], [42], but the linker separating the two elements is longer and the basic motif is more dispersed than in these proteins. Consequently, TIS11 proteins were not included in a list of predicted Transportin cargoes [38]. A recent report on the Transportin-mediated nuclear import of Huntingtin further documents the variability of the PY-NLSs [43]. All together, our and their data suggest that the current definition of PY-NLSs should be revisited to embrace the diversity of all the cargoes imported by this karyopherin.

Another remarkable feature of the NLS located in TIS11 protein TZF is that it seems to promote nuclear import only upon modification of ZnF2 Zn-coordination properties. While the structurating effect of Zn coordination on ZnF folding is extensively documented, it appears not always sufficient to mediate ZnF folding. Indeed, earlier structural analyses of the hTTP TZF domain by nuclear magnetic resonance revealed that the two ZnFs differ from each other in their folding characteristics. When a synthetic peptide corresponding to hTTP TZF is dissolved in a Zn^2+^-containing solution, only the residues from ZnF1 can be assigned, while those of the linker region and of the ZnF2 remain unassigned [44]. These observations lead us to hypothesize that TIS11 proteins could oscillate between two states: one in which the TZF is fully ordered, and one in which it is less ordered in its carboxy-terminal part. In the latter state, the PY-containing motif would be more accessible and could mediate the nuclear accumulation of the protein. Destructuration of ZnF2 by mutation of residues involved in Zn coordination would thus stabilize this second state, whereas unfolding of ZnF1 would not enhance exposition of the PY-containing motif.

The functional role of TIS11 protein nuclear import is unclear. At least three hypotheses can be considered. First, the import of TIS11 proteins into the nucleus might be a way to reduce their cytoplasmic pool, therefore limiting their activity in ARE-mediated decay. We attempted to test this hypothesis in our *Drosophila* model and observed that the ARE-containing mRNA encoding Cecropin A1 (CecA1) is markedly stabilized in a polyadenylated form upon CRM1 inhibition by LMB (Fig.S5), which is reminiscent of the effect of dTIS11 knockdown on this mRNA [16]. However, further experiments aiming at determining whether the destabilizing activity of dTIS11 could be affected by deletion or mutation of the NES were inconclusive. We hypothesize that overexpression of a NES-mutated dTIS11 protein still leads to sufficient dTIS11 cytoplasmic accumulation to promote AMD.

Alternatively, nuclear import might allow the association of TIS11 proteins with their RNA targets in the nucleus, from where they could direct them to the cytoplasm, acting as an adaptor for CRM1-dependent export of specific mRNAs. Nuclear export of the c-fos mRNA, which bears an ARE in its 3’UTR, was shown to be inhibited by LMB in HeLa cells [45]. This suggested a potential involvement of CRM1 in the export of ARE-containing mRNAs, possibly mediated through adapter RBPs. Preliminary results from fluorescent in situ hybridization experiments on CecA1 mRNA in S2 cells do however not support the hypothesis of a dTIS11-mediated, CRM1-dependent export of CecA1 mRNA as this messenger appeared cytoplasmic regardless of a LMB treatment (data not shown).

A third possibility is that TIS11 proteins might have a nuclear function distinct from their involvement in ARE-mediated decay. In yeast, Cth2 has been shown to modulate the selection of the poly(A) site of several mRNAs [46]. More recently, studies have shown that TIS11B regulates the 3’ end processing of the *Dll4* mRNA [47], and that TTP could play a role in the polyadenylation of ARE-containing mRNAs via its interaction with poly(A)-binding protein nuclear 1 [48]. It will be of interest to determine whether dTIS11 also participates in nuclear processes and to establish how the Transportin/TIS11 interaction is modulated to intervene in the various roles of TIS11 proteins.

## Materials and Methods

### Ethics statement

Animal care and experimental procedures were performed in accordance with the Belgian law of August 14^th^, 1986, as well as the royal decree of April 6^th^, 2010 on the protection of laboratory animals, under the laboratory licence 1500474, granted by the Service public fédéral de santé publique, sécurité de la chaîne alimenaire et environnement (Direction générale Animaux, Végétaux et Alimentation). The experimental procedure was approved by the Comité d’éthique du bien-être animal de l’Institut de Biologie et de Médecine Moléculaires de l’ULB (#CEBEA-05). Mice were checked daily and sacrificed by cervical dislocation.

### Plasmid construction

To construct the pMT-Cherry-mcs plasmid, the sequence encoding mCherry was amplified by PCR using the pmCherry-N1 (Clontech) as a template and oligonucleotides 5’aattctagagccaccatggtgagcaagggcgagga (for) and 5’aattgaattcttgtacagctcgtccatgc (rev). It was then inserted XbaI/EcoRI into the pMT/V5-His C plasmid (Invitrogen). Wild-type dTIS11 coding sequence was originally amplified from S2 cell cDNA and corresponded to GenBank entry AAA62666. To generate the pMT-Cherry-dTIS11 constructs, fragments encoding wild-type, deleted or point-mutated versions of dTIS11 were amplified by PCR and cloned EcoRI/XhoI or EcoRI/EcoRI into pMT-Cherry-mcs. Wild-type or mutant versions of dTIS11 were cloned EcoRI/XhoI in frame into the PMT/V5-His A (Invitrogen) to generate the pMT-dTIS11-V5 constructs. A FLAG tag was added at the N or C-terminus of dTIS11 by PCR and the resulting sequence was cloned EcoRI/XhoI or EcoRI/EcoRI into pMT-V5-His plasmids to generate the pMT-FLAG-dTIS11 and pMT-dTIS11-FLAG constructs, respectively. pMT-Cherry was constructed by inserting a dimer of oligonucleotides encoding a stop codon into the EcoRI site of pMT-mCherry-mcs. PMT-TIS11-Cherry was generated by pasting the sequence encoding mCherry from pmCherry-N1 into the EcoRI site of pMT/V5-His A (Invitrogen) and then adding the CDS of dTIS11 into the BamHI site of the construct. pMT-GFP-dTIS11 and pMT-LUC-ARE were described previously [18]. A pMT-GFP vector was created by inserting the eGFP sequence (amplified from peGFP-C3, Clontech, with the appropriate flanking restriction sites) between the XbaI and EcoRI sites of pMT/V5-His C. pMT-GFP-hnRNPA1 was generated by inserting mammalian hnRNPA1 sequence (320 a.a. isoform) into the BamHI site of pMT-GFP. The pMT-GFP-M9M was constructed by inserting the NcoI/BamHI GFP-peptide cassette from peGFP-M9M [49] into pMT-GFP. pcDNA-Cherry was generated by inserting the Cherry sequence from pmCherry-N1 XbaI/EcoRI into the pcDNA 3.1(-) (Invitrogen). The sequence of murine wild-type or point-mutated TTP was added into the EcoRI site of this vector to generate pcDNA-Cherry-mTTP constructs. Plasmids used to generate double-stranded RNAs for RNAi experiments were obtained by RT-PCR amplification of ∼500 bp fragments within the coding region of *Drosophila* importin mRNAs and cloning into the EcoRV site of the pBluescript II SK+ vector. All the DNA constructs were sequenced and maps are available on request. Enzymes were purchased from Invitrogen and Roche. Oligonucleotides were purchased from Sigma-Aldrich.

### Cell culture, treatment and transfection

*Drosophila* S2 cells (Invitrogen) were maintained in serum-free Express Five medium (Invitrogen) at 25.8°C. For fluorescence microscopy experiments, ∼10^6^ S2 cells were seeded on coverslips in 12-well plates and transfected the day before fixation. For Northern and Western blot experiments, 1.5×10^6^ S2 cells were seeded and transfected in 12-well plates the day before treatments and protein or RNA extraction. Transfection was performed with Fugene HD (Roche) according to the manufacturer’s instructions. Transcription driven by the metallothionein promoter was induced by adding 0.5 mM CuSO_4_ overnight. For mRNA half-life measurements, transcription was blocked with actinomycin D (5 µg/ml; Sigma-Aldrich) and the cells were harvested at the indicated time points. For induction of the *cecA1* gene transcription, peptidoglycan (Invivogen) was added to the culture medium at 7 µg/ml. dsRNA treatment were performed as described previously [16]. Hela cells were maintained in DMEM high glucose GlutaMAX (Invitrogen) supplemented with 10% fetal bovine serum (FBS, Gibco-BRL), sodium pyruvate and penicillin/streptomycin (Invitrogen). For fluorescence experiments, ∼10^5^ HeLa cells were seeded on coverslips in 12-well plates the day before transfection, which was performed with Lipofectamine 2000 (Invitrogen) according to the manufacturer’s instructions. Treatments and fixation were performed on the following day. Leptomycin B (LC Laboratories, Woburn, MA USA) was used at 10 ng/ml for the indicated time.

### Generation of dTIS11 monoclonal antibody

*Drosophila* TIS11 protein was expressed in *E. coli* as a fusion protein with a 6xHis tag at its N-terminus (pscodon vector, Delphi Genetics) and purified on a HisPur Cobalt Resin (Thermo scientific) in denaturing conditions (8 M urea) according to the manufacturer’s instructions. The purified protein was then dialyzed against a non-denaturing buffer containing acetic acid (20 mM). 20 µg of the purified protein was injected in BALB/c (Harlan) mice three times, with 2 weeks between each injection. Single spleen cells from immunized mice were fused with SP20 myeloma cells (American Type Culture Collection). Three successive selection steps were performed with ELISA verification to obtain a clone producing an anti-TIS11 antibody validated for western blot.

### Immunoblotting

S2 cells were lysed in EBC buffer [50 mM Tris (pH 8.0), 150 mM NaCl, 0.5% NP-40] containing complete EDTA-free protease inhibitor cocktail (Roche Diagnostics). 40 µg total proteins was denaturated at 95°C in Laemmli buffer and loaded on a 10% polyacrylamide gel. Separated proteins were transferred on a nitrocellulose membrane. After blocking in TBST containing 1.5% nonfat dry milk proteins (Bio-Rad), the membrane was incubated with the anti-dTIS11 antibody diluted 1/500 in blocking solution overnight at 4°C. Bands were visualized using an anti-murine HRP-conjugated IgG from sheep (GE Healthcare) and the SuperSignal West Pico chemiluminescent substrate (Thermo Scientific).

### Fluorescence microscopy

For observation of fluorescent protein fusions, coverslips were rinsed twice with PBS, fixed with 4% formaldehyde (prepared from paraformaldehyde dilution in PBS) for 10 min., washed three times with PBS for 10 min, rinsed with water, briefly air-dried, mounted on slides in 10 µl of DAKO fluorescent mounting medium (DAKO, Glostrup, Denmark) supplemented with 100 pg/ml 4’,6’-diamidino-2-phenylindole (DAPI) and sealed with nail polish. For immunofluorescence experiments, the cells were permeabilized with 0.5% Triton X-100 (Sigma-Aldrich) for 10 min. after fixation and rinsing. They were further rinsed, blocked in 5% BSA (Sigma-Aldrich) for 45 min. at RT and incubated overnight at 4°C with primary antibody diluted in 0.1% Tween 20 (Sigma-Aldrich) + 0.5% BSA (anti-FLAG 1/1000; anti-V5 1/1000; anti-dTIS11 1/500). They were then washed three times for 10 min. in 0.1% Tween 20 and incubated with an AlexaFluor 594 donkey anti-mouse IgG (Invitrogen) diluted 1/2500 or 1/5000 in PBS + 0.1% Tween 20 + 0.5% BSA. After three 10 min. washes in 0.1% Tween 20, coverslips were rinsed with water, dried and mounted as described supra. All dilutions of chemical reagents were initially performed in PBS.

### Image acquisition and analysis

Slides were observed with an Axio Observer Z.1 (Zeiss, Jena, Germany). A grid of 16–25 regularly spaced positions was defined using the Mark & Find module of the Zeiss AxioVision software and these positions were screened. When a position in the grid included healthy transfected cells, an image was acquired; otherwise, the operator moved to the nearest field of view fulfilling this criterion to acquire an image. TTP fusions tended to form cytoplasmic aggregates when the overexpression was high; only cells devoid of aggregates were considered in experiments that evaluate TTP localization. This systematic acquisition process was repeated for all the positions on the grid. Post-acquisition treatment and extraction of data from the images were performed with the Zeiss AxioVision software. Contrast and luminosity were adjusted faithfully to the initial images. The gamma was always kept to 1, except for DAPI images. For quantification experiments, the average fluorescence was measured in the raw.zvi images for the whole nucleoplasmic (nuc) and cytoplasmic (cyt) compartments, which were manually delimited. The localization of the imaged protein was then expressed as the nuc/cyt ratio. For each experimental condition, a series of 30–40 nuc/cyt ratios was compiled. We checked that this sampling was sufficient to get a good estimation of the means, i.e. characterized by low standard error of mean values (SEM usually ranging from 1.5% to 5% of the mean value, and always <8%). The normal form of the nuc/cyt ratio distribution was assessed with the Kolmogorov-Smirnov test. When all the series of an experiment passed the test, they were compared with 2-tailed unpaired t-tests, including Welch’s correction when the variances were detected as significantly different (using F-tests). When at least one of the series failed the normality test, all the comparisons inside the experiment were done with the non-parametric 2-tailed Mann-Whitney test. The number of replicate experiments performed is specified in the figure legends. The raw data of all experiments presented are given as supporting information (File S1).

### RNA analysis

Total RNA was purified using Tri reagent (MBI) according to the manufacturer’s instructions. Northern blot analyses were performed as described previously [16]. For qRT-PCR experiments, 1 µg of total RNA was treated with DNAseI (Invitrogen) and half of it was reverse-transcribed with an oligo(dT)20 using M-MLV reverse transcriptase (Sigma) according to the manufacturer’s instructions; the other half was mock-transcribed (without polymerase). QPCR reactions were performed with the qPCR MasterMixPlus w/o UNG for SYBR® Assay ROX (EuroGentec) in a StepOnePlus™ instrument (Applied BioSystems) and quantification was performed using the comparative C_q_ method. The C_q_ for negative controls (no template and mock-reverse transcribed samples) were always higher than the ones for the positive samples by at least 5. The reference gene used was Rp49. For each condition and primer pair, the C_q_ measure was done in triplicate with the equivalent of 25 ng of total RNA. At least two primer pairs were tested for each gene and the ones with the optimal melting curves were selected for further use. Primer were designed with Primer 3 [50] and their sequences are available upon request.

## Supporting Information

Figure S1 **Cherry-dTIS11 efficiently destabilizes a reporter mRNA containing an ARE in the 3’UTR.** S2 cells were transfected with pMT-Cherry or pMT-Cherry-dTis11 expression vectors and a PMT- Luc-ARE reporter gene, induced with CuSO_4_ overnight, and treated with actinomycin D for the indicated time before harvest of the cells and RNA extraction. Northern blots were hybridized with luciferase and rp49 antisense riboprobes. The luc/rp49 ratio was quantified with PhosphorImager and normalized on the t0 value. The normalized ratios were plotted on a semi-logarithmic graph, and mRNA half-lives were determined by exponential regression. Here are shown the average values for four independent experiments. Error bars represent s.d. for each time point. Mean values for luc-ARE mRNA half-life ± s.d. are given for Cherry and Cherry-dTIS11 expressing cells.
(TIF)

Figure S2 **Hydrophobic residues are necessary for dTIS11 101-113 NES activity.** (A) Localization of various deletion mutants of dTIS11 fused to the V5 epitope in S2 cells. Cells were treated (right) or not (left) with LMB (10 ng/ml for 5 h) before observation by immunofluorescence microscopy. Bar  =  5 µm. (B) Quantification of the relocalization of the various mutants in response to LMB from the experiment presented in (A). For each condition, the nuc/cyt ratio was measured in 30-40 cells as described in Fig. 2. Bars show the average of the nuc/cyt ratio ± s.d. *: p<0.05; ***: p<0.0001 (U-tests); ns: non-significant. (C) dTIS11 101-113 NES sequence. Hydrophobic residues are in bold. (D) Localization of various NES point-mutation or deletion mutants of dTIS11 fused to Cherry in S2 cells treated (right) or not (left) with LMB (10 ng/ml for 5 h). Bar  =  5 µm. (E) Quantification of the relocalization of the various mutants in response to LMB for the experiment presented in (D). The quantification was performed as in (B). Bars show the average of the nuc/cyt ratio ± standard deviation. ***: p<0.0001 (U-tests).
(TIF)

Figure S3 **Localization of dTIS11 mutants lacking the TZF domain or bearing point mutations altering Zn coordination of ZnFs**. (A) Representative images illustrating the localization of the mutants described in Fig. 4B. Bar  =  5 µm. (B) Representative images illustrating the localization of the wild-type, C179A or C156A C179A dTIS11 proteins fused to the V5 tag. Bar  =  5 µm. (C) Quantification of the relocalization of the various mutants in response to LMB in the experiment presented in (B). For each condition, the nuc/cyt ratio was measured in 30-40 cells as described in Fig. 2. Bars show the average of the nuc/cyt ratio ± s.d. ***: p<0.0001 (U-tests).
(TIF)

Figure S4 **Validation of the M9M peptide as an inhibitor of the Transportin pathway in S2 cells.** (A) Subcellular distribution of GFP fused to mammalian hnRNPA1 in S2 cells treated or not with dsRNA targeting Trn. The dsRNA treatment was performed as described in Fig. 5C. (B) Representative images illustrating the subcellular distribution of GFP-hnRNPA1 upon expression of Cherry alone or in fusion with the M9M peptide in S2 cells.
(TIF)

Figure S5 **Stabilization of CecA1 mRNA upon CRM1 inhibition by LMB in S2 cells.** (A) Schematic representation of the experimental setting. CecA1 mRNA accumulation was induced by stimulation of S2 cell with peptidoglycan for 3 hours after incubation of the cells in the presence or the absence of LMB (2h). Actinomycin D was then added to the cultures for the indicated time before harvest of the cells and RNA extraction. (B) Northern blots were hybridized with cecropinA1 and rp49 antisense riboprobes. (C) The cecA1/rp49 ratio was quantified with PhosphorImager and normalized on the t0 value. The normalized ratios were plotted on a semilogarithmic graph, and mRNA half-lives were determined by exponential regression. Average values for three independent experiments are shown. Error bars represent s.d. for each time point. Mean values for CecA1 mRNA half-life ± s.d. are given for cells treated or not with LMB.
(TIF)

File S1 **Raw data and statistical analyses for the quantitative fluorescence microscopy experiments.**
(PDF)
