# Supplementary figures and images for: A Masked PY-NLS in Drosophila TIS11 and Its Mammalian Homolog Tristetraprolin
Source: PLoS One. 2013 Aug 9;8(8):e71686. doi: 10.1371/journal.pone.0071686 (PMC3739726; doi:10.1371/journal.pone.0071686)

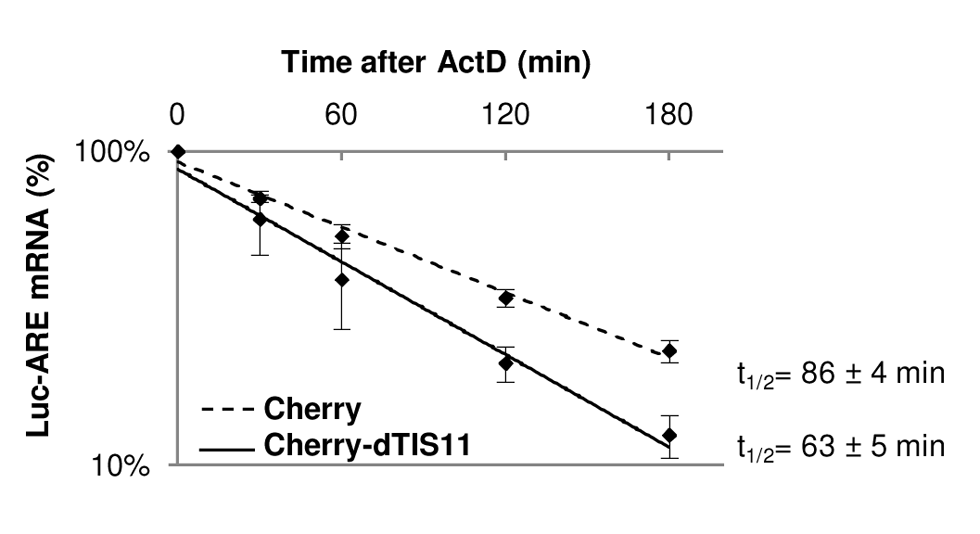

Supplement: Figure S1 — Cherry-dTIS11 efficiently destabilizes a reporter mRNA containing an ARE in the 3’UTR. S2 cells were transfected with pMT-Cherry or pMT-Cherry-dTis11 expression vectors and a PMT- Luc-ARE reporter gene, induced with CuSO4 overnight, and treated with actinomycin D for the indicated time before harvest of the cells and RNA extraction. Northern blots were hybridized with luciferase and rp49 antisense riboprobes. The luc/rp49 ratio was quantified with PhosphorImager and normalized on the t0 value. The normalized ratios were plotted on a semi-logarithmic graph, and mRNA half-lives were determined by exponential regression. Here are shown the average values for four independent experiments. Error bars represent s.d. for each time point. Mean values for luc-ARE mRNA half-life ± s.d. are given for Cherry and Cherry-dTIS11 expressing cells. (TIF) [file pone.0071686.s001.tif]

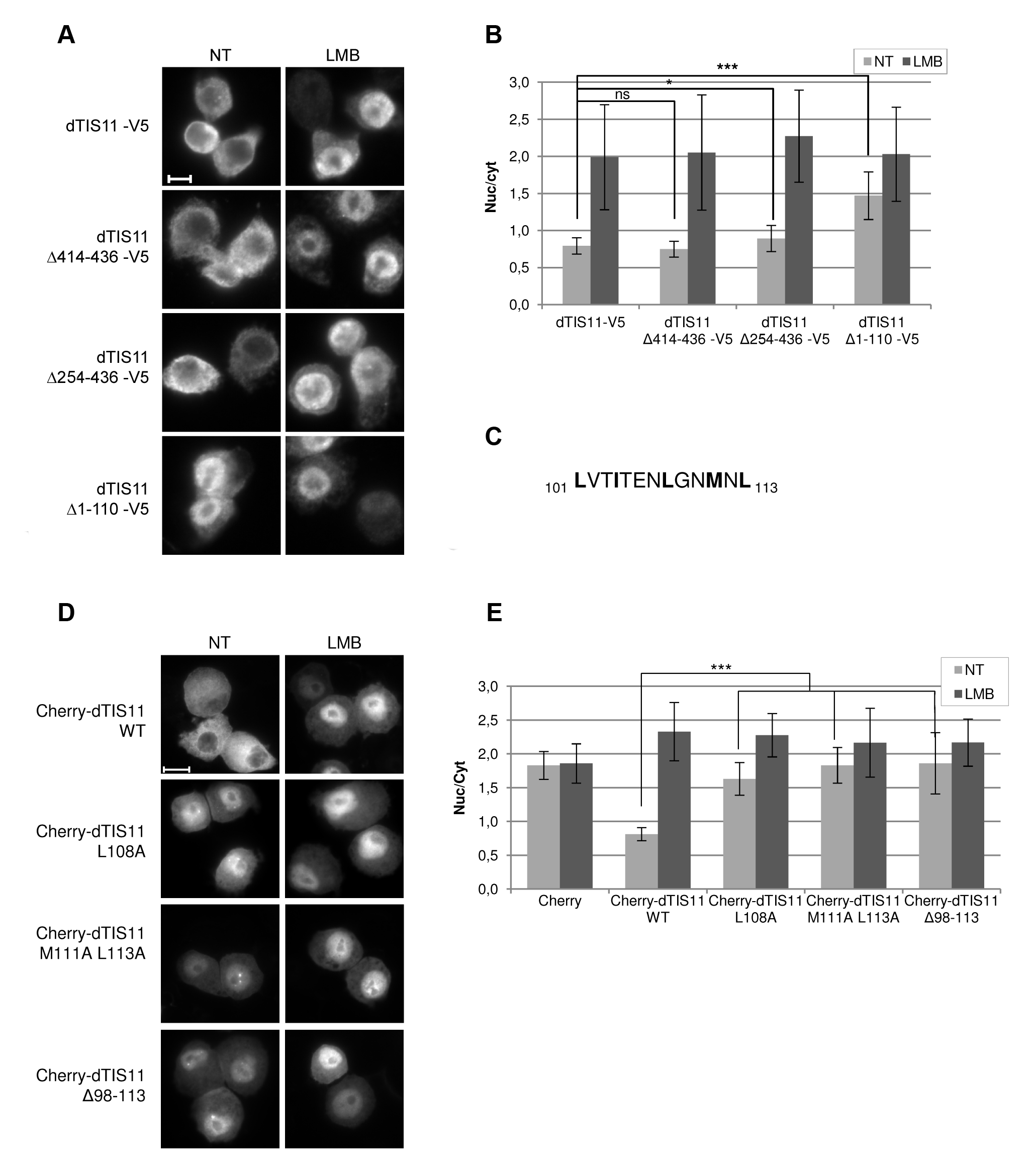

Supplement: Figure S2 — Hydrophobic residues are necessary for dTIS11 101-113 NES activity. (A) Localization of various deletion mutants of dTIS11 fused to the V5 epitope in S2 cells. Cells were treated (right) or not (left) with LMB (10 ng/ml for 5 h) before observation by immunofluorescence microscopy. Bar = 5 µm. (B) Quantification of the relocalization of the various mutants in response to LMB from the experiment presented in (A). For each condition, the nuc/cyt ratio was measured in 30-40 cells as described in Fig. 2. Bars show the average of the nuc/cyt ratio ± s.d. *: p<0.05; ***: p<0.0001 (U-tests); ns: non-significant. (C) dTIS11 101-113 NES sequence. Hydrophobic residues are in bold. (D) Localization of various NES point-mutation or deletion mutants of dTIS11 fused to Cherry in S2 cells treated (right) or not (left) with LMB (10 ng/ml for 5 h). Bar = 5 µm. (E) Quantification of the relocalization of the various mutants in response to LMB for the experiment presented in (D). The quantification was performed as in (B). Bars show the average of the nuc/cyt ratio ± standard deviation. ***: p<0.0001 (U-tests). (TIF) [file pone.0071686.s002.tif]

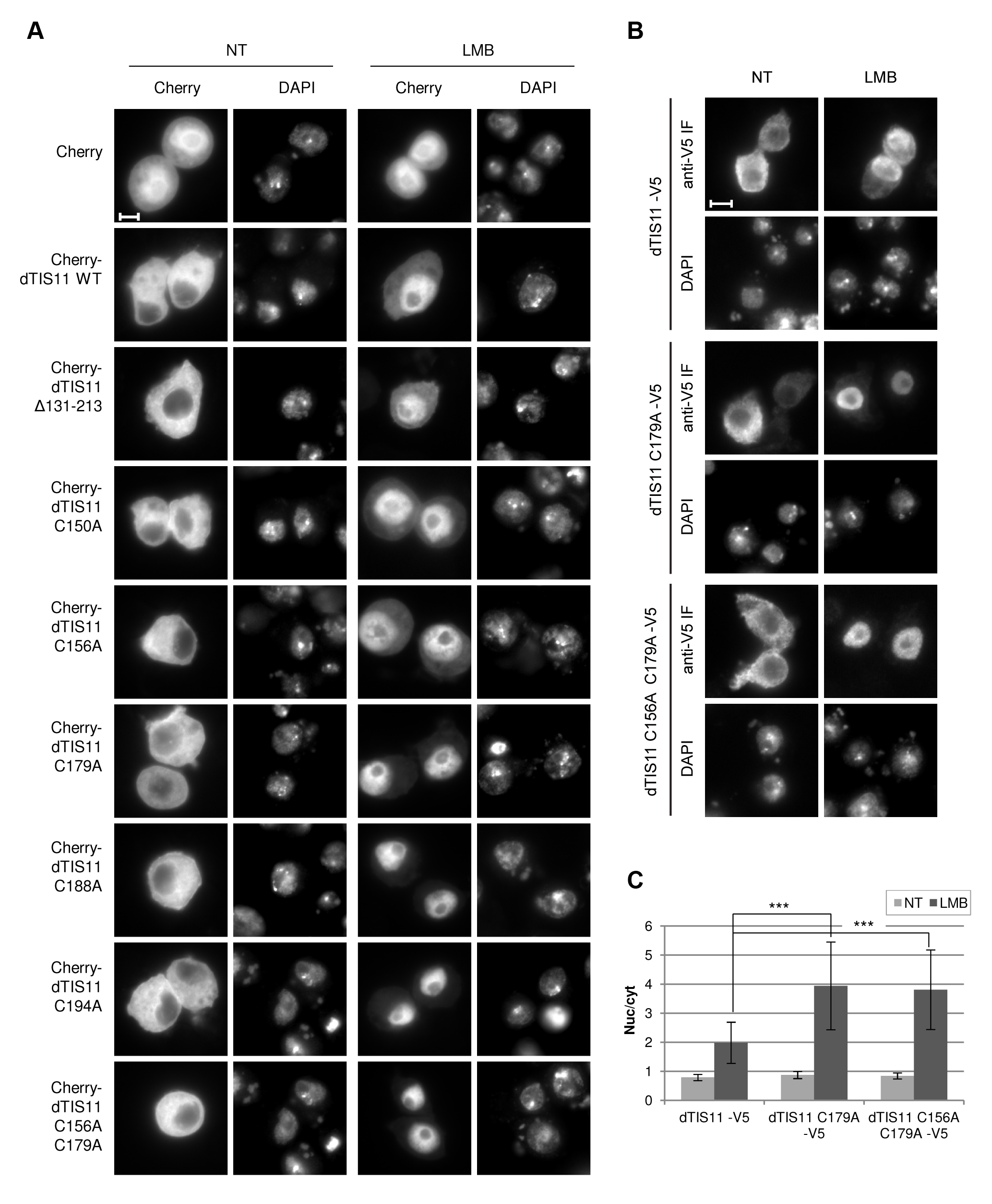

Supplement: Figure S3 — Localization of dTIS11 mutants lacking the TZF domain or bearing point mutations altering Zn coordination of ZnFs. (A) Representative images illustrating the localization of the mutants described in Fig. 4B. Bar = 5 µm. (B) Representative images illustrating the localization of the wild-type, C179A or C156A C179A dTIS11 proteins fused to the V5 tag. Bar = 5 µm. (C) Quantification of the relocalization of the various mutants in response to LMB in the experiment presented in (B). For each condition, the nuc/cyt ratio was measured in 30-40 cells as described in Fig. 2. Bars show the average of the nuc/cyt ratio ± s.d. ***: p<0.0001 (U-tests). (TIF) [file pone.0071686.s003.tif]

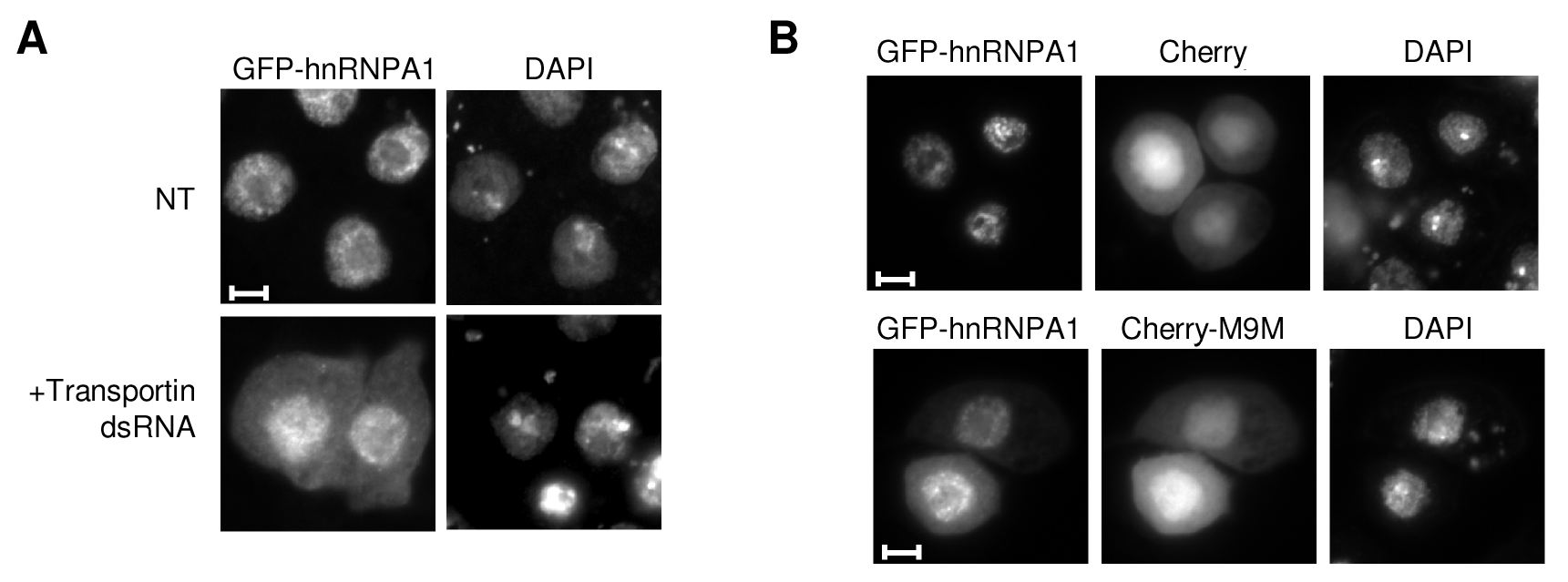

Supplement: Figure S4 — Validation of the M9M peptide as an inhibitor of the Transportin pathway in S2 cells. (A) Subcellular distribution of GFP fused to mammalian hnRNPA1 in S2 cells treated or not with dsRNA targeting Trn. The dsRNA treatment was performed as described in Fig. 5C. (B) Representative images illustrating the subcellular distribution of GFP-hnRNPA1 upon expression of Cherry alone or in fusion with the M9M peptide in S2 cells. (TIF) [file pone.0071686.s004.tif]

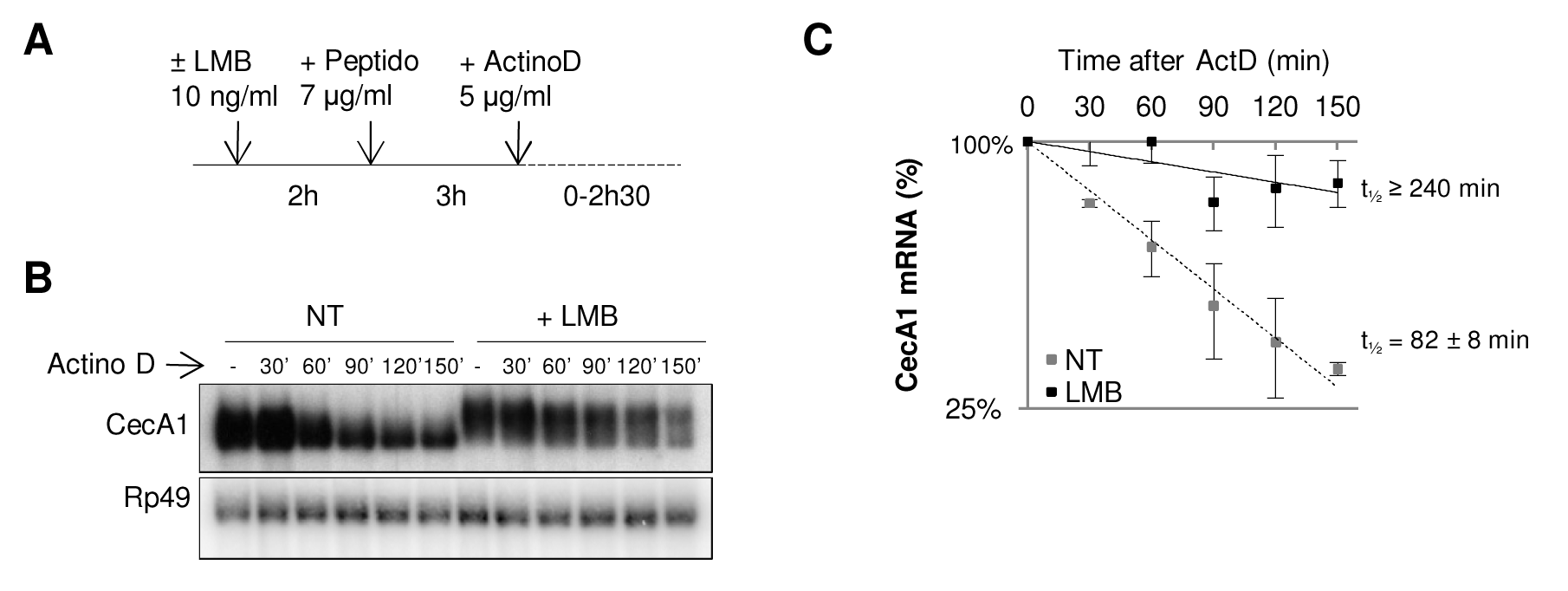

Supplement: Figure S5 — Stabilization of CecA1 mRNA upon CRM1 inhibition by LMB in S2 cells. (A) Schematic representation of the experimental setting. CecA1 mRNA accumulation was induced by stimulation of S2 cell with peptidoglycan for 3 hours after incubation of the cells in the presence or the absence of LMB (2h). Actinomycin D was then added to the cultures for the indicated time before harvest of the cells and RNA extraction. (B) Northern blots were hybridized with cecropinA1 and rp49 antisense riboprobes. (C) The cecA1/rp49 ratio was quantified with PhosphorImager and normalized on the t0 value. The normalized ratios were plotted on a semilogarithmic graph, and mRNA half-lives were determined by exponential regression. Average values for three independent experiments are shown. Error bars represent s.d. for each time point. Mean values for CecA1 mRNA half-life ± s.d. are given for cells treated or not with LMB. (TIF) [file pone.0071686.s005.tif]
