# Supplementary material for: A Masked PY-NLS in Drosophila TIS11 and Its Mammalian Homolog Tristetraprolin
Source: PLoS One. 2013 Aug 9;8(8):e71686. doi: 10.1371/journal.pone.0071686 (PMC3739726; doi:10.1371/journal.pone.0071686)
Supplement: File S1 — Raw data and statistical analyses for the quantitative fluorescence microscopy experiments. (PDF) [file pone.0071686.s006.pdf]

Figure 2C - Raw data

This file can be opened by GraphPad Prism (version 5.00 or later).

This file contains 1 data tables and 0 info tables:

- **Fig 2C new**

Fig 2C new

Cherry NT	Cherry LMB	Cherry-dTIS11 NT	Cherry-dTIS11 LMB	Cherry-dTIS11 delta415-436 NT	Cherry-dTIS11 delta415-436 LMB	Cherry-dTIS11 delta214-436 NT	Cherry-dTIS11 delta214-436 LMB	Cherry-dTIS11 delta98-113 NT	Cherry-dTIS11 delta98-113 LMB
1,63	2,03	0,75	2,51	0,66	2,1	0,66	2,51	1,43	1,73
1,6	1,44	0,85	2,01	0,99	1,94	0,63	1,48	1,7	2,54
1,37	1,51	0,93	2,29	0,89	2,13	0,66	1,75	1,35	2,95
1,58	1,46	0,85	2,4	0,9	2,1	0,7	1,59	1,51	1,41
1,89	2,25	0,75	2,06	0,84	1,77	0,75	2,41	1,05	2
1,76	1,69	0,8	1,76	0,93	1,55	0,69	2,18	1,36	2,28
1,4	1,49	0,8	2,41	0,57	1,93	0,74	2,55	1,7	1,88
1,39	1,42	0,6	2,15	0,62	2,21	1,01	2,98	1,54	2,11
1,43	1,33	0,98	2,01	0,81	1,73	0,75	1,88	1,02	1,85
1,61	1,99	0,89	2,07	0,75	1,53	0,83	1,96	1,52	2,01
1,76	1,83	0,68	2,16	0,96	2,17	0,8	2,18	1,64	2,82
1,86	1,63	0,85	1,97	0,78	2,02	0,66	2,23	1,69	1,89
1,8	2	0,79	1,96	0,82	2,21	0,8	1,86	1,26	1,91
1,7	1,94	0,88	2,42	0,69	2,09	0,94	2,45	1,36	2,16
1,55	1,88	0,74	1,95	0,75	1,86	0,89	3,11	1,62	2,05
1,25	1,66	0,69	2,08	0,81	1,61	0,66	2,13	1,13	2,11
1,46	1,78	0,71	2,09	0,7	2,5	0,84	2,07	1,47	2,08
1,52	1,88	0,77	2,26	0,5	2,5	0,77	1,98	1,62	1,58
1,46	1,91	0,87	2,58	0,91	3,23	0,81	1,95	1,34	1,97
1,6	2,12	0,95	2,16	0,84	2,45	0,76	3,04	1,56	1,7
1,8	1,7	0,72	1,81	0,83	3,19	0,87	3,16	1,47	2,2
1,78	1,89	0,5	1,95	0,93	2,41	0,75	2,46	1,44	2,26
1,91	1,44	0,89	1,87	0,86	3,32	0,86	1,86	1,52	1,24
1,81	1,59	0,69	1,61	0,83	2,36	0,86	1,55	1,15	1,79
1,71	1,31	0,78	1,51	0,85	2,3	0,77	2,28	1,31	2,33
1,94	1,83	0,84	2	0,9	2,4	0,74	2,5	1,23	1,36
1,59	1,36	0,77	1,76	0,75	2,33	0,76	2,48	1,35	1,6
1,67	1,51	1,01	2,8	0,56	2,12	0,83	2,03	1,66	1,39
1,58	1,88	0,89	2,52	0,87	1,81	0,76	2,42	1,52	1,43
1,75	1,86	0,72	1,47	0,78	1,97	0,69	2,87	1,35	2,04
2,04	1,89	0,89	2,49	0,75	3,04	1	2,72	1,19	1,29
1,81	1,78	0,77	2,6	0,96	2,06	0,79	2,12	1,83	1,72
1,75	1,9	0,92	1,77	0,74	1,57	0,61	2,25	1,48	1,94
1,91	2,01	0,82	2,61	0,88	1,69	0,72	2,35	1,29	1,7
	1,86	0,61	2,16	0,52	1,91	0,93	2,46	1,74	2,2
	1,7	0,74	2,44	0,83	2,1	0,92	2,66	1,24	1,35

		0,75		0,73	2,76	0,58<br>0,89<br>0,74	2,58<br>2,05<br>1,77		

Fig 2C Dot plot

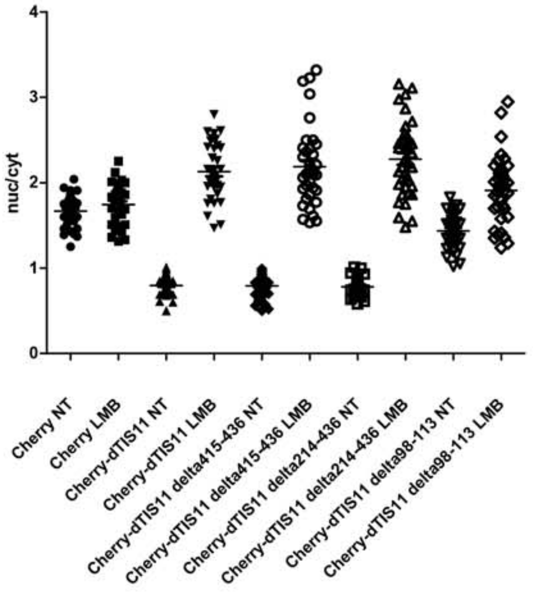

Fig 2C Mean + SD

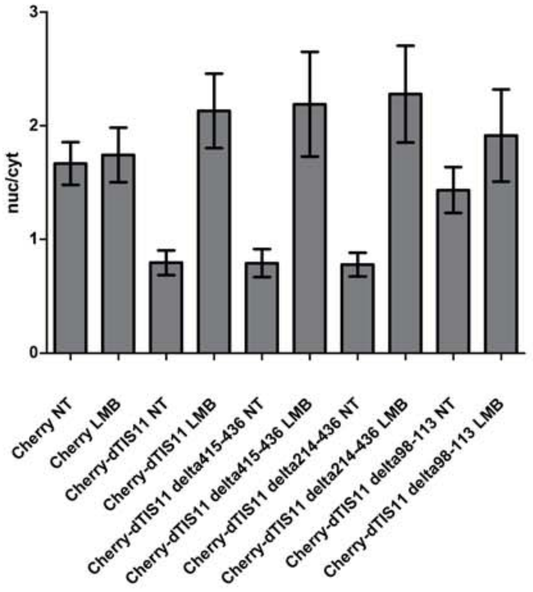

Fig 2C Mean + SEM

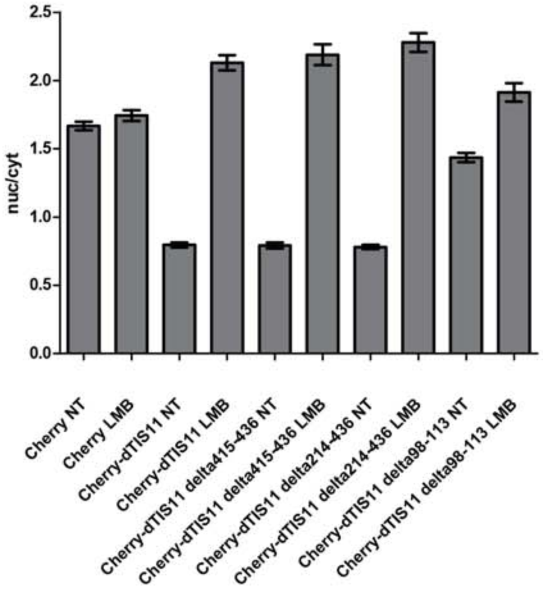

Figure 2C - Statistical analysis

This file can be opened by GraphPad Prism (version 5.00 or later).

This file contains 11 data tables and 0 info tables:

- Col. stats of Fig 2C new
- Cherry NT vs LMB
- Cherry-dTIS11 NT vs LMB
- Cherry-dTIS11delta415-436 NT vs LMB
- Cherry-dTIS11delta214-436 NT vs LMB
- Cherry-dTIS11delta98-113 NT vs LMB
- Cherry NT vs Cherry-dTIS11 NT
- Cherry LMB vs Cherry-dTIS11 LMB
- Cherry-dTIS11 WT vs delta415-436 NT
- Cherry-dTIS11 WT vs delta214-436 NT
- Cherry-dTIS11 WT vs delta98-113 NT

Col. stats of Fig 2C new

	**Cherry NT**	**Cherry LMB**	**Cherry-dTIS11 NT**	**Cherry-dTIS11 LMB**	**Cherry-dTIS11 delta415-436 NT**	**Cherry-dTIS11 delta415-436 LMB**	**Cherry-dTIS11 delta214-436 NT**	**Cherry-dTIS11 delta214-436 LMB**	**Cherry-dTIS11 delta98-113 NT**	**Cherry-dTIS11 delta98-113 LMB**
Number of values	34	36	37	36	37	37	39	39	36	36
Minimum	1.250	1.310	0.5000	1.470	0.5000	1.530	0.5800	1.480	1.020	1.240
25% Percentile	1.543	1.510	0.7300	1.950	0.7350	1.885	0.7000	1.960	1.295	1.625
Median	1.685	1.805	0.7900	2.085	0.8200	2.100	0.7600	2.250	1.455	1.925
75% Percentile	1.803	1.898	0.8850	2.418	0.8850	2.405	0.8600	2.510	1.605	2.148
Maximum	2.040	2.250	1.010	2.800	0.9900	3.320	1.010	3.160	1.830	2.950
Mean	1.667	1.743	0.7957	2.130	0.7916	2.188	0.7800	2.278	1.434	1.913
Std. Deviation	0.1878	0.2397	0.1088	0.3272	0.1242	0.4602	0.1037	0.4259	0.2013	0.4043
Std. Error	0.03221	0.03995	0.01789	0.05453	0.02041	0.07565	0.01660	0.06819	0.03355	0.06739
Lower 95% CI of mean	1.601	1.662	0.7594	2.019	0.7502	2.035	0.7464	2.140	1.366	1.776
Upper 95% CI of mean	1.732	1.824	0.8320	2.240	0.8330	2.342	0.8136	2.417	1.503	2.050
KS normality test										
KS distance	0.1124	0.1416	0.06955	0.1020	0.1264	0.1299	0.1025	0.06256	0.08869	0.07831
P value	> 0.10	0.0657	> 0.10	> 0.10	> 0.10	> 0.10	> 0.10	> 0.10	> 0.10	> 0.10
Passed normality test (alpha=0.05)?	Yes	Yes	Yes	Yes	Yes	Yes	Yes	Yes	Yes	Yes
P value summary	ns	ns	ns	ns	ns	ns	ns	ns	ns	ns
Sum	56.67	62.75	29.44	76.67	29.29	80.97	30.42	88.86	51.64	68.87

Cherry NT vs LMB

	Data Set-A
Table Analyzed	Fig 2C new
Column A	Cherry NT
vs	vs
Column B	Cherry LMB
Unpaired t test	
P value	0.1444
P value summary	ns
Are means signif. different? (P < 0.05)	No
One- or two-tailed P value?	Two-tailed
t, df	t=1.476 df=68
How big is the difference?	
Mean ± SEM of column A	1.667 ± 0.03221 N=34
Mean ± SEM of column B	1.743 ± 0.03995 N=36

Difference between means	-0.07629 ± 0.05167
95% confidence interval	-0.1795 to 0.02692
R squared	0.03106
F test to compare variances	
F,DFn, Dfd	1.629, 35, 33
P value	0.1619
P value summary	ns
Are variances significantly different?	No

Cherry-dTIS11 NT vs LMB

	Data Set-A
Table Analyzed	Fig. 2C
Column C	Cherry-dTIS11 NT
vs	vs
Column D	Cherry-dTIS11 LMB
Unpaired t test with Welch's correction	
P value	< 0.0001
P value summary	***
Are means signif. different? (P < 0.05)	Yes
One- or two-tailed P value?	Two-tailed
Welch-corrected t, df	t=23.24 df=42
How big is the difference?	
Mean ± SEM of column C	0.7957 ± 0.01789 N=37
Mean ± SEM of column D	2.130 ± 0.05453 N=36
Difference between means	-1.334 ± 0.05739
95% confidence interval	-1.450 to -1.218
R squared	0.9279
F test to compare variances	
F,DFn, Dfd	9.041, 35, 36
P value	< 0.0001
P value summary	***
Are variances significantly different?	Yes

Cherry-dTIS11delta415-436 NT vs LMB

	Data Set-A
Table Analyzed	Fig. 2C
Column E	Cherry-dTIS11 delta415-436 NT
vs	vs
Column F	Cherry-dTIS11 delta415-436 LMB
Unpaired t test with Welch's correction	
P value	< 0.0001
P value summary	***
Are means signif. different? (P < 0.05)	Yes
One- or two-tailed P value?	Two-tailed
Welch-corrected t, df	t=17.83 df=41
How big is the difference?	
Mean ± SEM of column E	0.7916 ± 0.02041 N=37
Mean ± SEM of column F	2.188 ± 0.07565 N=37
Difference between means	-1.397 ± 0.07835
95% confidence interval	-1.555 to -1.238
R squared	0.8857
F test to compare variances	
F,DFn, Dfd	13.74, 36, 36
P value	< 0.0001
P value summary	***
Are variances significantly different?	Yes

Cherry-dTIS11delta214-436 NT vs LMB

	Data Set-A
Table Analyzed	Fig 2C
Column G	Cherry-dTIS11 delta214-436 NT
vs	vs
Column H	Cherry-dTIS11 delta214-436 LMB
Unpaired t test with Welch's correction	
P value	< 0.0001
P value summary	***
Are means signif. different? (P < 0.05)	Yes
One- or two-tailed P value?	Two-tailed
Welch-corrected t, df	t=21.35 df=42
How big is the difference?	
Mean ± SEM of column G	0.7800 ± 0.01660 N=39
Mean ± SEM of column H	2.278 ± 0.06819 N=39
Difference between means	-1.498 ± 0.07018
95% confidence interval	-1.640 to -1.357
R squared	0.9156
F test to compare variances	
F,DFn, Dfd	16.87, 38, 38
P value	< 0.0001
P value summary	***
Are variances significantly different?	Yes

Cherry-dTIS11delta98-113 NT vs LMB

	Data Set-A
Table Analyzed	Fig. 2C
Column I	Cherry-dTIS11 delta98-113 NT
vs	vs
Column J	Cherry-dTIS11 delta98-113 LMB
Unpaired t test with Welch's correction	
P value	< 0.0001
P value summary	***
Are means signif. different? (P < 0.05)	Yes
One- or two-tailed P value?	Two-tailed
Welch-corrected t, df	t=6.358 df=51
How big is the difference?	
Mean ± SEM of column I	1.434 ± 0.03355 N=36
Mean ± SEM of column J	1.913 ± 0.06739 N=36
Difference between means	-0.4786 ± 0.07528
95% confidence interval	-0.6299 to -0.3273
R squared	0.4422
F test to compare variances	
F,DFn, Dfd	4.035, 35, 35
P value	< 0.0001
P value summary	***
Are variances significantly different?	Yes

Cherry NT vs Cherry-dTIS11 NT

	Data Set-A
Table Analyzed	Fig 2C new
Column A	Cherry NT
vs	vs
Column C	Cherry-dTIS11 NT
Unpaired t test with Welch's correction	
P value	< 0.0001

P value summary	***
Are means signif. different? (P < 0.05)	Yes
One- or two-tailed P value?	Two-tailed
Welch-corrected t, df	t=23.64 df=51
How big is the difference?	
Mean ± SEM of column A	1.667 ± 0.03221 N=34
Mean ± SEM of column C	0.7957 ± 0.01789 N=37
Difference between means	0.8711 ± 0.03684
95% confidence interval	0.7971 to 0.9451
R squared	0.9164
F test to compare variances	
F,DFn, Dfd	2.978, 33, 36
P value	0.0017
P value summary	**
Are variances significantly different?	Yes

Cherry LMB vs Cherry-dTIS11 LMB

	Data Set-A
Table Analyzed	Fig. 2C
Column B	Cherry LMB
vs	vs
Column D	Cherry-dTIS11 LMB
Unpaired t test	
P value	< 0.0001
P value summary	***
Are means signif. different? (P < 0.05)	Yes
One- or two-tailed P value?	Two-tailed
t, df	t=5.720 df=70
How big is the difference?	
Mean ± SEM of column B	1.743 ± 0.03995 N=36
Mean ± SEM of column D	2.130 ± 0.05453 N=36
Difference between means	-0.3867 ± 0.06760
95% confidence interval	-0.5216 to -0.2517
R squared	0.3185
F test to compare variances	
F,DFn, Dfd	1.863, 35, 35
P value	0.0697
P value summary	ns
Are variances significantly different?	No

Cherry-dTIS11 WT vs delta415-436 NT

	Data Set-A
Table Analyzed	Fig. 2C
Column C	Cherry-dTIS11 NT
vs	vs
Column E	Cherry-dTIS11 delta415-436 NT
Unpaired t test	
P value	0.8817
P value summary	ns
Are means signif. different? (P < 0.05)	No
One- or two-tailed P value?	Two-tailed
t, df	t=0.1494 df=72
How big is the difference?	
Mean ± SEM of column C	0.7957 ± 0.01789 N=37
Mean ± SEM of column E	0.7916 ± 0.02041 N=37
Difference between means	0.004054 ± 0.02714
95% confidence interval	-0.05012 to 0.05823
R squared	0.0003098

F test to compare variances	
F,DFn, Dfd	1.302, 36, 36
P value	0.4329
P value summary	ns
Are variances significantly different?	No

Cherry-dTIS11 WT vs delta214-436 NT

	Data Set-A
Table Analyzed	Fig. 2C
Column C	Cherry-dTIS11 NT
vs	vs
Column G	Cherry-dTIS11 delta214-436 NT
Unpaired t test	
P value	0.5222
P value summary	ns
Are means signif. different? (P < 0.05)	No
One- or two-tailed P value?	Two-tailed
t, df	t=0.6431 df=74
How big is the difference?	
Mean ± SEM of column C	0.7957 ± 0.01789 N=37
Mean ± SEM of column G	0.7800 ± 0.01660 N=39
Difference between means	0.01568 ± 0.02438
95% confidence interval	-0.03296 to 0.06432
R squared	0.005557
F test to compare variances	
F,DFn, Dfd	1.101, 36, 38
P value	0.7684
P value summary	ns
Are variances significantly different?	No

Cherry-dTIS11 WT vs delta98-113 NT

	Data Set-A
Table Analyzed	Fig. 2C
Column C	Cherry-dTIS11 NT
vs	vs
Column I	Cherry-dTIS11 delta98-113 NT
Unpaired t test with Welch's correction	
P value	< 0.0001
P value summary	***
Are means signif. different? (P < 0.05)	Yes
One- or two-tailed P value?	Two-tailed
Welch-corrected t, df	t=16.80 df=53
How big is the difference?	
Mean ± SEM of column C	0.7957 ± 0.01789 N=37
Mean ± SEM of column I	1.434 ± 0.03355 N=36
Difference between means	-0.6388 ± 0.03802
95% confidence interval	-0.7151 to -0.5624
R squared	0.8419
F test to compare variances	
F,DFn, Dfd	3.421, 35, 36
P value	0.0004
P value summary	***
Are variances significantly different?	Yes

Figure 2E - Raw data

This file can be opened by GraphPad Prism (version 5.00 or later).

This file contains 1 data tables and 0 info tables:

- Fig 2E

Fig 2E

Cherry NT	Cherry +LMB	Cherry-dTIS11 97-131 NT	Cherry-dTIS11 97-131 +LMB
2,056	1,984	1,29	1,612
2,112	1,842	1,346	1,977
1,901	1,855	1,322	1,769
2,056	1,829	1,045	1,629
1,879	1,865	1,113	1,62
1,58	1,69	1,152	1,936
1,676	1,974	1,14	1,466
1,654	1,82	1,343	1,841
2,366	1,498	1,233	1,556
2,082	1,976	1,386	1,704
1,784	1,963	1,083	2,028
2,013	2,042	1,391	2,084
1,7	2,195	1,784	1,444
1,579	2,174	1,601	1,77
1,593	1,761	0,932	1,804
2,345	1,909	1,062	1,622
2,101	2,031	1,659	1,591
1,776	1,845	1,133	1,935
1,933	1,913	1,135	1,84
2,004	1,613	1,066	1,68
1,631	2,068	1,064	1,716
1,807	2,062	1,061	1,607
1,862	1,871	1,135	1,7
1,806	2,183	1,428	1,537
1,53	2,031	0,974	2,028
2,068	1,781	1,049	1,319
1,576	2,058	1,257	1,737
1,668	1,577	1,248	1,883
1,711	1,808	0,996	1,445
1,971	1,995	1,021	1,428
2,163	2,086	1,143	1,584
1,667	1,755	0,87	1,468
1,489	2,305	1,15	1,506
1,643	1,831	1,577	1,727
1,529	1,851	1,015	1,862
1,748	1,449	1,05	1,836

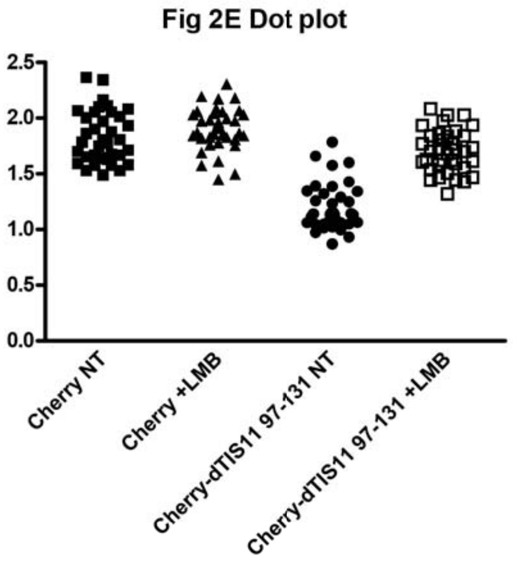

Fig 2E Dot plot
2.5
2.0
1.5
1.0
0.5
0.0
Cherry NT
Cherry +LMB
Cherry-dTIS11 97-131 NT
Cherry-dTIS11 97-131 +LMB

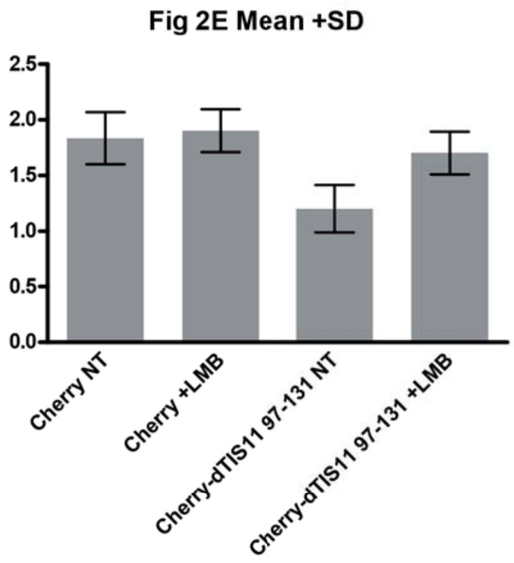

Fig 2E Mean +SD
2.5
2.0
1.5
1.0
0.5
0.0
Cherry NT
Cherry +LMB
Cherry-dTIS11 97-131 NT
Cherry-dTIS11 97-131 +LMB

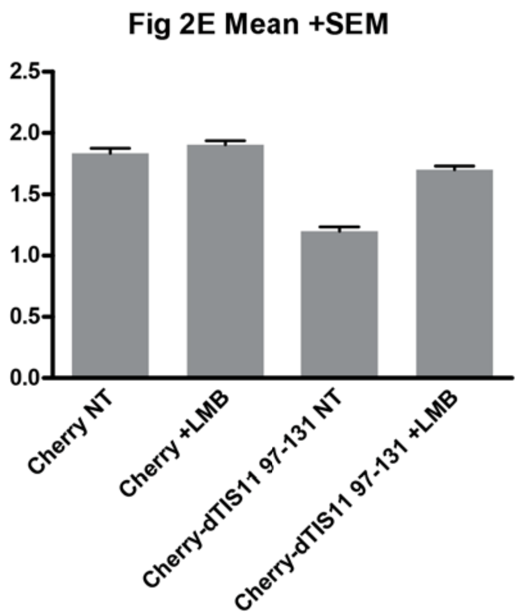

Fig 2E Mean +SEM
2.5
2.0
1.5
1.0
0.5
0.0
Cherry NT
Cherry +LMB
Cherry-dTIS11 97-131 NT
Cherry-dTIS11 97-131 +LMB

Figure 2E - Statistical analysis

This file can be opened by GraphPad Prism (version 5.00 or later).

This file contains 4 data tables and 0 info tables:

- Col Stats of Fig 2E
- Cherry NT vs LMB
- Cherry-dTIS11 97-131 NT vs LMB
- Cherry NT vs Cherry-dTIS11 97-131 NT

Col Stats of Fig 2E

	Cherry NT	Cherry +LMB	Cherry-dTIS11 97-131 NT	Cherry-dTIS11 97-131 +LMB
Number of values	36	36	36	36
Minimum	1.489	1.449	0.8700	1.319
25% Percentile	1.646	1.811	1.053	1.563
Median	1.795	1.890	1.138	1.702
75% Percentile	2.045	2.039	1.338	1.841
Maximum	2.366	2.305	1.784	2.084
Mean	1.835	1.903	1.202	1.703
Std. Deviation	0.2336	0.1923	0.2124	0.1922
Std. Error	0.03893	0.03205	0.03540	0.03204
Lower 95% CI of mean	1.756	1.837	1.130	1.637
Upper 95% CI of mean	1.914	1.968	1.273	1.768
KS normality test				
KS distance	0.1189	0.08933	0.2033	0.09338
P value	> 0.10	> 0.10	0.0006	> 0.10
Passed normality test (alpha=0.05)?	Yes	Yes	No	Yes
P value summary	ns	ns	***	ns
Sum	66.06	68.49	43.25	61.29

Cherry NT vs LMB

	Data Set-A
Table Analyzed	Fig 2E
Column A	Cherry NT
vs	vs
Column B	Cherry +LMB
Mann Whitney test	
P value	0.1342
Exact or approximate P value?	Gaussian Approximation

P value summary	ns
Are medians signif. different? (P < 0.05)	No
One- or two-tailed P value?	Two-tailed
Sum of ranks in column A,B	1181 , 1448
Mann-Whitney U	514.5

Cherry-dTIS11 97-131 NT vs LMB

	Data Set-A
Table Analyzed	Fig 2E
Column C	Cherry-dTIS11 97-131 NT
vs	vs
Column D	Cherry-dTIS11 97-131 +LMB
Mann Whitney test	
P value	< 0.0001
Exact or approximate P value?	Gaussian Approximation
P value summary	***
Are medians signif. different? (P < 0.05)	Yes
One- or two-tailed P value?	Two-tailed
Sum of ranks in column C,D	732.5 , 1896
Mann-Whitney U	66.50

Cherry NT vs Cherry-dTIS11 97-131 NT

	Data Set-A
Table Analyzed	Fig 2E
Column A	Cherry NT
vs	vs
Column C	Cherry-dTIS11 97-131 NT
Mann Whitney test	
P value	< 0.0001
Exact or approximate P value?	Gaussian Approximation
P value summary	***
Are medians signif. different? (P < 0.05)	Yes
One- or two-tailed P value?	Two-tailed
Sum of ranks in column A,C	1924 , 704.5
Mann-Whitney U	38.50

Figure 3B - Raw data

This file can be opened by GraphPad Prism (version 5.00 or later).

This file contains 1 data tables and 0 info tables:

- **Data 1**

Data 1

Cherry NT	Cherry +LMB	Cherry +NaN3	Cherry +LMB+NaN3	Cherry-dTIS11 NT	Cherry-dTIS11 +LMB	Cherry-dTIS11 +NaN3	Cherry-dTIS1 +LMB+NaN3
1,406	1,726	1,538	1,567	0,788	1,773	0,975	1,016
1,478	1,345	1,758	1,706	0,874	1,627	0,928	0,937
1,75	1,52	1,518	1,799	0,604	1,62	0,855	0,921
2,333	1,753	1,358	1,541	0,853	1,835	1,214	1,189
1,913	1,525	1,696	1,672	0,841	1,189	1,009	1,062
1,991	1,591	1,832	1,67	0,673	1,455	1,001	1,132
2,135	1,714	1,777	1,515	0,706	1,555	1,565	1,107
2,1	1,801	1,263	1,811	0,667	1,533	1,159	0,896
1,736	1,243	1,889	2,036	0,838	1,632	0,94	0,854
1,707	1,374	1,284	1,548	0,682	1,213	0,859	0,958
1,698	1,527	1,794	1,645	0,895	1,54	0,961	0,937
2,189	1,76	2,051	1,758	0,917	1,655	1,054	1,016
1,983	1,707	2,021	1,678	0,736	1,467	0,84	1,306
1,463	1,425	2,014	1,442	0,808	1,588	0,972	1,449
1,564	1,664	1,801	1,415	0,698	1,429	0,892	0,888
1,949	1,475	2,004	1,997	0,829	1,517	1,035	1,032
1,899	1,679	1,874	1,738	0,801	1,539	0,931	0,966
1,546	1,843	1,878	1,994	0,846	1,661	0,872	1,051
1,57	1,732	1,667	2,017	0,633	1,49	0,901	1,418
1,934	1,811	1,547	1,691	0,883	2,177	1,029	0,973
2,002	1,778	1,514	1,71	0,606	1,867	0,971	1,024
1,798	1,497	1,486	1,873	0,846	1,344	0,83	0,994
1,883	1,359	1,337	1,987	0,751	1,417	1,218	0,835
1,195	1,952	1,499	1,583	0,812	1,652	0,822	0,889
1,337	1,964	1,522	1,581	0,63	1,558	1,053	0,928
1,845	1,96	1,657	1,283	0,577	1,469	1,087	0,838
1,611	2,021	1,567	1,368	0,798	1,266	0,999	0,925
1,616	1,745	1,741	1,751	0,754	1,473	1,281	0,927
1,644	1,699	1,683	1,708	1,023	1,626	0,799	0,919
1,482	1,716	2,218	1,487	0,782	1,201	0,835	0,957
1,781	1,811	1,585	1,73	0,973	1,417	0,851	1,034
1,651	1,963	1,712	1,584	0,938	1,203	1,052	0,806
1,627	1,714	1,549	1,535	0,812			
2,263		1,66	1,555				
1,463			1,375				
1,644			1,436				
1,457							

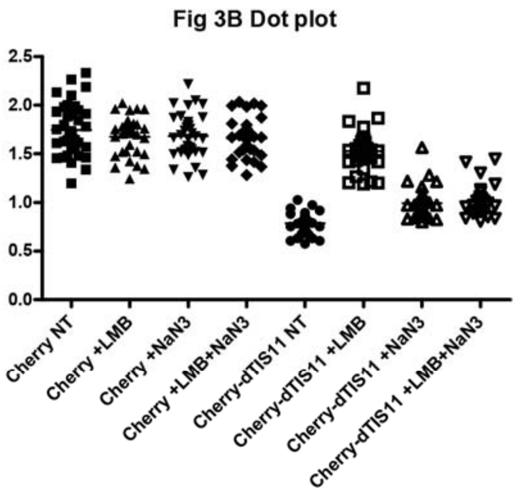
Fig 3B Dot plot
2.5
2.0
1.5
1.0
0.5
0.0
Cherry NT
Cherry +LMB
Cherry +NaN3
Cherry +LMB+NaN3
Cherry-dTIS11 NT
Cherry-dTIS11 +LMB
Cherry-dTIS11 +NaN3
Cherry-dTIS11 +LMB+NaN3

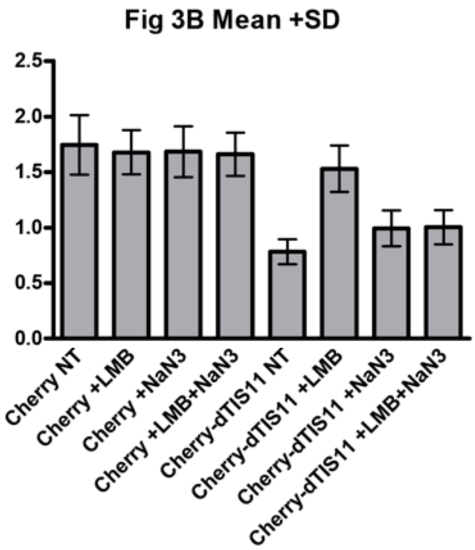
Fig 3B Mean +SD
2.5
2.0
1.5
1.0
0.5
0.0
Cherry NT
Cherry +LMB
Cherry +NaN3
Cherry +LMB+NaN3
Cherry-dTIS11 NT
Cherry-dTIS11 +LMB
Cherry-dTIS11 +NaN3
Cherry-dTIS11 +LMB+NaN3

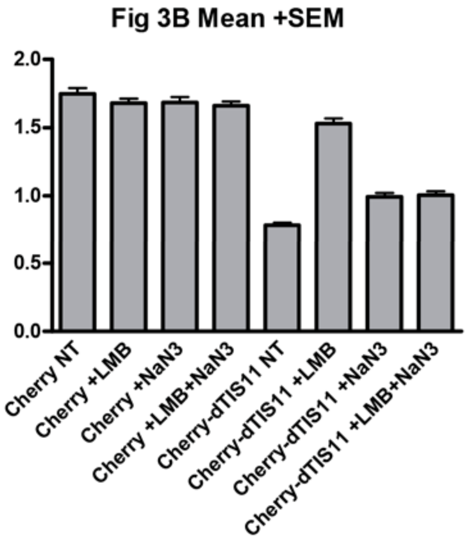
Fig 3B Mean +SEM
2.0
1.5
1.0
0.5
0.0
Cherry NT
Cherry +LMB
Cherry +NaN3
Cherry +LMB+NaN3
Cherry-dTIS11 NT
Cherry-dTIS11 +LMB
Cherry-dTIS11 +NaN3
Cherry-dTIS11 +LMB+NaN3

Figure 3B - Statistical analysis

This file can be opened by GraphPad Prism (version 5.00 or later).

This file contains 8 data tables and 0 info tables:

- Col Stats of Data 1
- Cherry NT vs LMB
- Cherry NT vs NaN3
- Cherry NT vs NaN3+LMB
- Cherry-dTIS11 NT vs LMB
- Cherry-dTIS11 NT vs NaN3
- Cherry-dTIS11 NT vs NaN3+LMB
- Cherry-dTIS11 NaN3 vs NaN3+LMB

Col Stats of Data 1

	Cherry NT	Cherry +LMB	Cherry +NaN3	Cherry +LMB+NaN3	Cherry-dTIS11 NT	Cherry-dTIS11 +LMB	Cherry-dTIS11 +NaN3	Cherry-dTIS1 +LMB+NaN3
Number of values	37	33	34	36	33	32	32	32
Minimum	1.195	1.243	1.263	1.283	0.5770	1.189	0.7990	0.8060
25% Percentile	1.555	1.523	1.521	1.537	0.6900	1.420	0.8623	0.9195
Median	1.707	1.714	1.675	1.671	0.8010	1.536	0.9715	0.9620
75% Percentile	1.942	1.806	1.843	1.756	0.8495	1.631	1.053	1.047
Maximum	2.333	2.021	2.218	2.036	1.023	2.177	1.565	1.449
Mean	1.747	1.679	1.685	1.661	0.7841	1.531	0.9934	1.006
Std. Deviation	0.2680	0.1986	0.2290	0.1944	0.1112	0.2098	0.1612	0.1538
Std. Error	0.04406	0.03456	0.03927	0.03241	0.01935	0.03708	0.02850	0.02720
Lower 95% CI of mean	1.658	1.608	1.605	1.595	0.7446	1.455	0.9353	0.9503
Upper 95% CI of mean	1.836	1.749	1.765	1.727	0.8235	1.607	1.052	1.061
KS normality test								
KS distance	0.09951	0.1470	0.08080	0.09787	0.09867	0.1426	0.1661	0.1772
P value	> 0.10	0.0681	> 0.10	> 0.10	> 0.10	0.0968	0.0250	0.0120
Passed normality test (alpha=0.05)?	Yes	Yes	Yes	Yes	Yes	Yes	No	No
P value summary	ns	ns	ns	ns	ns	ns	*	*
Sum	64.64	55.39	57.29	59.79	25.87	48.99	31.79	32.18

Cherry NT vs LMB

	Data Set-A
Table Analyzed	Data 1
Column A	Cherry NT
vs	vs
Column B	Cherry +LMB
Mann Whitney test	
P value	0.4621
Exact or approximate P value?	Gaussian Approximation
P value summary	ns
Are medians signif. different? (P < 0.05)	No
One- or two-tailed P value?	Two-tailed
Sum of ranks in column A,B	1377 , 1109
Mann-Whitney U	547.5

Cherry NT vs NaN3

	Data Set-A
Table Analyzed	Data 1
Column A	Cherry NT
vs	vs
Column C	Cherry +NaN3
Mann Whitney test	
P value	0.4474
Exact or approximate P value?	Gaussian Approximation
P value summary	ns
Are medians signif. different? (P < 0.05)	No
One- or two-tailed P value?	Two-tailed
Sum of ranks in column A,C	1399 , 1158
Mann-Whitney U	562.5

Cherry NT vs NaN3+LMB

	Data Set-A
Table Analyzed	Data 1
Column A	Cherry NT
vs	vs
Column D	Cherry +LMB+NaN3
Mann Whitney test	
P value	0.2186
Exact or approximate P value?	Gaussian Approximation
P value summary	ns
Are medians signif. different? (P < 0.05)	No
One- or two-tailed P value?	Two-tailed
Sum of ranks in column A,D	1481 , 1220
Mann-Whitney U	554.0

Cherry-dTIS11 NT vs LMB

	Data Set-A
Table Analyzed	Data 1
Column E	Cherry-dTIS11 NT
vs	vs
Column F	Cherry-dTIS11 +LMB
Mann Whitney test	
P value	< 0.0001
Exact or approximate P value?	Gaussian Approximation
P value summary	***
Are medians signif. different? (P < 0.05)	Yes
One- or two-tailed P value?	Two-tailed
Sum of ranks in column E,F	561 , 1584
Mann-Whitney U	0.0000

Cherry-dTIS11 NT vs NaN3

	Data Set-A
Table Analyzed	Data 1

Column E	Cherry-dTIS11 NT
vs	vs
Column G	Cherry-dTIS11 +NaN3
Mann Whitney test	
P value	< 0.0001
Exact or approximate P value?	Gaussian Approximation
P value summary	***
Are medians signif. different? (P < 0.05)	Yes
One- or two-tailed P value?	Two-tailed
Sum of ranks in column E,G	682 , 1463
Mann-Whitney U	121.0

Cherry-dTIS11 NT vs NaN3+LMB

	Data Set-A
Table Analyzed	Data 1
Column E	Cherry-dTIS11 NT
vs	vs
Column H	Cherry-dTIS1 +LMB+NaN3
Mann Whitney test	
P value	< 0.0001
Exact or approximate P value?	Gaussian Approximation
P value summary	***
Are medians signif. different? (P < 0.05)	Yes
One- or two-tailed P value?	Two-tailed
Sum of ranks in column E,H	653 , 1492
Mann-Whitney U	92.00

Cherry-dTIS11 NaN3 vs NaN3+LMB

	Data Set-A
Table Analyzed	Data 1
Column G	Cherry-dTIS11 +NaN3
vs	vs
Column H	Cherry-dTIS1 +LMB+NaN3
Mann Whitney test	
P value	0.7831
Exact or approximate P value?	Gaussian Approximation
P value summary	ns
Are medians signif. different? (P < 0.05)	No
One- or two-tailed P value?	Two-tailed
Sum of ranks in column G,H	1019 , 1061
Mann-Whitney U	491.0

Figure 4B - Raw data

This file can be opened by GraphPad Prism (version 5.00 or later).

This file contains 1 data tables and 0 info tables:

- **Data 1**

Data 1

Cherry NT	Cherry +LMB	Cherry-dTIS11 WT NT	Cherry-dTIS11 WT +LMB	delta131-213 NT	delta131-213 +LMB	C150A NT	C150A +LMB	C156A NT	C156A +LMB	C179A NT	C179A +LMB	C188A NT	C188A +LMB	C194A NT	C194A +LMB	C156A C179A NT	C156A C179A +LMB
1,79	1,77	1	1,68	0,99	1,85	0,59	2,11	0,85	2,08	0,66	2,13	0,81	2,5	0,79	3,15	0,76	5,89
1,78	1,81	0,6	1,84	0,85	1,43	0,65	1,74	0,85	2,35	0,87	2,98	0,94	2,72	0,87	3,63	0,85	4,54
1,42	1,53	0,93	2,4	0,9	1,76	0,83	1,97	0,95	1,98	0,76	3,4	0,88	2,66	0,82	3,33	0,9	2,94
1,54	1,68	0,83	2	0,9	1,74	1,01	1,69	0,75	1,89	0,83	3,53	0,73	2,98	0,9	3,92	0,79	4,58
1,85	1,79	0,82	2,35	1,1	1,95	0,93	1,82	0,81	2,4	0,71	2,96	0,76	4,42	0,69	2,72	0,9	5,28
1,7	1,81	0,84	1,89	0,76	1,93	0,73	1,78	0,93	2,82	0,75	3,04	0,54	3,75	0,86	3,83	0,83	3,31
1,55	1,52	0,72	2,36	0,71	1,49	0,83	3,14	0,92	2,83	0,86	3,47	0,88	6,04	0,85	2,36	0,83	3,19
1,52	1,94	0,69	1,86	0,59	1,65	0,61	2,58	0,76	1,82	0,81	2,63	0,92	6,85	0,74	5,01	0,84	3,49
1,63	1,44	0,67	2,16	0,8	1,88	0,93	1,72	0,99	2,14	0,66	3,87	0,74	2,86	0,75	5,54	0,78	3,74
1,71	2,03	0,75	2,02	0,8	2,11	0,68	2,01	0,87	2	0,79	4,02	0,65	5,53	0,8	2,72	0,75	2,48
1,4	1,79	0,75	2,28	0,59	2,05	0,72	2,41	0,68	1,93	0,88	3,21	0,88	2,54	0,71	5,55	0,96	2,51
1,74	1,81	0,96	2,22	0,96	1,67	0,73	2,13	0,85	2,23	0,82	3,59	0,57	4,01	0,91	6,47	0,85	3,05
1,91	1,57	0,86	2,39	0,94	1,61	0,74	1,98	0,72	2,06	0,81	3,67	0,8	3,33	0,67	6,04	0,93	3,21
1,84	1,62	0,46	2,56	0,83	1,88	0,85	2,34	0,87	1,91	0,81	3,83	0,81	2,42	0,66	2,56	0,9	5,75
2,08	1,4	0,85	1,89	0,89	1,73	0,82	2,27	0,48	1,89	0,82	3,65	0,78	2,05	0,85	2,84	1,01	4,81
1,49	1,76	0,91	2,12	0,76	1,66	0,91	2,2	0,91	2,04	0,79	3,61	0,87	4,07	0,85	3,44	0,7	2,82
1,53	1,61	0,89	1,77	0,92	1,82	0,76	2,02	0,67	2,11	0,81	3,19	0,71	5,84	0,9	3,03	0,82	2,7
1,68	1,88	0,91	1,79	0,65	1,75	0,83	2,28	0,76	2,23	0,62	3,91	0,78	2,72	0,63	3,44	0,77	2,96
1,57	1,8	0,75	2,06	0,86	1,9	0,82	1,98	0,68	2,25	0,96	4,19	0,92	3,03	0,76	4,72	0,64	3,79
1,56	1,73	0,66	2,1	0,77	1,53	0,85	1,31	0,87	2,16	0,95	3,91	0,76	4,09	0,8	3,22	0,97	3,37
1,69	1,59	0,76	2,18	0,75	1,73	0,81	2,17	0,84	2,36	0,8	2,46	0,93	2,79	0,8	4,57	0,75	3,39
1,53	1,45	0,83	2,8	0,78	1,81	0,85	2,41	0,76	2,61	0,97	2,77	0,91	3,55	0,58	3,55	0,84	3,69
1,68	1,9	0,74	2,14	0,91	1,72	0,76	2,37	0,79	2,45	0,82	3,41	0,94	6,34	0,72	4,9	0,85	5,36
1,63	1,7	0,74	2,38	0,96	1,85	0,9	2,12	0,7	1,97	0,81	2,73	0,86	6,27	0,92	1,7	0,76	4,57
1,66	1,72	0,65	1,43	0,88	1,73	0,91	2,18	0,66	1,76	0,91	2,45	0,9	5,61	0,71	2,07	0,86	2,89
1,34	1,65	0,62	2,15	0,63	1,53	0,92	2,04	0,96	2,24	0,82	4,15	0,82	4,79	0,82	4,65	0,66	3,67
1,69	1,5	0,6	2,02	0,72	1,49	0,72	2,2	0,77	2,52	0,8	4,11	0,92	4,66	0,78	4,59	0,76	5,7
1,49	1,62	0,71	1,87	0,88	1,9	0,94	2,64	0,82	2,11	0,83	2,72	1,08	3,33	0,86	9,23	0,8	3,14
1,62	1,78	0,89	2,09	0,74	1,73	0,85	2,08	0,8	1,63	0,79	2,46	0,92	3,79	0,74	2,75	0,88	2,44
1,78	1,67	0,93	2,19	0,9	1,72	0,82	2	0,81	2,43	0,91	2,72	0,9	3,69	0,85	5,08	0,82	5,5
	1,7		2,4				2,37	0,84		0,77	2,35		4,17		4,48	0,8	5,2
	1,93		2,08				2,64						4,82		3,81		3,54
	1,63		2,42				2,3						4,78		4,46		
			2,11				2,2						4,59		2,79		
			2,26										4,3		2,74		
													5,81				

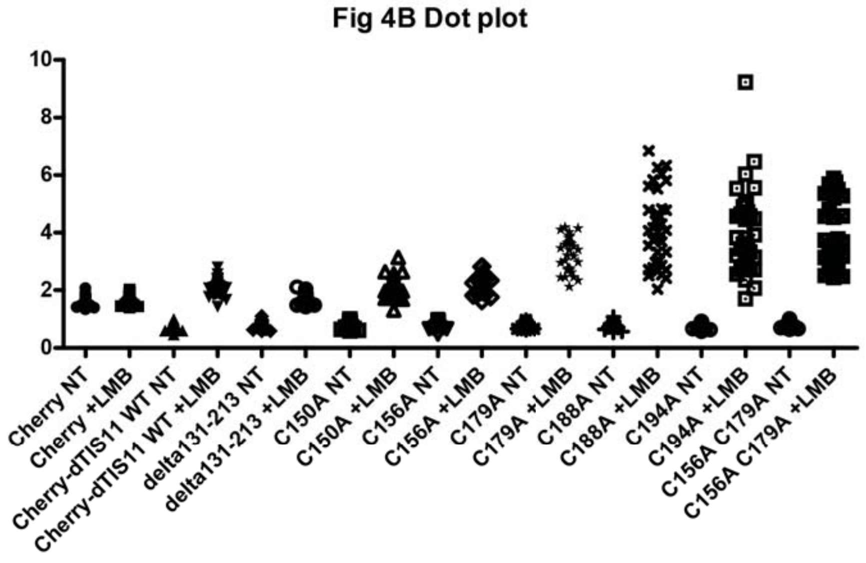
Fig 4B Dot plot
10
8
6
4
2
0
Cherry NT
Cherry +LMB
Cherry-dTIS11 WT NT
Cherry-dTIS11 WT +LMB
delta131-213 NT
delta131-213 +LMB
C150A NT
C150A +LMB
C156A NT
C156A +LMB
C179A NT
C179A +LMB
C188A NT
C188A +LMB
C194A NT
C194A +LMB
C156A C179A NT
C156A C179A +LMB

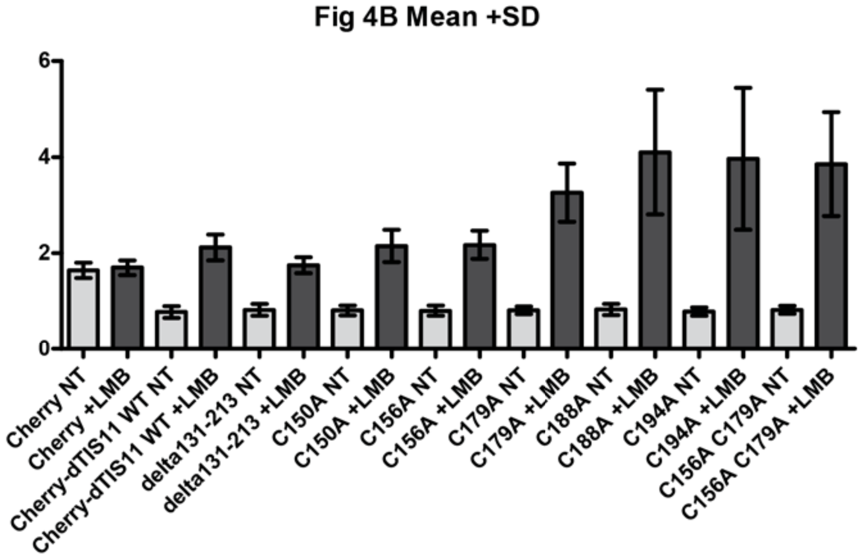
Fig 4B Mean +SD
6
4
2
0
Cherry NT
Cherry +LMB
Cherry-dTIS11 WT NT
Cherry-dTIS11 WT +LMB
delta131-213 NT
delta131-213 +LMB
C150A NT
C150A +LMB
C156A NT
C156A +LMB
C179A NT
C179A +LMB
C188A NT
C188A +LMB
C194A NT
C194A +LMB
C156A C179A NT
C156A C179A +LMB

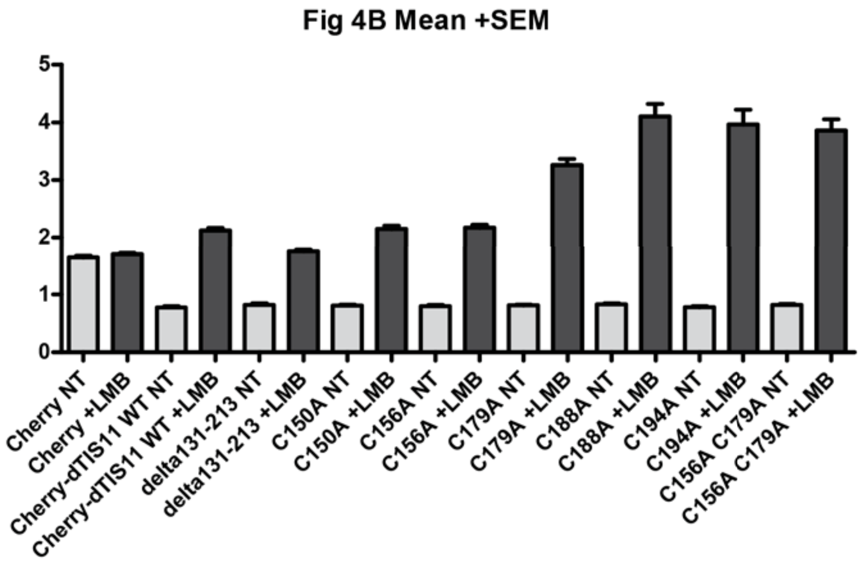
Fig 4B Mean +SEM
5
4
3
2
1
0
Cherry NT
Cherry +LMB
Cherry-dTIS11 WT NT
Cherry-dTIS11 WT +LMB
delta131-213 NT
delta131-213 +LMB
C150A NT
C150A +LMB
C156A NT
C156A +LMB
C179A NT
C179A +LMB
C188A NT
C188A +LMB
C194A NT
C194A +LMB
C156A C179A NT
C156A C179A +LMB

Figure 4B - Statistical analysis

This file can be opened by GraphPad Prism (version 5.00 or later).

This file contains 10 data tables and 0 info tables:

- Col Stats of Data 1
- Cherry +LMB vs Cherry-dTIS11 WT +LMB
- WT +LMB vs delta131-213 +LMB
- Cherry +LMB vs delta131-213 +LMB
- WT +LMB vs C150A +LMB
- WT +LMB vs C156A +LMB
- WT +LMB vs C179A +LMB
- WT +LMB vs C188A +LMB
- WT +LMB vs C194A +LMB
- WT +LMB vs C156A C179A +LMB

Col Stats of Data 1

	Cherry NT	Cherry +LMB	Cherry-dTIS11 WT NT	Cherry-dTIS11 WT +LMB	delta131-213 NT	delta131-213 +LMB
Number of values	30	33	30	35	30	30
Minimum	1.340	1.400	0.4600	1.430	0.5900	1.430
25% Percentile	1.530	1.600	0.6850	1.890	0.7475	1.658
Median	1.645	1.700	0.7550	2.120	0.8400	1.735
75% Percentile	1.750	1.805	0.8900	2.350	0.9025	1.880
Maximum	2.080	2.030	1.000	2.800	1.100	2.110
Mean	1.647	1.701	0.7773	2.122	0.8240	1.753
Std. Deviation	0.1606	0.1531	0.1265	0.2699	0.1216	0.1641
Std. Error	0.02932	0.02666	0.02310	0.04562	0.02220	0.02996
Lower 95% CI of mean	1.587	1.647	0.7301	2.029	0.7786	1.692
Upper 95% CI of mean	1.707	1.755	0.8246	2.214	0.8694	1.815
KS normality test						
KS distance	0.08348	0.08662	0.09869	0.07742	0.1108	0.1195
P value	> 0.10	> 0.10	> 0.10	> 0.10	> 0.10	> 0.10
Passed normality test (alpha=0.05)?	Yes	Yes	Yes	Yes	Yes	Yes
P value summary	ns	ns	ns	ns	ns	ns
Sum	49.40	56.13	23.32	74.26	24.72	52.60

	C150A NT	C150A +LMB	C156A NT	C156A +LMB	C179A NT	C179A +LMB	C188A NT	C188A +LMB	C194A NT
Number of values	30	34	31	30	31	31	30	36	30
Minimum	0.5900	1.310	0.4800	1.630	0.6200	2.130	0.5400	2.050	0.5800
25% Percentile	0.7300	1.980	0.7500	1.960	0.7900	2.720	0.7600	2.890	0.7175
Median	0.8250	2.150	0.8100	2.125	0.8100	3.400	0.8650	4.040	0.8000
75% Percentile	0.9025	2.348	0.8700	2.370	0.8600	3.830	0.9200	4.813	0.8525
Maximum	1.010	3.140	0.9900	2.830	0.9700	4.190	1.080	6.850	0.9200
Mean	0.8100	2.153	0.8039	2.173	0.8129	3.262	0.8303	4.103	0.7863
Std. Deviation	0.1023	0.3331	0.1073	0.2921	0.08162	0.6049	0.1157	1.298	0.0879
Std. Error	0.01868	0.05712	0.01928	0.05333	0.01466	0.1086	0.02113	0.2163	0.0160
Lower 95% CI of mean	0.7718	2.037	0.7645	2.064	0.7830	3.040	0.7871	3.664	0.7535
Upper 95% CI of mean	0.8482	2.269	0.8432	2.282	0.8428	3.484	0.8736	4.542	0.8192
KS normality test									
KS distance	0.1389	0.1150	0.08341	0.09648	0.1637	0.1145	0.1384	0.1014	0.1322
P value	> 0.10	> 0.10	> 0.10	> 0.10	0.0337	> 0.10	> 0.10	> 0.10	> 0.10
Passed normality test (alpha=0.05)?	Yes	Yes	Yes	Yes	No	Yes	Yes	Yes	Yes
P value summary	ns	ns	ns	ns	*	ns	ns	ns	ns
Sum	24.30	73.20	24.92	65.20	25.20	101.1	24.91	147.7	23.59

Cherry +LMB vs Cherry-dTIS11 WT +LMB

	Data Set-A
Table Analyzed	Data 1
Column B	Cherry +LMB
vs	vs
Column D	Cherry-dTIS11 WT +LMB
Mann Whitney test	
P value	< 0.0001
Exact or approximate P value?	Gaussian Approximation
P value summary	***
Are medians signif. different? (P < 0.05)	Yes
One- or two-tailed P value?	Two-tailed
Sum of ranks in column B,D	660 , 1686
Mann-Whitney U	99.00

WT +LMB vs delta131-213 +LMB

	Data Set-A
Table Analyzed	Data 1
Column D	Cherry-dTIS11 WT +LMB
vs	vs
Column F	delta131-213 +LMB
Mann Whitney test	
P value	< 0.0001
Exact or approximate P value?	Gaussian Approximation
P value summary	***
Are medians signif. different? (P < 0.05)	Yes
One- or two-tailed P value?	Two-tailed
Sum of ranks in column D,F	1557 , 588
Mann-Whitney U	123.0

Cherry +LMB vs delta131-213 +LMB

	Data Set-A
Table Analyzed	Data 1
Column B	Cherry +LMB
vs	vs
Column F	delta131-213 +LMB
Mann Whitney test	
P value	0.2077
Exact or approximate P value?	Gaussian Approximation
P value summary	ns
Are medians signif. different? (P < 0.05)	No
One- or two-tailed P value?	Two-tailed
Sum of ranks in column B,F	964 , 1052
Mann-Whitney U	403.0

WT +LMB vs C150A +LMB

	Data Set-A
Table Analyzed	Data 1
Column D	Cherry-dTIS11 WT +LMB
vs	vs
Column H	C150A +LMB
Mann Whitney test	
P value	0.7779
Exact or approximate P value?	Gaussian Approximation
P value summary	ns
Are medians signif. different? (P < 0.05)	No
One- or two-tailed P value?	Two-tailed
Sum of ranks in column D,H	1201 , 1214
Mann-Whitney U	571.0

WT +LMB vs C156A +LMB

	Data Set-A
Table Analyzed	Data 1
Column D	Cherry-dTIS11 WT +LMB
vs	vs
Column J	C156A +LMB
Mann Whitney test	
P value	0.6262
Exact or approximate P value?	Gaussian Approximation
P value summary	ns
Are medians signif. different? (P < 0.05)	No
One- or two-tailed P value?	Two-tailed
Sum of ranks in column D,J	1118 , 1028
Mann-Whitney U	487.5

WT +LMB vs C179A +LMB

	Data Set-A
Table Analyzed	Data 1
Column D	Cherry-dTIS11 WT +LMB
vs	vs
Column L	C179A +LMB
Mann Whitney test	
P value	< 0.0001
Exact or approximate P value?	Gaussian Approximation
P value summary	***
Are medians signif. different? (P < 0.05)	Yes
One- or two-tailed P value?	Two-tailed
Sum of ranks in column D,L	666.5 , 1545
Mann-Whitney U	36.50

WT +LMB vs C188A +LMB

	Data Set-A
Table Analyzed	Data 1
Column D	Cherry-dTIS11 WT +LMB
vs	vs
Column N	C188A +LMB
Mann Whitney test	
P value	< 0.0001
Exact or approximate P value?	Gaussian Approximation
P value summary	***
Are medians signif. different? (P < 0.05)	Yes
One- or two-tailed P value?	Two-tailed

Sum of ranks in column D,N	663.5 , 1893
Mann-Whitney U	33.50

WT +LMB vs C194A +LMB

	Data Set-A
Table Analyzed	Data 1
Column D	Cherry-dTIS11 WT +LMB
vs	vs
Column P	C194A +LMB
Mann Whitney test	
P value	< 0.0001
Exact or approximate P value?	Gaussian Approximation
P value summary	***
Are medians signif. different? (P < 0.05)	Yes
One- or two-tailed P value?	Two-tailed
Sum of ranks in column D,P	699 , 1786
Mann-Whitney U	69.00

WT +LMB vs C156A C179A +LMB

	Data Set-A
Table Analyzed	Data 1
Column D	Cherry-dTIS11 WT +LMB
vs	vs
Column R	C156A C179A +LMB
Mann Whitney test	
P value	< 0.0001
Exact or approximate P value?	Gaussian Approximation
P value summary	***
Are medians signif. different? (P < 0.05)	Yes
One- or two-tailed P value?	Two-tailed
Sum of ranks in column D,R	637 , 1641
Mann-Whitney U	7.000

Figure 4D - Raw data

This file can be opened by GraphPad Prism (version 5.00 or later).

This file contains 1 data tables and 0 info tables:

- **Data 1**

Data 1

Cherry	Cherry-dTIS11 114-240 WT	Cherry-dTIS11 114-240 C179A
1,261	1,635	2,179
1,889	1,294	2,532
1,453	1,575	2,363
1,572	1,483	2,088
2,298	1,611	1,879
2,175	1,552	1,784
1,829	1,623	1,595
1,893	1,968	1,925
1,375	1,532	2,434
1,741	1,425	1,636
1,66	1,612	3,185
1,536	1,641	1,939
1,679	2,135	2,13
1,579	1,707	1,896
1,57	2,607	2,715
1,591	1,719	2,773
2,103	1,744	2,093
1,932	1,947	1,986
1,76	1,738	1,804
1,953	1,387	2,853
1,665	1,707	2,915
1,488	1,746	2,265
1,773	1,719	1,929
1,866	1,824	2,058
2,016	1,637	1,969
1,562	2,019	2,595
1,629	1,491	1,714
2,157	1,439	1,419
1,721	1,363	2,046
2,863	1,567	2,114
2,353	1,519	2,557
1,328	1,292	2,47
1,778	1,479	2,554
1,796	1,54	1,815
2,031	1,492	2,124

Fig 4D Dot plot

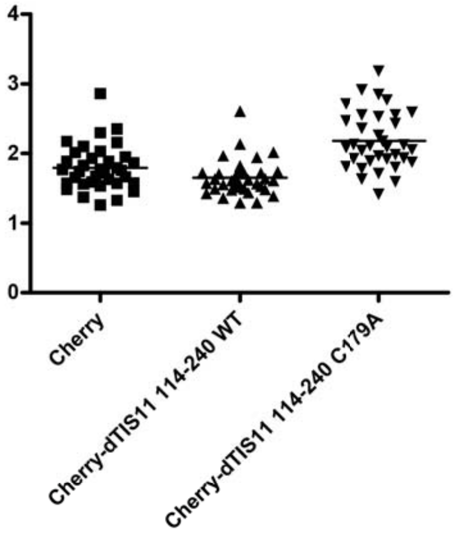

Fig 4D Mean +SD

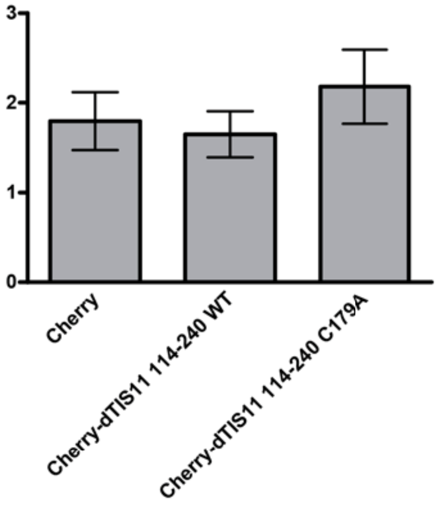

Fig 4D Mean +SEM

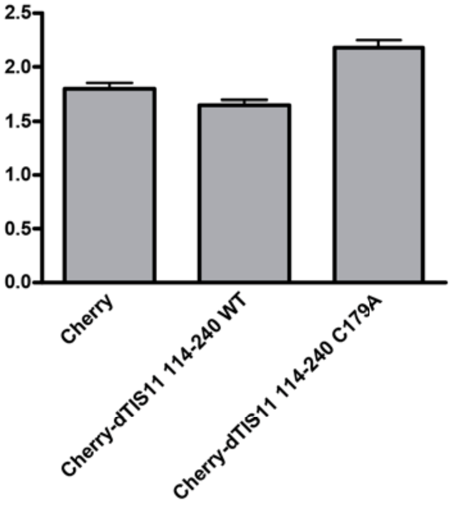

Figure 4D - Statistical analysis

This file can be opened by GraphPad Prism (version 5.00 or later).

This file contains 3 data tables and 0 info tables:

- Col Stats of Data 1
- Cherry vs Cherry-dTIS11 114-240 WT
- Cherry vs Cherry-dTIS11 114-240 C179A

Col Stats of Data 1

	Cherry	Cherry-dTIS11 114-240 WT	Cherry-dTIS11 114-240 C179A
Number of values	35	35	35
Minimum	1.261	1.292	1.419
25% Percentile	1.572	1.491	1.896
Median	1.760	1.612	2.093
75% Percentile	1.953	1.738	2.532
Maximum	2.863	2.607	3.185
Mean	1.796	1.651	2.181
Std. Deviation	0.3225	0.2559	0.4121
Std. Error	0.05451	0.04326	0.06965
Lower 95% CI of mean	1.686	1.563	2.039
Upper 95% CI of mean	1.907	1.738	2.322
KS normality test			
KS distance	0.1005	0.1832	0.1492
P value	> 0.10	0.0044	0.0469
Passed normality test (alpha=0.05)?	Yes	No	No
P value summary	ns	**	*
Sum	62.88	57.77	76.33

Cherry vs Cherry-dTIS11 114-240 WT

	Data Set-A
Table Analyzed	Data 1
Column A	Cherry
vs	vs
Column B	Cherry-dTIS11 114-240 WT
Mann Whitney test	
P value	0.0200
Exact or approximate P value?	Gaussian Approximation
P value summary	*
Are medians signif. different? (P < 0.05)	Yes

One- or two-tailed P value?	Two-tailed
Sum of ranks in column A,B	1441 , 1044
Mann-Whitney U	414.0

Cherry vs Cherry-dTIS11 114-240 C179A

	Data Set-A
Table Analyzed	Data 1
Column A	Cherry
vs	vs
Column C	Cherry-dTIS11 114-240 C179A
Mann Whitney test	
P value	< 0.0001
Exact or approximate P value?	Gaussian Approximation
P value summary	***
Are medians signif. different? (P < 0.05)	Yes
One- or two-tailed P value?	Two-tailed
Sum of ranks in column A,C	894 , 1591
Mann-Whitney U	264.0

Figure 5C - Raw data

This file can be opened by GraphPad Prism (version 5.00 or later).

This file contains 1 data tables and 0 info tables:

- **Data 1**

Data 1

NT	Cdm	Imp4	KapB3	Ketel	Msk	Rbp9	Rbp11	Trn	Trn-SR
2,021	1,451	2,278	2,156	2,436	1,884	2,054	1,612	1,636	1,459
1,959	2,164	2,413	2,035	1,756	2,093	2,191	2,092	1,88	1,77
2,566	2,535	2,523	2,3	2,685	2,525	1,949	2,515	1,752	1,941
2,009	2,482	1,913	2,232	2,309	1,879	2,001	3,148	1,709	1,979
2,971	2,021	1,501	1,198	1,756	4,627	2,503	2,78	1,769	3,25
3,326	2,482	3,196	1,38	3,332	1,571	2,22	1,508	1,626	2,629
2,265	1,657	2,133	2,25	2,484	2,23	1,326	2,125	1,547	2,461
2,073	1,446	2,57	2,804	2,745	2,425	2,266	2,155	1,632	2,034
2,128	2,216	1,734	2,171	2,576	2,492	1,723	2,558	1,082	5,6
2,64	1,755	2,236	2,538	1,971	2,478	2,233	3,146	1,999	3,171
2,43	1,572	2,335	3,027	1,873	2,87	2,921	1,842	2,791	1,934
3,318	3,114	1,159	3,278	3,033	2,317	3,904	2,521	1,729	2,193
2,177	2,365	1,798	1,765	2,436	2,036	2,196	2,113	1,957	2,055
3,125	3,553	2,907	2,272	1,693	1,858	3,076	1,831	1,857	3,196
2,491	1,952	2,185	2,544	4,208	1,612	1,983	2,308	1,889	1,873
2,53	1,972	3,318	2,73	3,594	2,097	2,358	2,91	1,751	2,194
2,706	1,87	2,28	1,882	2,501	1,937	2,919	2,455	1,588	1,749
2,497	1,873	2,459	2,571	2,901	2,114	2,189	2,169	2,051	1,868
3,221	3,335	2,174	2,547	1,471	3,44	1,919	2,256	1,461	1,572
2,648	2,342	2,698	2,051	2,955	2,411	2,309	3,601	1,701	1,398
2,856	2,387	2,296	2,612	2,407	2,991	2,021	3,13	1,823	2,156
2,971	2,078	2,085	3,036	1,916	2,594	4,069	1,541	1,92	1,844
2,525	3,047	1,947	2,434	2,08	2,115	2,466	1,906	2,055	1,906
1,758	1,98	2,072	2,096	2,538	1,887	2,229	1,713	1,973	2,093
2,133	2,184	1,623	2,091	2,759	2,01	2,26	3,148	2,069	2,706
2,133	2,524	1,981	1,521	3,792	1,933	2,442	2,51	2,379	1,8
1,299	1,929	2,223	1,971	2,813	2,286	2,718	2,355	1,003	2,66
1,995	2,306	2,591	2,875	2,555	2,601	2,699	2,158	1,382	2,186
1,786	2,45	2,373	1,389	2,346	1,374	2,209	1,834	1,542	1,432
2,185	2,379	1,993	2,262	2,582	2,141	2,232	2,295	1,274	1,631
2,225	1,63	2,755			2,29	2,35	2,897	1,47	2,879
1,737	2,982	2,647				2,206		2,325	2,507
3,344	2,092					2,273		1,705	1,766
	2,349					1,458		1,817	2,58
	2,13					2,069			2,447
	1,569								
	3,116								

Fig 5C Dot plot

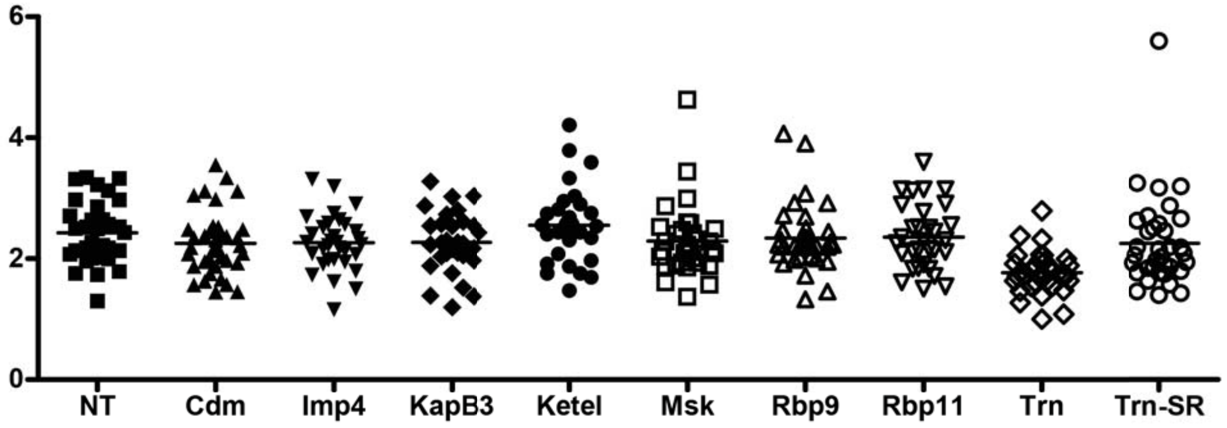

Fig 5C Mean +SD

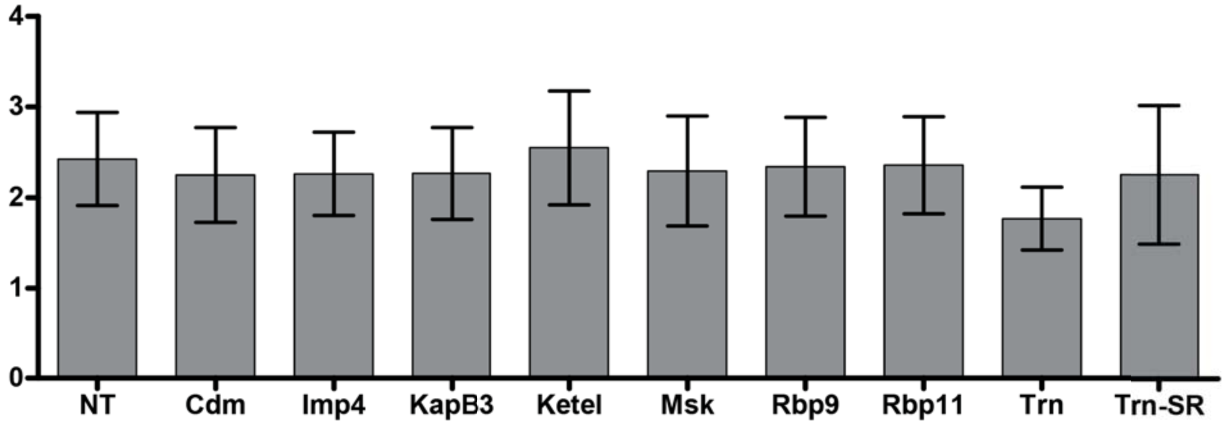

Fig 5C Mean +SEM

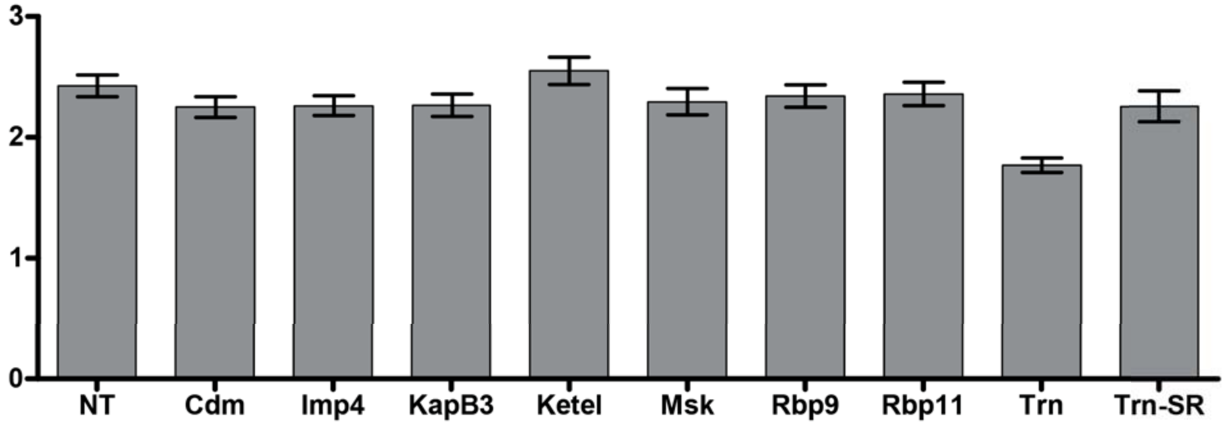

Figure 5C - Statistical analysis

This file can be opened by GraphPad Prism (version 5.00 or later).

This file contains 10 data tables and 0 info tables:

- **Col Stats of Data 1**
- **NT vs Ketel**
- **NT vs Cdm**
- **NT vs Imp4**
- **NT vs KapB3**
- **NT vs Trn**
- **NT vs Trn-SR**
- **NT vs Msk**
- **NT vs Rbp9**
- **NT vs Rbp11**

Col Stats of Data 1

	**NT**	**Cdm**	**Imp4**	**KapB3**	**Ketel**	**Msk**	**Rbp9**	**Rbp11**	**Trn**	**Trn-SR**
Number of values	33	37	32	30	30	31	35	31	34	35
Minimum	1.299	1.446	1.159	1.198	1.471	1.374	1.326	1.508	1.003	1.398
25% Percentile	2.047	1.901	1.984	2.019	2.053	1.933	2.054	1.906	1.578	1.800
Median	2.430	2.184	2.257	2.256	2.520	2.141	2.232	2.295	1.752	2.055
75% Percentile	2.781	2.482	2.558	2.581	2.835	2.492	2.466	2.780	1.961	2.580
Maximum	3.344	3.553	3.318	3.278	4.208	4.627	4.069	3.601	2.791	5.600
Mean	2.426	2.251	2.262	2.267	2.550	2.294	2.341	2.359	1.769	2.255
Std. Deviation	0.5149	0.5236	0.4582	0.5072	0.6273	0.6061	0.5466	0.5358	0.3487	0.7648
Std. Error	0.08964	0.08607	0.08099	0.09261	0.1145	0.1089	0.09240	0.09623	0.05980	0.1293
Lower 95% CI of mean	2.243	2.076	2.097	2.078	2.316	2.072	2.153	2.163	1.647	1.992
Upper 95% CI of mean	2.608	2.426	2.428	2.457	2.784	2.516	2.529	2.556	1.891	2.518
KS normality test										
KS distance	0.1074	0.1316	0.06664	0.09018	0.1131	0.1773	0.2020	0.09717	0.1065	0.1888
P value	> 0.10	> 0.10	> 0.10	> 0.10	> 0.10	0.0142	0.0009	> 0.10	> 0.10	0.0028
Passed normality test (alpha=0.05)?	Yes	Yes	Yes	Yes	Yes	No	No	Yes	Yes	No
P value summary	ns	ns	ns	ns	ns	*	***	ns	ns	**
Geometric mean	2.371	2.194	2.215	2.207	2.478	2.231	2.286	2.301	1.735	2.162
Lower 95% CI of geo. mean	2.194	2.034	2.050	2.016	2.262	2.048	2.120	2.117	1.616	1.964
Upper 95% CI of geo. mean	2.563	2.368	2.393	2.417	2.714	2.429	2.464	2.501	1.862	2.379
Sum	80.05	83.29	72.40	68.02	76.50	71.12	81.94	73.13	60.14	78.92

NT vs Ketel

	Data Set-A
Table Analyzed	Data 1
Column A	NT
vs	vs
Column E	Ketel
Mann Whitney test	
P value	0.5222
Exact or approximate P value?	Gaussian Approximation

P value summary	ns
Are medians signif. different? (P < 0.05)	No
One- or two-tailed P value?	Two-tailed
Sum of ranks in column A,E	1009 , 1007
Mann-Whitney U	448.0

NT vs Cdm

	Data Set-A
Table Analyzed	Data 1
Column A	NT
vs	vs
Column B	Cdm
Mann Whitney test	
P value	0.1057
Exact or approximate P value?	Gaussian Approximation
P value summary	ns
Are medians signif. different? (P < 0.05)	No
One- or two-tailed P value?	Two-tailed
Sum of ranks in column A,B	1310 , 1176
Mann-Whitney U	472.5

NT vs Imp4

	Data Set-A
Table Analyzed	Data 1
Column A	NT
vs	vs
Column C	Imp4
Mann Whitney test	
P value	0.2563
Exact or approximate P value?	Gaussian Approximation
P value summary	ns
Are medians signif. different? (P < 0.05)	No
One- or two-tailed P value?	Two-tailed
Sum of ranks in column A,C	1176 , 969
Mann-Whitney U	441.0

NT vs KapB3

	Data Set-A
Table Analyzed	Data 1
Column A	NT
vs	vs
Column D	KapB3
Mann Whitney test	
P value	0.4208
Exact or approximate P value?	Gaussian Approximation
P value summary	ns
Are medians signif. different? (P < 0.05)	No
One- or two-tailed P value?	Two-tailed
Sum of ranks in column A,D	1115 , 901
Mann-Whitney U	436.0

NT vs Trn

	Data Set-A
Table Analyzed	Data 1
Column A	NT
vs	vs
Column I	Trn
Mann Whitney test	
P value	< 0.0001
Exact or approximate P value?	Gaussian Approximation
P value summary	***
Are medians signif. different? (P < 0.05)	Yes
One- or two-tailed P value?	Two-tailed
Sum of ranks in column A,I	1543 , 735
Mann-Whitney U	140.0

NT vs Trn-SR

	Data Set-A
Table Analyzed	Data 1
Column A	NT
vs	vs
Column J	Trn-SR
Mann Whitney test	
P value	0.0503
Exact or approximate P value?	Gaussian Approximation
P value summary	ns
Are medians signif. different? (P < 0.05)	No
One- or two-tailed P value?	Two-tailed
Sum of ranks in column A,J	1299 , 1048
Mann-Whitney U	417.5

NT vs Msk

	Data Set-A
Table Analyzed	Data 1
Column A	NT
vs	vs
Column F	Msk
Mann Whitney test	
P value	0.1487
Exact or approximate P value?	Gaussian Approximation
P value summary	ns
Are medians signif. different? (P < 0.05)	No
One- or two-tailed P value?	Two-tailed
Sum of ranks in column A,F	1181 , 899.5
Mann-Whitney U	403.5

NT vs Rbp9

	Data Set-A
Table Analyzed	Data 1

Column A<br>vs<br>Column G	NT<br>vs<br>Rbp9
Mann Whitney test	
P value	0.4653
Exact or approximate P value?	Gaussian Approximation
P value summary	ns
Are medians signif. different? (P < 0.05)	No
One- or two-tailed P value?	Two-tailed
Sum of ranks in column A,G	1199 , 1148
Mann-Whitney U	517.5

NT vs Rbp11

	Data Set-A
Table Analyzed	Data 1
Column A<br>vs<br>Column H	NT<br>vs<br>Rbp11
Mann Whitney test	
P value	0.5545
Exact or approximate P value?	Gaussian Approximation
P value summary	ns
Are medians signif. different? (P < 0.05)	No
One- or two-tailed P value?	Two-tailed
Sum of ranks in column A,H	1117 , 963
Mann-Whitney U	467.0

Figure 5E - Raw data

This file can be opened by GraphPad Prism (version 5.00 or later).

This file contains 1 data tables and 0 info tables:

- **Fig 5E new**

Fig 5E new

WT GFP	WT GFP-M9M	C150A GFP	C150A GFP-M9M	C156A GFP	C156A GFP-M9M	C179A GFP	C179A GFP-M9M	C188A GFP	C188A GFP-M9M	C194A GFP	C194A GFP-M9M
2,1	2,15	2,18	2,06	2,68	1,3	2,29	1,8	3,68	2,1	2,56	2,13
2,38	1,61	2,29	2,04	2,4	1,82	3,91	2,07	4,21	2,03	3,36	2,3
1,86	1,77	1,25	2,26	2,37	2,34	2,53	2,56	3,13	2,12	4,08	1,97
1,83	1,82	1,47	2,21	2,59	1,75	2,1	2,17	2,93	2,01	3,8	1,91
1,92	1,86	1,64	2,31	2,18	2,35	2,7	1,61	4,53	2	4,29	1,87
1,73	1,48	2,54	1,79	2,05	2,76	3,21	2,57	2,63	2,38	4,8	1,99
1,86	1,7	2,33	1,99	2,01	3,19	3,5	1,16	1,55	2,97	2,09	2,12
1,98	1,59	1,85	2,09	2,03	1,67	4,25	1,68	4,08	2,24	3,49	1,59
1,88	1,08	1,81	1,92	1,99	2,03	3,46	2,62	4,81	2,25	4,15	2,23
1,92	1,65	2,02	2,62	1,72	1,91	3,28	1,72	2,79	2,07	3,3	1,88
1,55	2,24	2,15	2,07	2,37	2,11	3,76	1,65	2,72	1,92	2,34	2,44
1,36	2,07	1,88	2,23	2,02	1,89	2,49	2,36	3,2	1,92	4,14	2,52
1,84	2,06	1,89	1,79	2,14	1,98	2,73	2,28	4,18	2,53	2,88	2,16
2,01	1,84	2,04	2,08	2,81	1,5	3,26	2,23	3,28	1,72	3,62	1,96
1,85	1,4	2,3	2,13	2,13	1,85	3	1,69	2,82	2,26	2,25	1,39
1,5	1,26	2,64	1,9	2,69	2,09	2,77	2,04	2,7	1,81	3,7	1,91
1,87	1,18	2,33	1,95	2,26	2,21	3,39	1,91	3,16	1,87	2,42	2,57
1,73	1,73	1,8	1,39	2,13	1,5	2,79	0,95	3,59	1,8	3,72	2,46
1,66	2,6	2,37	1,67	1,41	1,51	2,68	2,7	4,17	1,88	3,93	2,2
1,53	1,82	2,25	1,54	1,98	1,69	2,91	1,37	3,87	2,09	3,39	1,61
1,97	2,09	2,27	2,02	2,65	2,45	4,97	2,44	2,69	2,03	3,08	1,95
1,98	2,15	1,97	1,91	2,38	1,28	3,62	2,12	1,22	1,63	2,33	2,53
1,82	1,6	2,11	1,79	2,16	1,57	2,84	2,24	4,05	1,38	4,57	1,82
1,59	2,02	2,16	1,38	2,16	2,45	3,88	2,07	2,34	2,27	2,42	2,06
1,49	2,27	2,13	2,1	2,08	2,16	1,75	2,18	4,84	2,83	2,48	1,9
1,97	1,98	2,38	2,01	2,02	1,07	2,43	1,87	5,56	2,24	5,08	2,1
1,77	2,09	2,33	2,06	2,34	2,04	3,41	1,95	2,4	2,33	4,35	2,32
1,84	2,1	2,04	1,83	1,89	1,92	3,65	1,87	3,19	2,26	3,07	2,42
1,92	3,08	1,96	2,81	1,67	2,55	1,8	1,78	5,17	1,98	4	2,24
2,56	1,94	2,06	2,62	1,72	1,82	2,25	1,86	3,36	2,3	1,66	2,63
1,86	1,91	2,22	1,77	2	1,82	2,95	1,85	2,67	1,96	2,55	2,49
1,59	1,92	1,88	2,23	2,49	1,88	2,52	1,72	1,09	1,87	4,6	2,77
1,63	2	2,13	1,76	2,1	1,58	3,07		2,85		3,38	1,83
1,18	2,22		1,98	2,01	2,61	3,27		4,49		2,71	1,54
	2			2,32	2,33			5,81		2,4	2,21
				2,3	1,9			3,84			2,09
					2,22						

Figure 5E Dot plot

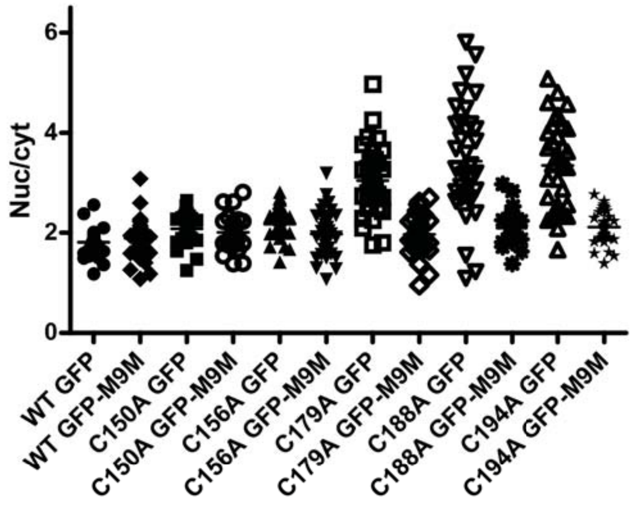

Figure 5E Mean +SD

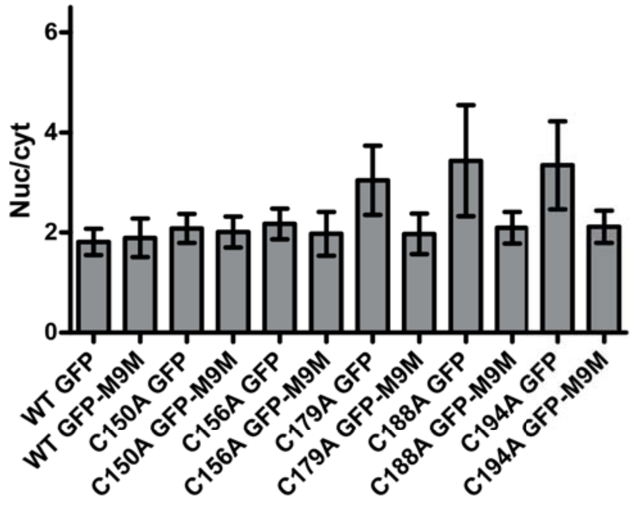

Figure 5E Mean +SEM

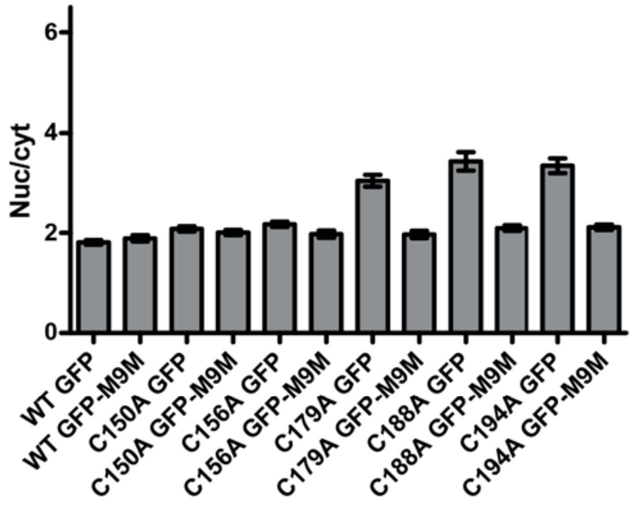

Figure 5E- Statistical analysis

This file can be opened by GraphPad Prism (version 5.00 or later).

This file contains 7 data tables and 0 info tables:

- Col Stats of Fig 5E new
- WT GFP vs GFP-M9M
- C150A GFP vs GFP-M9M
- C156A GFP vs GFP-M9M
- C179A GFP vs GFP-M9M
- C188A GFP vs GFP-M9M
- C194A GFP vs GFP-M9M

Col Stats of Fig 5E new

	WT GFP	WT GFP-M9M	C150A GFP	C150A GFP-M9M	C156A GFP	C156A GFP-M9M	C179A GFP	C179A GFP-M9M	C188A GFP	C188A GFP-M9M	C194A GFP	C194A GFP-M9M
Number of values	34	35	33	34	36	37	34	32	36	32	35	36
Minimum	1.180	1.080	1.250	1.380	1.410	1.070	1.750	0.9500	1.090	1.380	1.660	1.390
25% Percentile	1.620	1.650	1.885	1.790	2.010	1.680	2.528	1.720	2.705	1.890	2.480	1.903
Median	1.845	1.920	2.130	2.015	2.135	1.910	2.975	1.930	3.240	2.050	3.380	2.110
75% Percentile	1.933	2.090	2.295	2.150	2.370	2.275	3.470	2.238	4.178	2.260	4.080	2.395
Maximum	2.560	3.080	2.640	2.810	2.810	3.190	4.970	2.700	5.810	2.970	5.080	2.770
Mean	1.810	1.894	2.081	2.009	2.174	1.976	3.042	1.972	3.433	2.095	3.343	2.114
Std. Deviation	0.2632	0.3848	0.2905	0.3098	0.3051	0.4395	0.6905	0.4054	1.109	0.3176	0.8794	0.3257
Std. Error	0.04514	0.06504	0.05056	0.05313	0.05085	0.07225	0.1184	0.07167	0.1848	0.05615	0.1486	0.05428
Lower 95% CI of mean	1.718	1.762	1.978	1.901	2.070	1.829	2.801	1.825	3.058	1.981	3.040	2.004
Upper 95% CI of mean	1.902	2.026	2.184	2.117	2.277	2.122	3.283	2.118	3.809	2.210	3.645	2.224
KS normality test												
KS distance	0.1412	0.1098	0.09099	0.1199	0.1240	0.09095	0.05610	0.09253	0.09554	0.1050	0.1275	0.07614
P value	0.0835	> 0.10	> 0.10	> 0.10	> 0.10	> 0.10	> 0.10	> 0.10	> 0.10	> 0.10	> 0.10	> 0.10
Passed normality test (alpha=0.05)?	Yes	Yes	Yes	Yes	Yes	Yes	Yes	Yes	Yes	Yes	Yes	Yes
P value summary	ns	ns	ns	ns	ns	ns	ns	ns	ns	ns	ns	ns
Sum	61.53	66.28	68.67	68.31	78.25	73.10	103.4	63.09	123.6	67.05	117.0	76.11

WT GFP vs GFP-M9M

	Data Set-A
Table Analyzed	Fig 5E new
Column A	WT GFP
vs	vs
Column B	WT GFP-M9M
Unpaired t test with Welch's correction	
P value	0.2929
P value summary	ns
Are means signif. different? (P < 0.05)	No
One- or two-tailed P value?	Two-tailed
Welch-corrected t, df	t=1.061 df=60
How big is the difference?	
Mean ± SEM of column A	1.810 ± 0.04514 N=34
Mean ± SEM of column B	1.894 ± 0.06504 N=35
Difference between means	-0.08401 ± 0.07917
95% confidence interval	-0.2424 to 0.07433
R squared	0.01842
F test to compare variances	
F,DFn, Dfd	2.137, 34, 33
P value	0.0316
P value summary	*
Are variances significantly different?	Yes

C150A GFP vs GFP-M9M

	Data Set-A
Table Analyzed	Data 1
Column B	C150A GFP
vs	vs
Column H	C150A GFP-M9M
Unpaired t test	
P value	0.3318
P value summary	ns
Are means signif. different? (P < 0.05)	No
One- or two-tailed P value?	Two-tailed
t, df	t=0.9779 df=65
How big is the difference?	
Mean ± SEM of column B	2.081 ± 0.05056 N=33
Mean ± SEM of column H	2.009 ± 0.05313 N=34
Difference between means	0.07179 ± 0.07342
95% confidence interval	-0.07492 to 0.2185
R squared	0.01450
F test to compare variances	
F,DFn, Dfd	1.138, 33, 32
P value	0.7167
P value summary	ns
Are variances significantly different?	No

C156A GFP vs GFP-M9M

	Data Set-A
Table Analyzed	Data 1
Column C	C156A GFP
vs	vs
Column I	C156A GFP-M9M
Unpaired t test with Welch's correction	
P value	0.0285
P value summary	*
Are means signif. different? (P < 0.05)	Yes
One- or two-tailed P value?	Two-tailed
Welch-corrected t, df	t=2.240 df=64
How big is the difference?	
Mean ± SEM of column C	2.174 ± 0.05085 N=36
Mean ± SEM of column I	1.976 ± 0.07225 N=37
Difference between means	0.1979 ± 0.08835
95% confidence interval	0.02136 to 0.3745
R squared	0.07272
F test to compare variances	
F,DFn, Dfd	2.075, 36, 35
P value	0.0331
P value summary	*
Are variances significantly different?	Yes

C179A GFP vs GFP-M9M

	Data Set-A
Table Analyzed	Data 1
Column G	C179A GFP
vs	vs
Column H	C179A GFP-M9M
Unpaired t test with Welch's correction	
P value	< 0.0001
P value summary	***
Are means signif. different? (P < 0.05)	Yes
One- or two-tailed P value?	Two-tailed
Welch-corrected t, df	t=7.731 df=53

How big is the difference?	
Mean ± SEM of column G	3.042 ± 0.1184 N=34
Mean ± SEM of column H	1.972 ± 0.07167 N=32
Difference between means	1.070 ± 0.1384
95% confidence interval	0.7923 to 1.348
R squared	0.5300
F test to compare variances	
F,DFn, Dfd	2.901, 33, 31
P value	0.0037
P value summary	**
Are variances significantly different?	Yes

C188A GFP vs GFP-M9M

	Data Set-A
Table Analyzed	Data 1
Column E	C188A GFP
vs	vs
Column K	C188A GFP-M9M
Unpaired t test with Welch's correction	
P value	< 0.0001
P value summary	***
Are means signif. different? (P < 0.05)	Yes
One- or two-tailed P value?	Two-tailed
Welch-corrected t, df	t=6.927 df=41
How big is the difference?	
Mean ± SEM of column E	3.433 ± 0.1848 N=36
Mean ± SEM of column K	2.095 ± 0.05615 N=32
Difference between means	1.338 ± 0.1932
95% confidence interval	0.9478 to 1.728
R squared	0.5392
F test to compare variances	
F,DFn, Dfd	12.19, 35, 31
P value	< 0.0001
P value summary	***
Are variances significantly different?	Yes

C194A GFP vs GFP-M9M

	Data Set-A
Table Analyzed	Data 1
Column F	C194A GFP
vs	vs
Column L	C194A GFP-M9M
Unpaired t test with Welch's correction	
P value	< 0.0001
P value summary	***
Are means signif. different? (P < 0.05)	Yes
One- or two-tailed P value?	Two-tailed
Welch-corrected t, df	t=7.763 df=42
How big is the difference?	
Mean ± SEM of column F	3.343 ± 0.1486 N=35
Mean ± SEM of column L	2.114 ± 0.05428 N=36
Difference between means	1.228 ± 0.1582
95% confidence interval	0.9089 to 1.548
R squared	0.5893
F test to compare variances	
F,DFn, Dfd	7.292, 34, 35
P value	< 0.0001
P value summary	***
Are variances significantly different?	Yes

Figure 6B - Raw data

This file can be opened by GraphPad Prism (version 5.00 or later).

This file contains 1 data tables and 0 info tables:

- Fig. 6B

Fig. 6B

C179A	C179A P189A Y190A	C179A P378A Y379A	R135A K137A C179A	R166A R170A K173A K175A C179A	WT
3,92	1,85	4,94	3,84	1,81	2,08
3,3	1,67	4,92	2,95	1,84	1,99
4,89	1,79	2,93	2,8	1,74	1,91
4,26	2,2	2,54	4,1	1,81	2,56
2,99	1,94	2,57	2,95	1,86	1,79
4,05	1,56	4,07	2,54	2,09	1,73
4,74	1,52	2,47	4,2	2,15	1,34
2,37	1,8	3,09	2,75	1,99	1,32
2,44	1,89	5,04	2,53	2,23	1,66
3,8	1,68	3,26	3,49	2,2	1,45
3,62	1,95	2,79	3,19	2,05	1,75
3,84	2,06	2,85	2,89	1,76	1,76
3,03	1,73	2,9	2,84	1,89	2,34
2,64	1,35	2,12	2,85	1,65	1,77
2,81	1,5	3,51	2,9	1,75	2,74
3,76	2,01	4,9	3,39	1,69	2,32
3,92	1,6	4,41	3,01	1,77	1,47
2,63	1,82	2,81	2,49	1,97	1,78
3,03	1,99	4,09	3,17	1,82	2,18
3,49	1,54	2,71	3,02	2,05	1,95
3,87	1,62	3,44	3,4	2	1,74
3,84	1,32	3,19	2,92	1,96	1,67
2,96	1,89	3,71	2,42	2,26	2,13
3,33	2,13	4,71	3,56	1,78	1,98
2,89	2,01	4,67	2,55	1,82	1,89
2,97	1,76	2,48	3,07	1,89	1,66
3,63	1,87	2,23	3,32	2,05	1,63
3,54	1,82	4,11	3,97	1,75	1,78
3,79	1,58	4,66	3,41	1,69	2,02
4,69	1,65	4,39	2,96	1,6	2,28
4,24	1,6	4,06	2,83	2,31	1,64
3,41	1,68	2,89	3,96	2,18	1,71
4,08	1,32	3,97	2,99	1,75	1,72
4,34	1,62	3,27	4,26	2,31	1,45
2,41	1,88	2,22	3,62	1,89	1,72
2,61	2	3,34	3,08	2,12	1,59
3,87	1,73	2,64	2,65	1,98	
3,34	1,82	2,96	2,67	2,05	

		2,75	2,4	1,65<br>1,9	

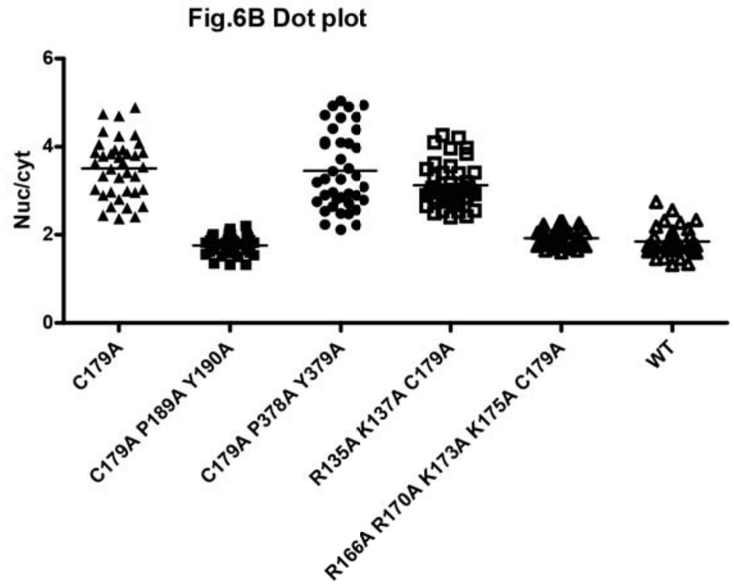
Fig.6B Dot plot
Nuc/cyt
0
2
4
6
C179A
C179A P189A Y190A
C179A P378A Y379A
R135A K137A C179A
R166A R170A K173A K175A C179A
WT

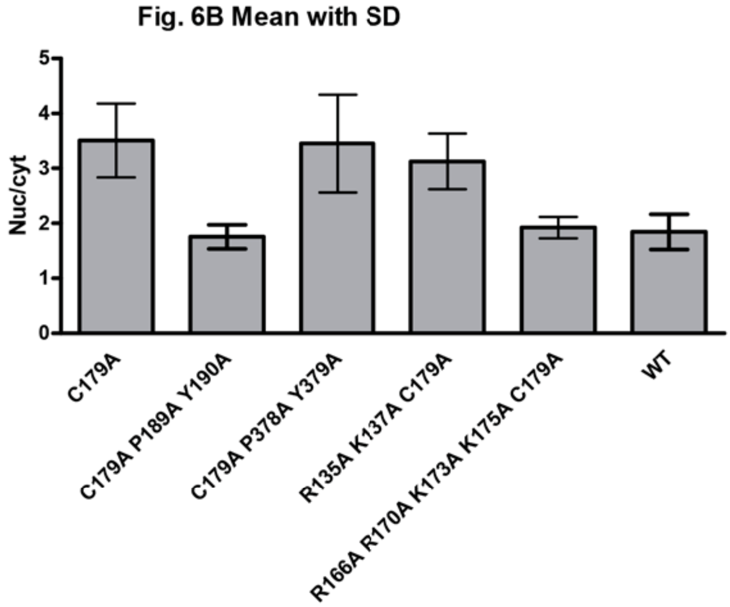
Fig. 6B Mean with SD
Nuc/cyt
0
1
2
3
4
5
C179A
C179A P189A Y190A
C179A P378A Y379A
R135A K137A C179A
R166A R170A K173A K175A C179A
WT

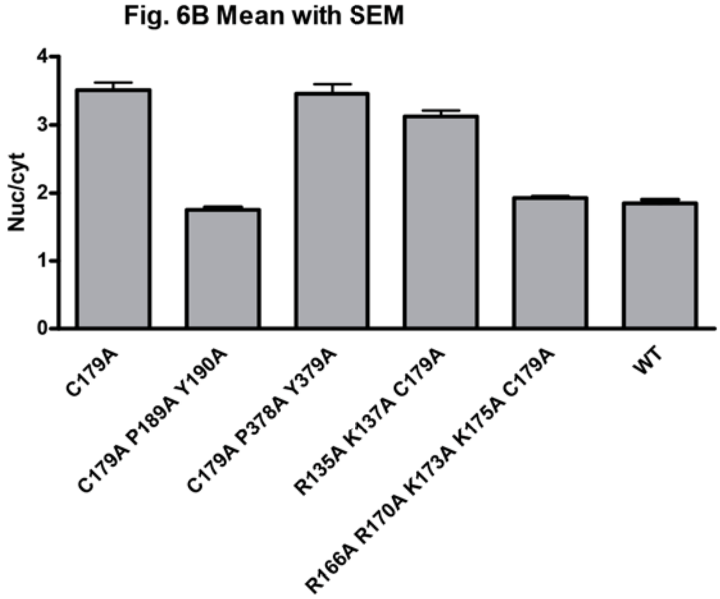
Fig. 6B Mean with SEM
Nuc/cyt
0
1
2
3
4
C179A
C179A P189A Y190A
C179A P378A Y379A
R135A K137A C179A
R166A R170A K173A K175A C179A
WT

Figure 6B - Statistical analysis

This file can be opened by GraphPad Prism (version 5.00 or later).

This file contains 6 data tables and 0 info tables:

- Col Stats of Fig. 6B
- C179A vs C179A P189A Y190A
- C179A vs C179A P378A Y379A
- C179A vs R135A K137A C179A
- C179A vs R166A R170A K173A K175A C179A
- WT vs R166A R170A K173A K175A C179A

Col Stats of Fig. 6B

	C179A	C179A P189A Y190A	C179A P378A Y379A	R135A K137A C179A	R166A R170A K173A K175A C179A	WT
Number of values	38	38	39	39	40	36
Minimum	2.370	1.320	2.120	2.400	1.600	1.320
25% Percentile	2.968	1.600	2.750	2.800	1.763	1.660
Median	3.580	1.775	3.260	2.990	1.890	1.765
75% Percentile	3.920	1.903	4.110	3.410	2.050	2.013
Maximum	4.890	2.200	5.040	4.260	2.310	2.740
Mean	3.509	1.757	3.452	3.127	1.927	1.847
Std. Deviation	0.6696	0.2164	0.8867	0.5082	0.1939	0.3230
Std. Error	0.1086	0.03510	0.1420	0.08138	0.03066	0.05383
Lower 95% CI of mean	3.289	1.685	3.164	2.962	1.864	1.738
Upper 95% CI of mean	3.729	1.828	3.739	3.291	1.989	1.956
KS normality test						
KS distance	0.1049	0.06264	0.1462	0.1520	0.1085	0.1814
P value	> 0.10	> 0.10	0.0349	0.0236	> 0.10	0.0042
Passed normality test (alpha=0.05)?	Yes	Yes	No	No	Yes	No
P value summary	ns	ns	*	*	ns	**
Sum	133.3	66.75	134.6	121.9	77.06	66.50

C179A vs C179A P189A Y190A

	Data Set-A
Table Analyzed	Fig. 6B
Column A	C179A
vs	vs
Column B	C179A P189A Y190A

Mann Whitney test	
P value	< 0.0001
Exact or approximate P value?	Gaussian Approximation
P value summary	***
Are medians signif. different? (P < 0.05)	Yes
One- or two-tailed P value?	Two-tailed
Sum of ranks in column A,B	2185 , 741
Mann-Whitney U	0.0000

C179A vs C179A P378A Y379A

	Data Set-A
Table Analyzed	Fig. 6B
Column A	C179A
vs	vs
Column C	C179A P378A Y379A
Mann Whitney test	
P value	0.5962
Exact or approximate P value?	Gaussian Approximation
P value summary	ns
Are medians signif. different? (P < 0.05)	No
One- or two-tailed P value?	Two-tailed
Sum of ranks in column A,C	1535 , 1469
Mann-Whitney U	688.5

C179A vs R135A K137A C179A

	Data Set-A
Table Analyzed	Fig. 6B
Column A	C179A
vs	vs
Column D	R135A K137A C179A
Mann Whitney test	
P value	0.0118
Exact or approximate P value?	Gaussian Approximation
P value summary	*
Are medians signif. different? (P < 0.05)	Yes
One- or two-tailed P value?	Two-tailed
Sum of ranks in column A,D	1730 , 1274
Mann-Whitney U	493.5

C179A vs R166A R170A K173A K175A C179A

	Data Set-A

Table Analyzed	Fig. 6B
Column A	C179A
vs	vs
Column E	R166A R170A K173A K175A C179A
Mann Whitney test	
P value	< 0.0001
Exact or approximate P value?	Gaussian Approximation
P value summary	***
Are medians signif. different? (P < 0.05)	Yes
One- or two-tailed P value?	Two-tailed
Sum of ranks in column A,E	2261 , 820
Mann-Whitney U	0.0000

WT vs R166A R170A K173A K175A C179A

	Data Set-A
Table Analyzed	Data 1
Column E	R166A R170A K173A K175A C179A
vs	vs
Column F	WT
Mann Whitney test	
P value	0.0562
Exact or approximate P value?	Gaussian Approximation
P value summary	ns
Are medians signif. different? (P < 0.05)	No
One- or two-tailed P value?	Two-tailed
Sum of ranks in column E,F	1724 , 1202
Mann-Whitney U	536.0

Figure 7B - Raw data

This file can be opened by GraphPad Prism (version 5.00 or later).

This file contains 1 data tables and 0 info tables:

- **Fig 7B**

Fig 7B

Cherry	Cherry+LMB	Cherry-TTP WT	Cherry-TTP WT+LMB	C139A	C139A+LMB	C139A P149A Y150A	C139A P149A Y150A+LMB	R126A R130A C139A P149A Y150A	R126A R130A C139A P149A Y150A+
2,26	2,01	0,71	3,13	1,28	16,18	0,76	6,43	1,06	2,22
2,73	2,8	0,74	4,08	0,86	7,65	1,44	2,91	0,95	2,31
2,81	2,44	1	6,05	0,79	8,72	1,11	2,61	0,99	2,72
2,2	3,4	1,12	5,02	0,96	4,28	1,04	3,5	1,15	1,52
2,11	2,5	1,04	6,53	1,09	6,45	1,19	2,66	0,96	2,01
2,31	2,13	1,09	5,92	1,18	3,42	0,92	4,47	0,84	2,01
2,5	2,38	1,16	7,71	0,98	11,49	0,9	3,49	0,77	1,64
2,66	2,59	0,93	5,58	0,82	10,54	1,3	2,93	1,09	2,08
2,14	2,48	1,43	6,13	1,1	6,02	1	3,01	1,01	2,37
1,77	2,61	1,49	2,38	1,09	7,74	0,96	4,17	0,92	2,04
1,45	2,41	1,17	5,54	1,28	7,26	0,94	3,41	0,96	3,06
1,97	2,75	0,83	7,28	1,03	8,06	1,02	2,81	0,9	2,49
2,07	1,66	0,9	3,65	1,07	14,53	1,3	3,78	0,87	2,57
3,08	2,39	0,95	6,02	0,77	8,05	1,3	2,23	0,89	1,63
1,9	2,08	1,22	5,92	1,11	10,46	1,08	2,98	0,84	2,64
2,06	2,78	1,31	7,95	0,73	4,52	1,13	2,45	0,76	2,25
2,2	2,73	1,07	9,53	1,06	4,19	0,9	3,94	1,15	2,07
2,12	2,38	1,26	5,46	1,44	4,66	1,03	3,49	0,91	1,97
2,05	3,05	0,7	4,92	1,32	4,98	1,39	2,57	1,18	2,73
2,41	2,13	0,59	5,79	1,15	5,82	1,2	2,73	1,04	2,19
2,12	2,24	1,01	6,17	0,9	4,21	0,83	4,03	1,29	2,09
2,29	2,07	1,17	6,15	0,87	4,21	1,28	2,9	0,75	2,92
3,11	2,68	1,08	3,06	1,38	13,39	1,11	3,45	0,85	1,76
2,38	2,42	0,95	4,37	0,99	8,95	1	4,27	0,83	1,64
2,62	1,96	1,41	2,03	0,75	8,04	1,52	3,59	0,78	1,82
2,14	2	1,03	6,57	0,77	5,17	0,97	4,73	0,96	1,96
2,55	2,76	1,17	3,07	1,07	8,5	1,19	3,25	1,09	1,9
2,62	2,57	1,2	2,75	1,24	7,15	0,69	3,28	1,14	1,9
2,16	2,49	1,12	2	0,85	5,88	1,15	3,04	0,85	2,06
1,87	2,03	1,32	2,61	1,16	5,02	1,4	3,14	0,68	1,87
	1,93	1,02	5,52		6,01				
		1,02	4,88		4				
		1,01	3,16						
		0,99							

Fig 7B Mean +SEM

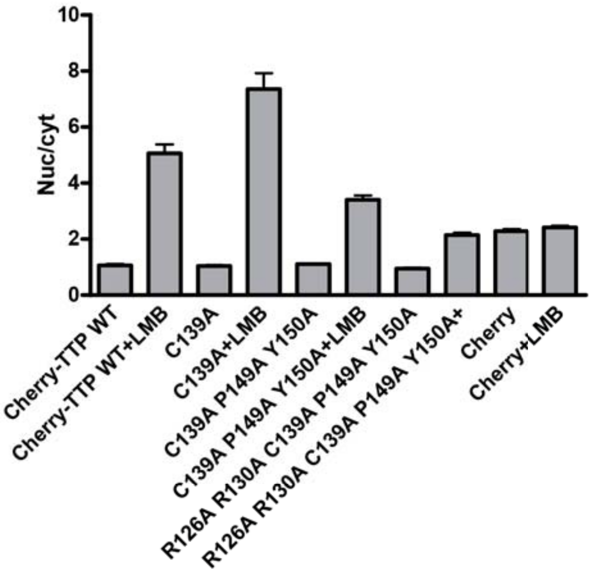

Fig 7B Mean+SD

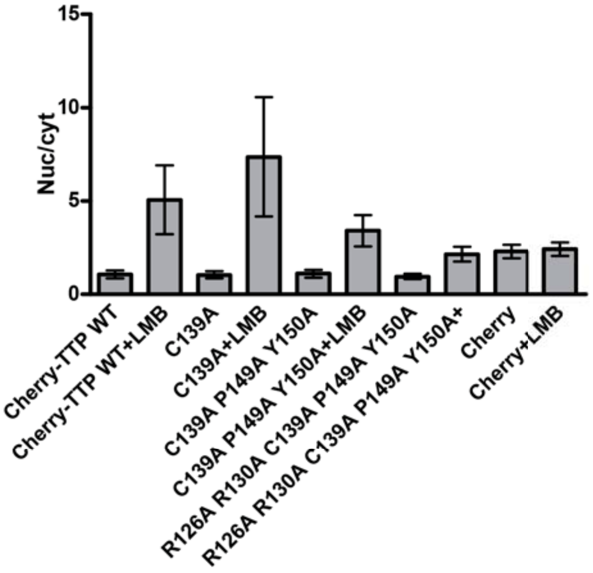

Fig 7B Dot plot

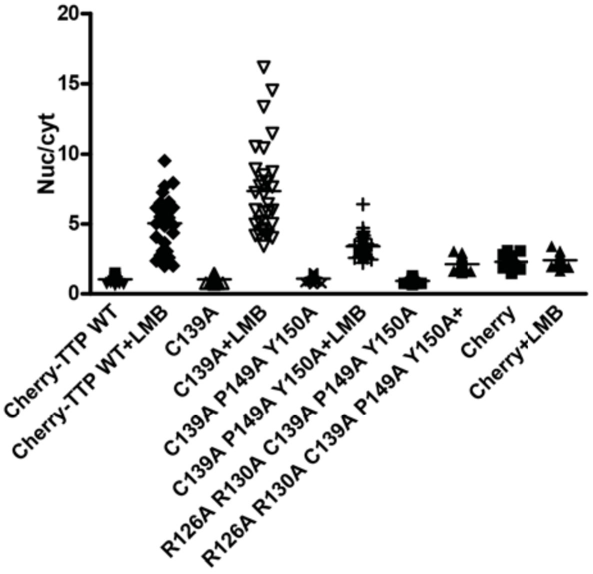

Figure 7B - Statistical analysis

This file can be opened by GraphPad Prism (version 5.00 or later).

This file contains 5 data tables and 0 info tables:

- Col Stats of Fig 7B
- WT+L vs C139A +L
- C139A+L vs C139A P149A Y150A +L
- C139A+L vs R126A R130A C139A P149A Y150A+L
- Cherry vs Cherry+L

Col Stats of Fig 7B

	Cherry	Cherry+LMB	Cherry-TTP WT	Cherry-TTP WT+LMB	C139A	C139A+LMB	C139A P149A Y150A	C139A P149A Y150A+LMB	R126A R130A C139A P149A Y150A	R126A R130A C139A P149A Y150A+
Number of values	30	31	34	33	30	32	30	30	30	30
Minimum	1.450	1.660	0.5900	2.000	0.7300	3.420	0.6900	2.230	0.6800	1.520
25% Percentile	2.068	2.080	0.9500	3.145	0.8575	4.740	0.9550	2.878	0.8400	1.893
Median	2.200	2.420	1.055	5.520	1.065	6.800	1.095	3.265	0.9350	2.065
75% Percentile	2.568	2.680	1.178	6.140	1.165	8.665	1.285	3.820	1.068	2.400
Maximum	3.110	3.400	1.490	9.530	1.440	16.18	1.520	6.430	1.290	3.060
Mean	2.289	2.415	1.065	5.058	1.036	7.361	1.102	3.408	0.9487	2.148
Std. Deviation	0.3707	0.3692	0.2079	1.845	0.1980	3.194	0.2047	0.8416	0.1478	0.3939
Std. Error	0.06768	0.06632	0.03566	0.3212	0.03615	0.5646	0.03736	0.1537	0.02698	0.07192
Lower 95% CI of mean	2.150	2.279	0.9925	4.404	0.9624	6.209	1.025	3.094	0.8935	2.001
Upper 95% CI of mean	2.427	2.550	1.138	5.713	1.110	8.512	1.178	3.723	1.004	2.295
KS normality test										
KS distance	0.1279	0.1079	0.09446	0.1316	0.09953	0.1321	0.08508	0.1566	0.1028	0.1585
P value	> 0.10	> 0.10	> 0.10	> 0.10	> 0.10	> 0.10	> 0.10	0.0583	> 0.10	0.0525
Passed normality test (alpha=0.05)?	Yes	Yes	Yes	Yes	Yes	Yes	Yes	Yes	Yes	Yes
P value summary	ns	ns	ns	ns	ns	ns	ns	ns	ns	ns
Sum	68.66	74.85	36.21	166.9	31.09	235.6	33.05	102.3	28.46	64.44

WT+L vs C139A +L

	Data Set-A
Table Analyzed	Fig 7B
Column D	Cherry-TTP WT+LMB
vs	vs
Column F	C139A+LMB
Unpaired t test with Welch's correction	
P value	0.0009
P value summary	***
Are means signif. different? (P < 0.05)	Yes
One- or two-tailed P value?	Two-tailed
Welch-corrected t, df	t=3.545 df=49
How big is the difference?	
Mean ± SEM of column D	5.058 ± 0.3212 N=33
Mean ± SEM of column F	7.361 ± 0.5646 N=32
Difference between means	-2.302 ± 0.6495
95% confidence interval	-3.609 to -0.9959
R squared	0.2041
F test to compare variances	
F,DFn, Dfd	2.997, 31, 32
P value	0.0028
P value summary	**
Are variances significantly different?	Yes

C139A+L vs C139A P149A Y150A +L

	Data Set-A
Table Analyzed	Fig 7B
Column F	C139A+LMB
vs	vs
Column H	C139A P149A Y150A+LMB
Unpaired t test with Welch's correction	
P value	< 0.0001
P value summary	***
Are means signif. different? (P < 0.05)	Yes
One- or two-tailed P value?	Two-tailed
Welch-corrected t, df	t=6.755 df=35
How big is the difference?	
Mean ± SEM of column F	7.361 ± 0.5646 N=32
Mean ± SEM of column H	3.408 ± 0.1537 N=30
Difference between means	3.953 ± 0.5851
95% confidence interval	2.764 to 5.141
R squared	0.5659
F test to compare variances	
F,DFn, Dfd	14.40, 31, 29
P value	< 0.0001
P value summary	***
Are variances significantly different?	Yes

C139A+L vs R126A R130A C139A P149A Y150A+L

	Data Set-A
Table Analyzed	Fig 7B
Column F	C139A+LMB
vs	vs
Column J	R126A R130A C139A P149A Y150A+
Unpaired t test with Welch's correction	
P value	< 0.0001
P value summary	***
Are means signif. different? (P < 0.05)	Yes
One- or two-tailed P value?	Two-tailed
Welch-corrected t, df	t=9.159 df=32
How big is the difference?	
Mean ± SEM of column F	7.361 ± 0.5646 N=32
Mean ± SEM of column J	2.148 ± 0.07192 N=30
Difference between means	5.213 ± 0.5692
95% confidence interval	4.053 to 6.373
R squared	0.7239
F test to compare variances	
F,DFn, Dfd	65.75, 31, 29
P value	< 0.0001
P value summary	***
Are variances significantly different?	Yes

Cherry vs Cherry+L

	Data Set-A
Table Analyzed	Fig 7B
Column A	Cherry
vs	vs
Column B	Cherry+LMB
Unpaired t test	
P value	0.1892
P value summary	ns
Are means signif. different? (P < 0.05)	No
One- or two-tailed P value?	Two-tailed
t, df	t=1.328 df=59
How big is the difference?	
Mean ± SEM of column A	2.289 ± 0.06768 N=30
Mean ± SEM of column B	2.415 ± 0.06632 N=31
Difference between means	-0.1258 ± 0.09475
95% confidence interval	-0.3154 to 0.06374
R squared	0.02903

F test to compare variances	
F,DFn, Dfd	1.008, 29, 30
P value	0.9816
P value summary	ns
Are variances significantly different?	No

Figure S2B - Raw data

This file can be opened by GraphPad Prism (version 5.00 or later).

This file contains 1 data tables and 0 info tables:

- Fig S2B

Fig S2B

dTIS11-V5 NT	dTIS11-V5 LMB	dTIS11 Δ 414-436-V5 NT	dTIS11 Δ 414-436-V5 LMB	dTIS11 Δ 254-436-V5 NT	dTIS11 Δ 254-436-V5 LMB	dTIS11 Δ 1-110-V5 NT	dTIS11 Δ 1-110-V5 LMB
0,86	2,38	0,68	2,61	1,02	1,95	2,01	1,35
0,79	1,27	0,55	2,23	1,18	2,51	1,38	2,42
0,86	2,38	0,77	1,81	1,01	1,98	2,26	1,33
0,59	1,35	0,76	4,91	0,98	4,75	1,22	2,05
0,7	2,1	0,59	2,15	0,73	2,54	1,94	1,81
0,87	1,28	0,78	1,63	0,8	2,73	2,04	2,02
0,9	1,66	0,67	1,87	0,71	1,7	1,3	1,69
0,93	1,79	0,61	2,8	0,65	3,38	1,56	2,93
0,73	2,51	0,63	1,23	0,78	1,38	1,53	1,84
0,76	1,64	0,67	1,29	0,82	1,7	1,55	2,83
0,6	3,95	0,77	1,2	0,69	2,23	1,1	1,92
0,72	3,62	0,97	2,29	0,92	1,84	1,22	1,28
0,75	2,06	0,94	1,62	0,87	1,51	1,48	1,43
0,71	2,79	0,89	2,57	0,95	2	1,73	2,62
0,75	1,63	0,68	1,35	0,85	2,22	1,77	1,91
0,81	2,03	0,95	2,31	1,07	2,01	1,34	1,85
0,67	3,43	0,76	1,79	0,91	2,08	1,03	0,87
0,99	1,3	0,82	1,46	1,1	2,45	1,41	1,89
0,91	1,19	0,66	3,32	1,37	2,09	1,1	1,43
0,64	2,12	0,75	1,6	0,98	2,15	1,07	2,73
0,77	2,33	0,56	1,89	1,04	1,71	1,54	2,24
0,82	1,41	0,93	1,59	0,87	2,57	1,14	2,9
0,9	2,24	0,73	2,43	0,86	2,53	1,59	3,03
0,78	1,41	0,74	1,7	0,76	2,3	1,34	2,59
0,69	1,11	0,74	1,86	0,82	1,96	1,2	1,34
0,69	2,07	0,69	1,82	0,89	2,2	1,62	1,35
0,85	3,34	0,71	1,41	0,8	2,57	1,23	1,74
0,75	2,06	0,7	1,62	1,41	1,61	1,48	2,53
1	1,28	0,76	3,04	0,87	1,93	1,51	1,48
0,7	1,54	0,83	3,21	0,76	2,64	1,85	2,53
0,9	1,8	0,87	1,56	0,73	1,71	1,92	3,46
0,88	1,68	0,72	1,51	0,72	2,99	0,9	1,62
0,72	1,76	0,76		0,72	1,86	1,32	
0,97	1,55	0,81		0,76	2,96	1,17	
	2,18	0,79		0,88	2,31	1,72	
	2,19				2,81	1,41	
	2,99						
	1,46						
	1,34						

	1,07<br>1,71<br>1,9						

Fig. S2B Dot plot

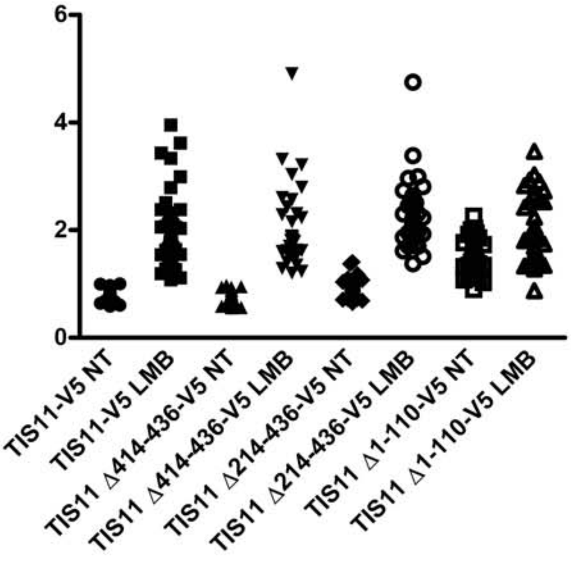

Fig. S2B Mean with SD

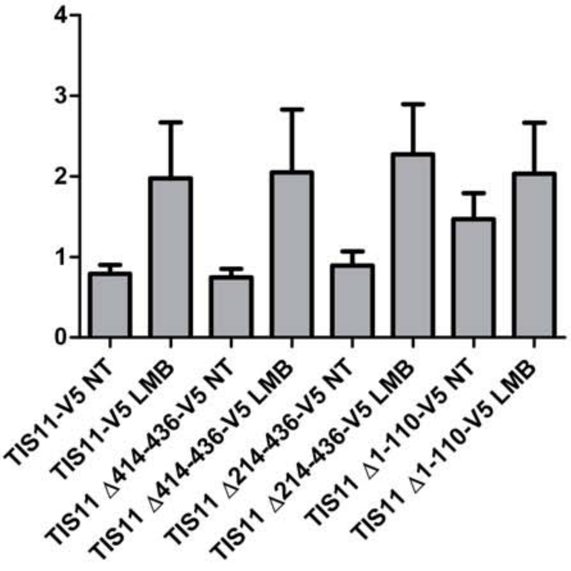

Fig. S2B Mean with SEM

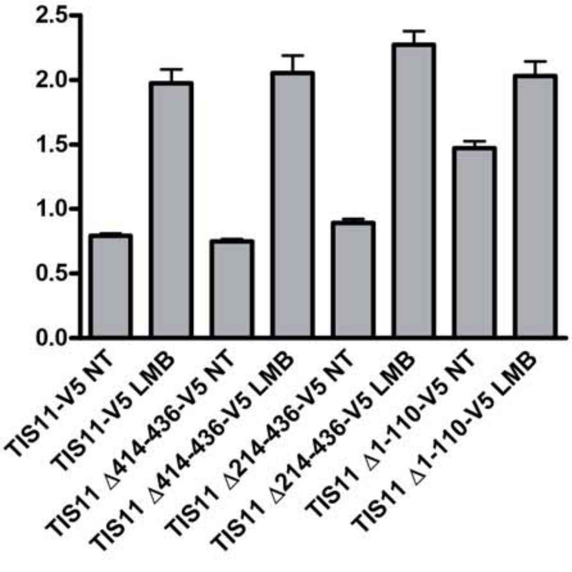

Figure S2B - Statistical analysis

This file can be opened by GraphPad Prism (version 5.00 or later).

This file contains 4 data tables and 0 info tables:

- Col. stats of Fig S2B
- WT vs delta414-436
- WT vs delta214-436
- WT vs delta1-110

Col. stats of Fig S2B

	dTIS11-V5 NT	dTIS11-V5 LMB	dTIS11 Δ 414-436-V5 NT	dTIS11 Δ 414-436-V5 LMB	dTIS11 Δ 254-436-V5 NT	dTIS11 Δ 254-436-V5 LMB	dTIS11 Δ 1-110-V5 NT	dTIS11 Δ 1-110-V5 LMB
Number of values	34	42	35	32	35	36	36	32
Minimum	0.5900	1.070	0.5500	1.200	0.6500	1.380	0.9000	0.8700
25% Percentile	0.7075	1.410	0.6800	1.568	0.7600	1.878	1.220	1.443
Median	0.7750	1.795	0.7500	1.815	0.8700	2.175	1.445	1.900
75% Percentile	0.8850	2.263	0.8100	2.400	0.9800	2.563	1.695	2.575
Maximum	1.000	3.950	0.9700	4.910	1.410	4.750	2.260	3.460
Mean	0.7929	1.974	0.7497	2.053	0.8937	2.274	1.472	2.032
Std. Deviation	0.1088	0.6987	0.1064	0.7763	0.1772	0.6210	0.3206	0.6338
Std. Error	0.01867	0.1078	0.01799	0.1372	0.02995	0.1035	0.05343	0.1120
Lower 95% CI of mean	0.7550	1.756	0.7132	1.773	0.8328	2.064	1.363	1.803
Upper 95% CI of mean	0.8309	2.192	0.7863	2.332	0.9546	2.484	1.580	2.260
KS normality test								
KS distance	0.09458	0.1220	0.1101	0.2079	0.1369	0.1223	0.08590	0.1324
P value	> 0.10	> 0.10	> 0.10	0.0012	0.0948	> 0.10	> 0.10	> 0.10
Passed normality test (alpha=0.05)?	Yes	Yes	Yes	No	Yes	Yes	Yes	Yes
P value summary	ns	ns	ns	**	ns	ns	ns	ns
Sum	26.96	82.90	26.24	65.68	31.28	81.86	52.98	65.01

WT vs delta414-436

	Data Set-A
Table Analyzed	Fig S2B
Column A	dTIS11-V5 NT
vs	vs
Column C	dTIS11 Δ 414-436-V5 NT
Mann Whitney test	
P value	0.1302
Exact or approximate P value?	Gaussian Approximation
P value summary	ns
Are medians signif. different? (P < 0.05)	No

One- or two-tailed P value?	Two-tailed
Sum of ranks in column A,C	1317 , 1099
Mann-Whitney U	468.5

WT vs delta214-436

	Data Set-A
Table Analyzed	Fig S2B
Column A	dTIS11-V5 NT
vs	vs
Column E	dTIS11 Δ 254-436-V5 NT
Mann Whitney test	
P value	0.0148
Exact or approximate P value?	Gaussian Approximation
P value summary	*
Are medians signif. different? (P < 0.05)	Yes
One- or two-tailed P value?	Two-tailed
Sum of ranks in column A,E	986.5 , 1429
Mann-Whitney U	391.5

WT vs delta1-110

	Data Set-A
Table Analyzed	Fig S2B
Column A	dTIS11-V5 NT
vs	vs
Column G	dTIS11 Δ 1-110-V5 NT
Mann Whitney test	
P value	< 0.0001
Exact or approximate P value?	Gaussian Approximation
P value summary	***
Are medians signif. different? (P < 0.05)	Yes
One- or two-tailed P value?	Two-tailed
Sum of ranks in column A,G	601.5 , 1884
Mann-Whitney U	6.500

Figure S2E - Raw data

This file can be opened by GraphPad Prism (version 5.00 or later).

This file contains 1 data tables and 0 info tables:

- **Fig S2E**

Fig S2E

Cherry	Cherry+LMB	Cherry-dTIS11 WT	Cherry-dTIS11 WT +LMB	Cherry-dTIS11 L108	Cherry-dTIS11 L108 +LMB	Cherry-dTIS11 M111A L113A	Cherry-dTIS11 M111A L113A +LMB	Cherry-dTIS11 delta98-113	Cherry-dTIS11 delta98-113 +LMB
1,79	2,12	0,81	2,39	1,68	2,35	1,83	1,51	1,58	2,23
1,87	1,72	0,71	2,31	1,28	2,42	2,28	2,4	2,02	1,7
1,98	1,85	1,05	1,93	1,72	2,39	2	1,87	1,71	1,72
1,82	2,08	0,88	2,27	1,85	2	1,69	2,74	2,33	2,33
1,85	1,82	0,71	2,26	1,38	2,22	1,75	2,62	3,21	1,91
1,83	1,68	0,8	1,91	1,83	2,51	1,79	1,56	1,85	1,57
1,52	1,41	0,69	3,02	1,82	2,36	2,28	2,55	1,76	2,08
2,24	2,39	0,69	2,26	1,53	2,74	1,78	1,38	1,65	3,04
1,46	1,76	0,89	1,78	2,4	2,26	1,88	3,28	2,18	2,09
1,81	1,61	0,82	1,81	1,53	2,52	1,99	2,2	1,38	1,59
1,94	1,77	0,79	2,46	1,58	3,7	1,98	1,56	1,28	2,21
1,73	1,74	0,77	3,08	1,42	2,4	1,8	2,69	1,24	1,96
1,83	2,17	0,65	2,7	1,54	2,47	1,51	2,22	2,04	1,69
1,98	1,6	0,98	2,28	1,96	1,42	1,44	2,47	1,01	2,24
1,88	2,02	0,88	1,95	1,67	2,06	1,27	2,61	1,95	2,14
1,8	1,81	0,83	2,85	1,79	2,46	2,03	2,47	1,69	2,51
1,78	1,88	0,87	1,87	1,39	2,57	1,73	2,24	1,89	2,23
2,42	2,25	0,7	1,7	1,24	2,17	1,31	2,52	1,68	1,91
1,97	1,38	0,81	2,22	1,59	2,27	1,65	2,37	1,71	2,47
1,71	1,55	0,66	2,44	1,74	1,85	1,63	1,41	1,62	1,82
1,53	1,37	0,86	2,21	1,63	2,29	1,9	1,83	2,41	2,5
1,76	1,39	0,87	2,59	1,52	2,09	1,66	1,54	1,59	2,28
1,91	2,33	0,75	2,17	1,28	2,38	1,76	2,46	1,75	2,58
1,99	1,95	0,7	3,2	1,94	2,18	2,16	2,54	1,61	1,93
1,78	1,8	0,84	2,03	1,69	1,93	2,27	2,52	1,79	2,58
1,8	1,67	0,84	1,91	1,86	2,22	1,9	2,16	2,86	2,05
1,46	2,01	0,9	2,5	1,61	1,64	1,86	1,28	2,1	2,53
1,57	2,13	0,99	1,84	1,58	2,44	2,24	1,4	1,68	1,97
1,78	2,34	0,88	3,01	1,41	2,64	1,94	2,39	1,95	2,39
1,8	2,15	0,79	2,94	1,32	3,09	1,64	2,21	2,36	2,27
1,97	1,87	0,79	2,01	1,82	2,54	1,8		2,92	2,65
2,17			2,49	1,6	1,99	1,73		1,46	
1,71			2,63		2,59			1,79	
			2,39		2,39				
			3,08						
			2,1						

Fig S2E Dot plot

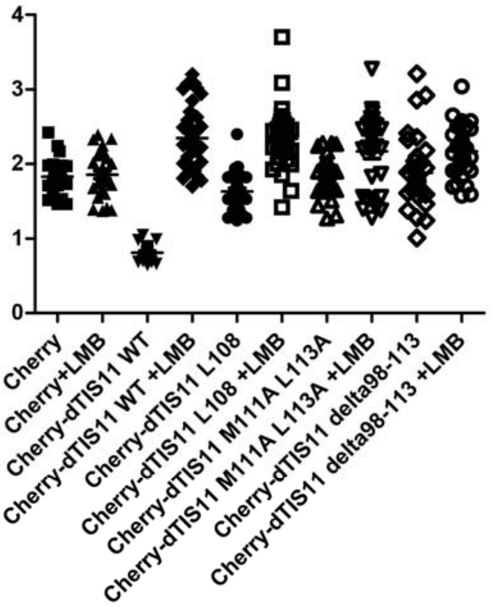

Fig S2E Mean +SD

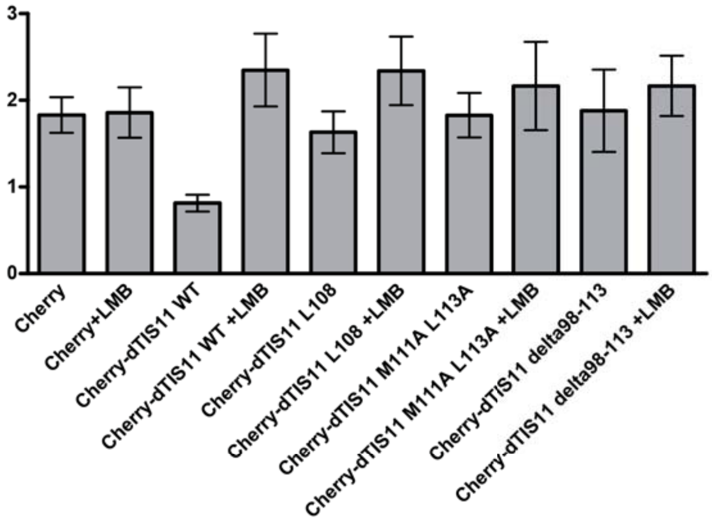

Fig S2E Mean +SEM

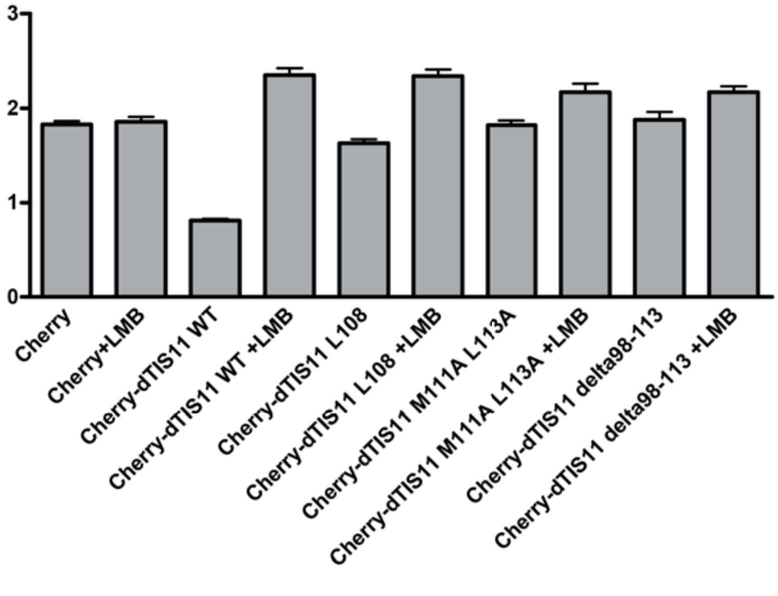

Figure S2E - Statistical analysis

This file can be opened by GraphPad Prism (version 5.00 or later).

This file contains 4 data tables and 0 info tables:

- Col Stats of Fig S2E
- WT vs L108A
- WT vs M111A L113A
- WT vs delta98-113

Col Stats of Fig S2E

	Cherry	Cherry+LMB	Cherry-dTIS11 WT	Cherry-dTIS11 WT +LMB	Cherry-dTIS11 L108	Cherry-dTIS11 L108 +LMB	Cherry-dTIS11 M111A L113A	Cherry-dTIS11 M111A L113A +LMB	Cherry-dTIS11 delta98-113	Cherry-dTIS11 delta98-113 +LMB
Number of values	33	31	31	36	32	34	32	30	33	31
Minimum	1.460	1.370	0.6500	1.700	1.240	1.420	1.270	1.280	1.010	1.570
25% Percentile	1.745	1.670	0.7100	1.965	1.445	2.150	1.668	1.560	1.615	1.910
Median	1.810	1.820	0.8100	2.275	1.605	2.370	1.800	2.305	1.760	2.210
75% Percentile	1.955	2.120	0.8800	2.620	1.813	2.513	1.988	2.525	2.070	2.470
Maximum	2.420	2.390	1.050	3.200	2.400	3.700	2.280	3.280	3.210	3.040
Mean	1.832	1.859	0.8129	2.350	1.631	2.340	1.828	2.167	1.880	2.167
Std. Deviation	0.2052	0.2920	0.09788	0.4218	0.2422	0.3953	0.2572	0.5097	0.4763	0.3491
Std. Error	0.03573	0.05245	0.01758	0.07030	0.04282	0.06780	0.04546	0.09306	0.08292	0.06270
Lower 95% CI of mean	1.759	1.752	0.7770	2.207	1.544	2.202	1.735	1.976	1.711	2.039
Upper 95% CI of mean	1.904	1.966	0.8488	2.492	1.719	2.478	1.920	2.357	2.049	2.295
KS normality test										
KS distance	0.1291	0.08385	0.1115	0.09342	0.07876	0.1457	0.09628	0.1614	0.1509	0.06832
P value	> 0.10	> 0.10	> 0.10	> 0.10	> 0.10	0.0650	> 0.10	0.0446	0.0543	> 0.10
Passed normality test (alpha=0.05)?	Yes	Yes	Yes	Yes	Yes	Yes	Yes	No	Yes	Yes
P value summary	ns	ns	ns	ns	ns	ns	ns	*	ns	ns
Sum	60.44	57.62	25.20	84.59	52.20	79.55	58.48	65.00	62.05	67.17

WT vs L108A

	Data Set-A
Table Analyzed	Data 1
Column C	WT
vs	vs
Column E	L108
Mann Whitney test	
P value	< 0.0001
Exact or approximate P value?	Gaussian Approximation
P value summary	***
Are medians signif. different? (P < 0.05)	Yes
One- or two-tailed P value?	Two-tailed
Sum of ranks in column C,E	496 , 1520
Mann-Whitney U	0.0000

WT vs M111A L113A

	Data Set-A
Table Analyzed	Data 1
Column C	WT
vs	vs
Column G	M111A L113A

Mann Whitney test	
P value	< 0.0001
Exact or approximate P value?	Gaussian Approximation
P value summary	***
Are medians signif. different? (P < 0.05)	Yes
One- or two-tailed P value?	Two-tailed
Sum of ranks in column C,G	496 , 1520
Mann-Whitney U	0.0000

WT vs delta98-113

	Data Set-A
Table Analyzed	Fig S2E
Column C	WT
vs	vs
Column I	delta98-113
Mann Whitney test	
P value	< 0.0001
Exact or approximate P value?	Gaussian Approximation
P value summary	***
Are medians signif. different? (P < 0.05)	Yes
One- or two-tailed P value?	Two-tailed
Sum of ranks in column C,I	497 , 1583
Mann-Whitney U	1.000

Figure S3C - Raw data

This file can be opened by GraphPad Prism (version 5.00 or later).

This file contains 1 data tables and 0 info tables:

- Fig S3C

Fig S3C

dTIS11-V5 NT	dTIS11-V5 LMB	dTIS11 C179A-V5 NT	dTIS11 C179A-V5 LMB	dTIS11 C156A C179A-V5 NT	dTIS11 C156A C179A-V5 LMB
0,86	2,38	0,68	2,61	0,81	3,45
0,79	1,27	0,68	2,35	0,91	3,96
0,86	2,38	1,04	4,78	0,85	4,67
0,59	1,35	0,98	3,37	0,8	2,11
0,7	2,1	0,94	3,1	0,9	3,94
0,87	1,66	1	3,34	0,81	2,28
0,9	1,79	0,94	3,6	0,98	3,44
0,93	2,51	0,87	5,05	0,99	4,07
0,73	1,64	1,03	3,65	0,76	3,25
0,76	3,95	0,7	1,77	1,03	5,05
0,6	3,62	0,82	3,23	0,96	2,08
0,72	2,06	1,08	6,81	0,75	1,89
0,75	2,79	0,84	3,75	0,94	2,62
0,71	1,63	0,73	9,09	0,7	3,51
0,75	2,03	0,77	4,04	0,82	2,4
0,81	3,43	0,82	4,33	0,69	4,77
0,67	1,3	0,96	3,49	0,75	3,59
0,99	1,19	0,89	6,53	0,87	2,8
0,91	2,12	0,73	3,18	0,9	2,93
0,64	2,33	0,89	4,1	0,82	4,05
0,77	1,41	0,88	2,26	0,93	4,35
0,82	2,24	0,8	2,89	0,94	6,67
0,9	1,41	0,76	3,87	0,64	2,63
0,78	1,11	0,71	2,27	0,72	3,13
0,69	2,07	0,99	3,83	0,69	3,24
0,69	3,34	0,68	4,24	0,92	4,2
0,85	1,28	1,03	2,84	0,71	3,95
0,75	1,54	0,93	3,52	0,84	1,77
1	1,8	1,03	4,06	0,9	4,17
0,7	1,68	1,04	4,24	0,94	4,4
0,9	1,76	0,88	3,96	0,81	7,54
0,88	1,55	0,77	2,87	0,79	6,32
0,72	2,18	0,97	2,85	0,83	4,85
0,97	2,19	0,89	3,21	1,04	3,26
	2,99	0,91	6,32		6,13
	1,46		6,65		
	1,34				
	1,07				
	1,71				
	1,9				

Fig S3C Dot plot

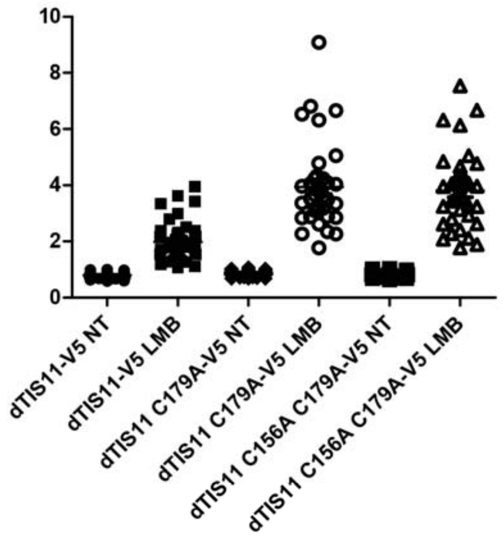

Fig S3C Mean +SD

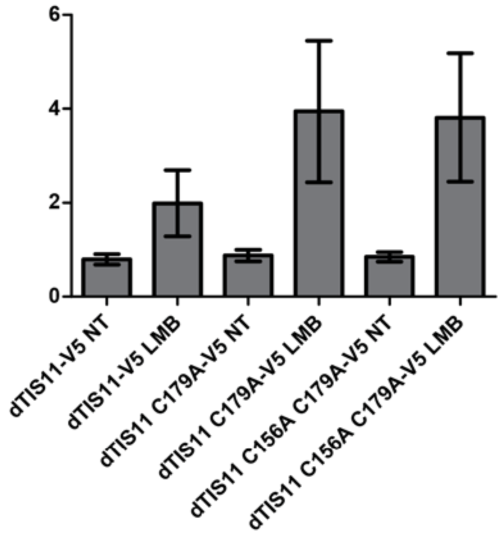

Fig S3C Mean +SEM

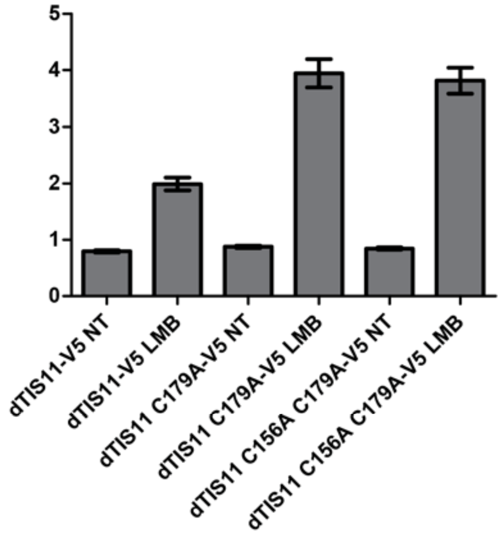

Figure S3C - Statistical analysis

This file can be opened by GraphPad Prism (version 5.00 or later).

This file contains 4 data tables and 0 info tables:

- Col. stats of Fig S3C
- WT +LMB vs delta131-213 +LMB
- WT +LMB vs C179A +LMB
- WT +LMB vs C156A C179A +LMB

Col. stats of Fig S3C

	dTIS11-V5 NT	dTIS11-V5 LMB	dTIS11 C179A-V5 NT	dTIS11 C179A-V5 LMB	dTIS11 C156A C179A-V5 NT	dTIS11 C156A C179A-V5 LMB
Number of values	34	40	35	36	34	35
Minimum	0.5900	1.070	0.6800	1.770	0.6400	1.770
25% Percentile	0.7075	1.423	0.7700	2.943	0.7575	2.800
Median	0.7750	1.795	0.8900	3.625	0.8350	3.590
75% Percentile	0.8850	2.308	0.9800	4.240	0.9325	4.400
Maximum	1.000	3.950	1.080	9.090	1.040	7.540
Mean	0.7929	1.989	0.8760	3.946	0.8453	3.813
Std. Deviation	0.1088	0.7075	0.1213	1.509	0.1043	1.368
Std. Error	0.01867	0.1119	0.02050	0.2515	0.01789	0.2312
Lower 95% CI of mean	0.7550	1.763	0.8343	3.435	0.8089	3.343
Upper 95% CI of mean	0.8309	2.215	0.9177	4.456	0.8817	4.283
KS normality test						
KS distance	0.09458	0.1303	0.09460	0.2051	0.1118	0.1055
P value	> 0.10	0.0848	> 0.10	0.0005	> 0.10	> 0.10
Passed normality test (alpha=0.05)?	Yes	Yes	Yes	No	Yes	Yes
P value summary	ns	ns	ns	***	ns	ns
Sum	26.96	79.56	30.66	142.1	28.74	133.5

WT +LMB vs delta131-213 +LMB

	Data Set-A
Table Analyzed	Fig S3C
Column B	dTIS11-V5 LMB
vs	vs
Column D	dTIS11 delta131-213 -V5 LMB
Mann Whitney test	

P value	0.8359
Exact or approximate P value?	Gaussian Approximation
P value summary	ns
Are medians signif. different? (P < 0.05)	No
One- or two-tailed P value?	Two-tailed
Sum of ranks in column B,D	1540 , 1310
Mann-Whitney U	680.0

WT +LMB vs C179A +LMB

	Data Set-A
Table Analyzed	Fig S3C
Column B	dTIS11-V5 LMB
vs	vs
Column F	dTIS11 C179A-V5 LMB
Mann Whitney test	
P value	< 0.0001
Exact or approximate P value?	Gaussian Approximation
P value summary	***
Are medians signif. different? (P < 0.05)	Yes
One- or two-tailed P value?	Two-tailed
Sum of ranks in column B,F	928.5 , 1998
Mann-Whitney U	108.5

WT +LMB vs C156A C179A +LMB

	Data Set-A
Table Analyzed	Fig S3C
Column B	dTIS11-V5 LMB
vs	vs
Column H	dTIS11 C156A C179A-V5 LMB
Mann Whitney test	
P value	< 0.0001
Exact or approximate P value?	Gaussian Approximation
P value summary	***
Are medians signif. different? (P < 0.05)	Yes
One- or two-tailed P value?	Two-tailed
Sum of ranks in column B,H	953.5 , 1897
Mann-Whitney U	133.5
